# Supplementary material for: Development and validation of multivariable machine learning algorithms to predict risk of cancer in symptomatic patients referred urgently from primary care: a diagnostic accuracy study
Source: BMJ Open. 2022 Apr 1;12(4):e053590. doi: 10.1136/bmjopen-2021-053590 (PMC8977764; doi:10.1136/bmjopen-2021-053590)
Supplement: Supplementary data [file bmjopen-2021-053590supp001.pdf]

Supplementary Materials

Table of Contents

Test Performance Characteristics 1
Table S1 1
Table S2 2
Clinical Utility Plots 3
Figure S1 3
Calibration 4
Figure S2 4
Univariate Analyses 5
Figure S3 6
ICD-10 Codes 7
Table S3 7
Table S4 7
Reference Costs 7
Table S5 7
Prevalence 8
Table S6 8

Test Performance Characteristics

In Tables S1 and S2, the "Threshold" column refers to the probability threshold that is applied to the test result for a given pathway in order to get the test performance characteristics given in the corresponding row of the table.

Table S1: Test validation set performance characteristics. Aim: 20% rule-out

Pathway	Threshold	AUC (95% CI)	NPV (95% CI)	TNR (95% CI)	FNR (95% CI)	Sensitivity (95% CI)	Specificity (95% CI)	PPV (95% CI)
Breast	0.0174	0.8007 (0.7750 – 0.8255)	0.9936 (0.9883 – 0.9981)	0.2036 (0.1926 – 0.2143)	0.0224 (0.0067 – 0.0404)	0.9776 (0.9596 – 0.9933)	0.2036 (0.1926 – 0.2143)	0.0672 (0.0601 – 0.0747)
Lower GI	0.0343	0.6798 (0.6566 – 0.7029)	0.9823 (0.9762 – 0.9877)	0.2002 (0.1921 – 0.2081)	0.0652 (0.0457 – 0.0865)	0.9348 (0.9135 – 0.9543)	0.2002 (0.1921 – 0.2081)	0.0609 (0.0559 – 0.0660)
Upper GI	0.0284	0.7323 (0.7008 – 0.7627)	0.9880 (0.9806 – 0.9946)	0.2017 (0.1901 – 0.2137)	0.0420 (0.0196 – 0.0677)	0.9580 (0.9323 – 0.9804)	0.2017 (0.1901 – 0.2137)	0.0653 (0.0576 – 0.0732)
Gynaecological	0.0392	0.8124 (0.7779 – 0.8459)	0.9895 (0.9799 – 0.9979)	0.2040 (0.1871 – 0.2209)	0.0282 (0.0058 – 0.0538)	0.9718 (0.9462 – 0.9942)	0.2040 (0.1871 – 0.2209)	0.0852 (0.0732 – 0.0980)
Urological	0.1062	0.7590 (0.7414 – 0.7757)	0.9525 (0.9358 – 0.9680)	0.2002 (0.1864 – 0.2141)	0.0319 (0.0215 – 0.0432)	0.9681 (0.9568 – 0.9785)	0.2002 (0.1864 – 0.2141)	0.2751 (0.2609 – 0.2900)
Lung	0.0876	0.7376 (0.6938 – 0.7797)	0.9630 (0.9281 – 0.9924)	0.2031 (0.1704 – 0.2331)	0.0327 (0.0067 – 0.0636)	0.9673 (0.9364 – 0.9933)	0.2031 (0.1704 – 0.2331)	0.2249 (0.1934 – 0.2571)
Haematological	0.111	0.7589 (0.7152 – 0.8006)	0.9375 (0.8795 – 0.9868)	0.2095 (0.1694 – 0.2542)	0.0303 (0.0062 – 0.0592)	0.9697 (0.9408 – 0.9938)	0.2095 (0.1694 – 0.2542)	0.3612 (0.3166 – 0.4068)
Head and Neck	0.0423	0.6996 (0.6649 – 0.7334)	0.9748 (0.9623 – 0.9858)	0.2001 (0.1862 – 0.2139)	0.0733 (0.0420 – 0.1083)	0.9267 (0.8917 – 0.9580)	0.2001 (0.1862 – 0.2139)	0.0755 (0.0657 – 0.0852)
Skin	0.0851	0.7220 (0.7057 – 0.7378)	0.9406 (0.9232 – 0.9570)	0.2002 (0.1868 – 0.2130)	0.0391 (0.0283 – 0.0507)	0.9609 (0.9493 – 0.9717)	0.2002 (0.1868 – 0.2130)	0.2796 (0.2656 – 0.2939)

Savage R, *et al. BMJ Open* 2022; 12:e053590. doi: 10.1136/bmjopen-2021-053590

BMJ Publishing Group Limited (BMJ) disclaims all liability and responsibility arising from any reliance placed on this supplemental material which has been supplied by the author(s)

Table S2: Test validation set performance characteristics. Aim: 90% rule-in

Pathway	Threshold	AUC (95% CI)	NPV (95% CI)	TNR (95% CI)	FNR (95% CI)	Sensitivity (95% CI)	Specificity (95% CI)	PPV (95% CI)
Breast	0.029	0.8007 (0.7746 – 0.8256)	0.9875 (0.9830 – 0.9916)	0.4582 (0.4450 – 0.4715)	0.0990 (0.0678 – 0.1337)	0.9010 (0.8663 – 0.9322)	0.4582 (0.4450 – 0.4715)	0.0890 (0.0793 – 0.0991)
Lower GI	0.041	0.6798 (0.6565 – 0.7029)	0.9799 (0.9745 – 0.9850)	0.2723 (0.2637 – 0.2811)	0.1006 (0.0754 – 0.1262)	0.8994 (0.8738 – 0.9246)	0.2723 (0.2637 – 0.2811)	0.0642 (0.0587 – 0.0697)
Upper GI	0.041	0.7323 (0.7012 – 0.7625)	0.9831 (0.9763 – 0.9893)	0.3363 (0.3227 – 0.3503)	0.0992 (0.0641 – 0.1389)	0.9008 (0.8611 – 0.9359)	0.3363 (0.3227 – 0.3503)	0.0732 (0.0644 – 0.0822)
Gynaecological	0.05	0.8124 (0.7768 – 0.8462)	0.9828 (0.9746 – 0.9900)	0.4674 (0.4473 – 0.4879)	0.1073 (0.0640 – 0.1553)	0.8927 (0.8447 – 0.9360)	0.4674 (0.4473 – 0.4879)	0.1134 (0.0972 – 0.1303)
Urological	0.148	0.7590 (0.7417 – 0.7762)	0.9191 (0.9035 – 0.9336)	0.3548 (0.3379 – 0.3710)	0.0996 (0.0818 – 0.1183)	0.9004 (0.8817 – 0.9182)	0.3548 (0.3379 – 0.3710)	0.3044 (0.2878 – 0.3208)
Lung	0.134	0.7376 (0.6939 – 0.7796)	0.9431 (0.9120 – 0.9702)	0.3625 (0.3238 – 0.3987)	0.0915 (0.0482 – 0.1392)	0.9085 (0.8608 – 0.9518)	0.3625 (0.3238 – 0.3987)	0.2541 (0.2178 – 0.2906)
Haematological	0.189	0.7589 (0.7143 – 0.7999)	0.9118 (0.8633 – 0.9509)	0.4330 (0.3807 – 0.4849)	0.0909 (0.0506 – 0.1412)	0.9091 (0.8588 – 0.9494)	0.4330 (0.3807 – 0.4849)	0.4249 (0.3722 – 0.4759)
Head and Neck	0.047	0.6996 (0.6648 – 0.7339)	0.9751 (0.9644 – 0.9847)	0.2733 (0.2579 – 0.2885)	0.0991 (0.0619 – 0.1393)	0.9009 (0.8607 – 0.9381)	0.2733 (0.2579 – 0.2885)	0.0804 (0.0703 – 0.0911)
Skin	0.141	0.7220 (0.7060 – 0.7380)	0.9236 (0.9100 – 0.9367)	0.3905 (0.3745 – 0.4068)	0.0999 (0.0829 – 0.1175)	0.9001 (0.8825 – 0.9171)	0.3905 (0.3745 – 0.4068)	0.3230 (0.3067 – 0.3392)

Savage R, *et al. BMJ Open* 2022; 12:e053590. doi: 10.1136/bmjopen-2021-053590

Clinical Utility Plots

Figure S1 shows negative predictive value (NPV) against the specificity, i.e. the proportion of patients ruled out, for each pathway. This shows the trade-off for a given pathway between avoiding erroneously ruling out patients who in fact have cancer (high NPV is better) vs the proportion of patients referred who are ruled out of the pathway.

Bootstrap resampling with replacement with 1000 bootstraps was used to generate 95% and 68% confidence intervals on NPV. The solid line marks the median, the dark grey band indicates the 68% confidence interval, and the light grey band indicates the 95% confidence interval.

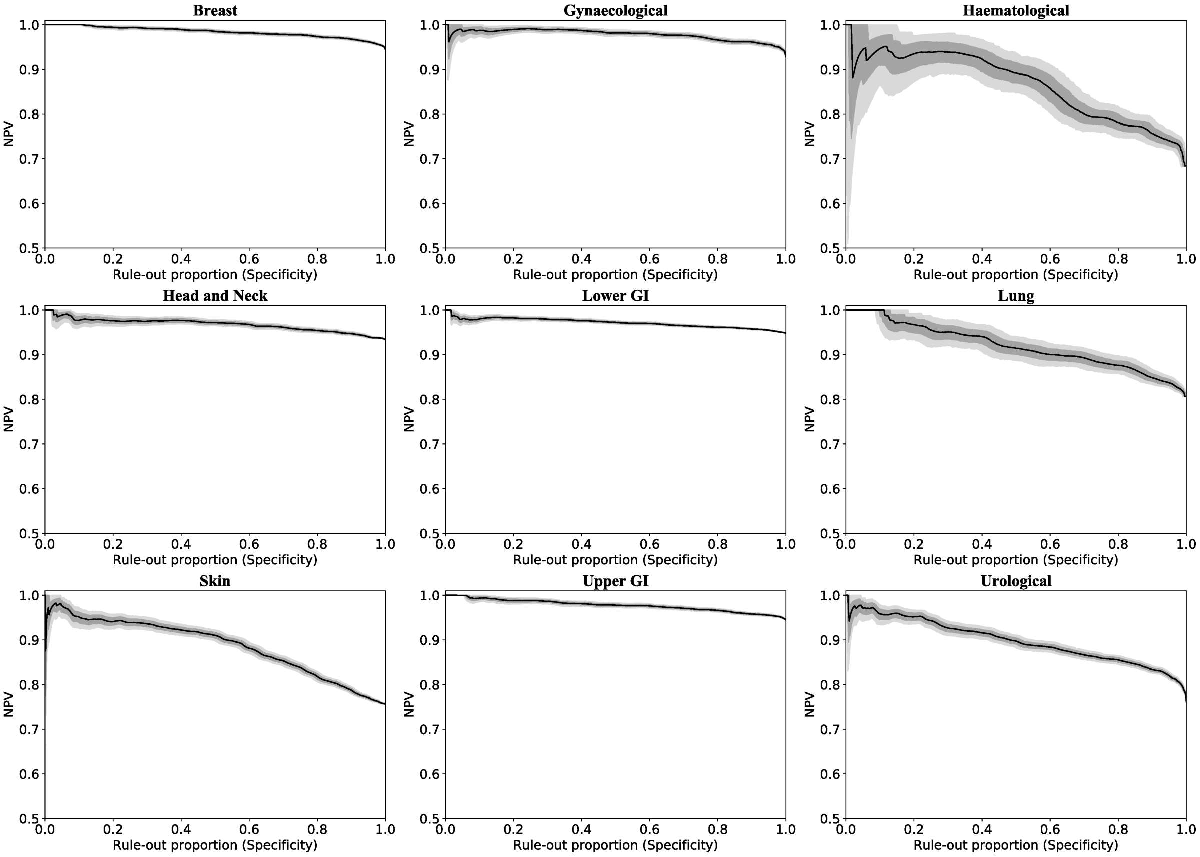

Figure S1: Plots of Negative Predictive Ability against specificity for each pathway. Light and dark grey bands indicate 68% and 95% confidence intervals. See text for details.

Savage R, *et al. BMJ Open* 2022; 12:e053590. doi: 10.1136/bmjopen-2021-053590

Calibration

Figure S2 shows calibration curves for validation set predictions by the algorithms for each pathway, calculated using equal occupancy bins. Good calibration means that the algorithm results can be interpreted as being the probability of a given patient having cancer and is indicated by the points lying along the dashed diagonal line.

The error bars show the 95% binomial proportion confidence interval, calculated using the Wilson score with continuity correction. The log loss for each pathway is also included.

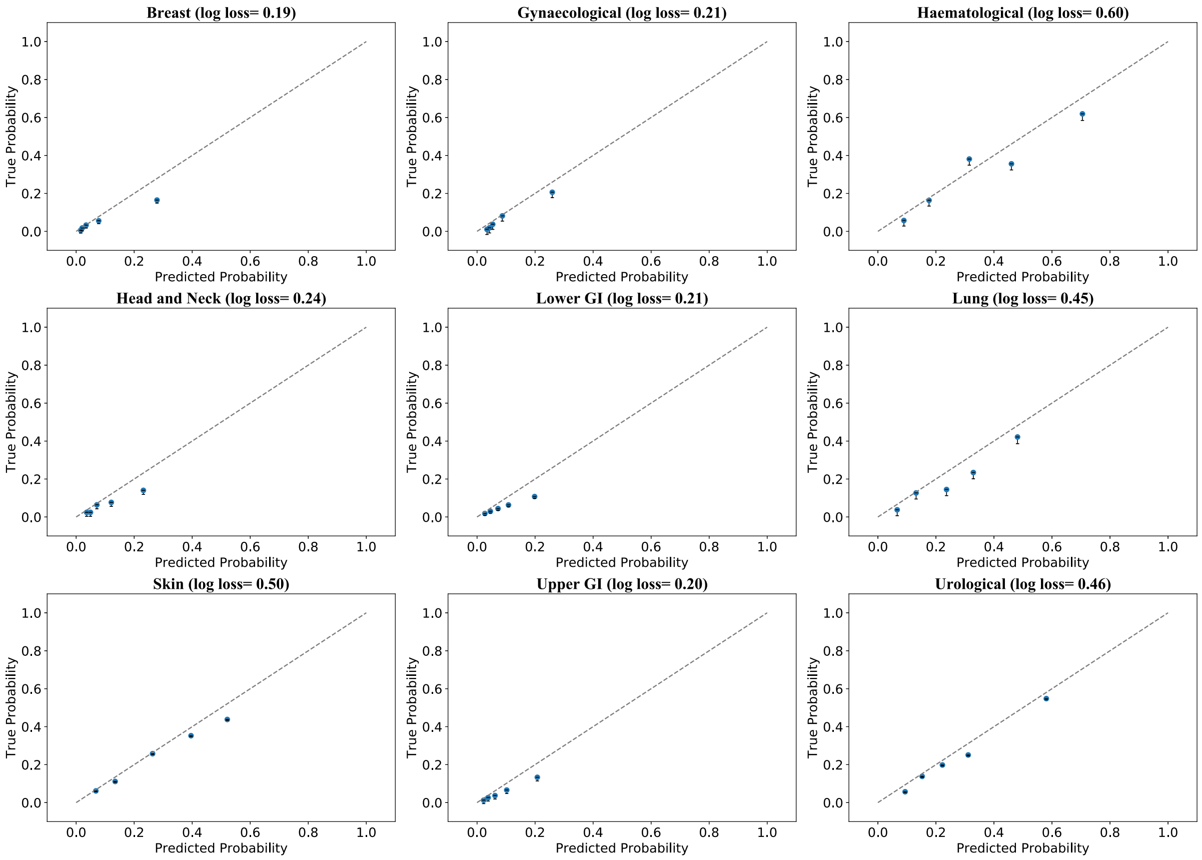

Figure S2: Plots of calibration curves per pathway. Dashed grey line indicates perfect calibration. See text for details.

Univariate Analyses

Validation set predicted probabilities were generated using the nine algorithms. For each input data feature, ROC AUCs were calculated for cases restricted to those for which the feature data was available, whereby the feature was used as the predictor and the binary cancer flag as the outcome. ROC AUCs were also calculated using the probabilities predicted by the algorithm, with identical restriction of cases applied to allow direct comparison. The difference between the algorithm ROC AUC and the single-feature ROC AUC was then calculated for each feature, ΔAUC.

Using this process, ΔAUCs were calculated for each feature and each pathway-specific algorithm. Bootstrap resampling with replacement with 10000 bootstraps was used to generate 95% confidence intervals on ΔAUC, where both the algorithm ROC AUC and single-feature ROC AUC were calculated on the same bootstrap samples.

Figure S3 shows the median ΔAUCs as black circles with 95% confidence intervals, for each feature and each pathway. Any features with data for less than one hundred patients for a given pathway were removed from the plot for that pathway. Arrows indicate that a confidence interval extends outside the plot area, in the direction of the arrow. The number of cancers and the number of cases were annotated for each feature at the bottom of the plot area. These are in the format "# cancers/# cases". An asterisk was appended to feature names for which the 95% confidence interval does not intersect the line $\Delta AUC = 0$. The feature names are assigned according to the category into which the blood test falls—"FBC" for blood counts, "Bio" for biochemistry, and "TM" for tumour markers—with numbers assigned arbitrarily but consistently across the subplots.

Savage R, *et al. BMJ Open* 2022; 12:e053590. doi: 10.1136/bmjopen-2021-053590

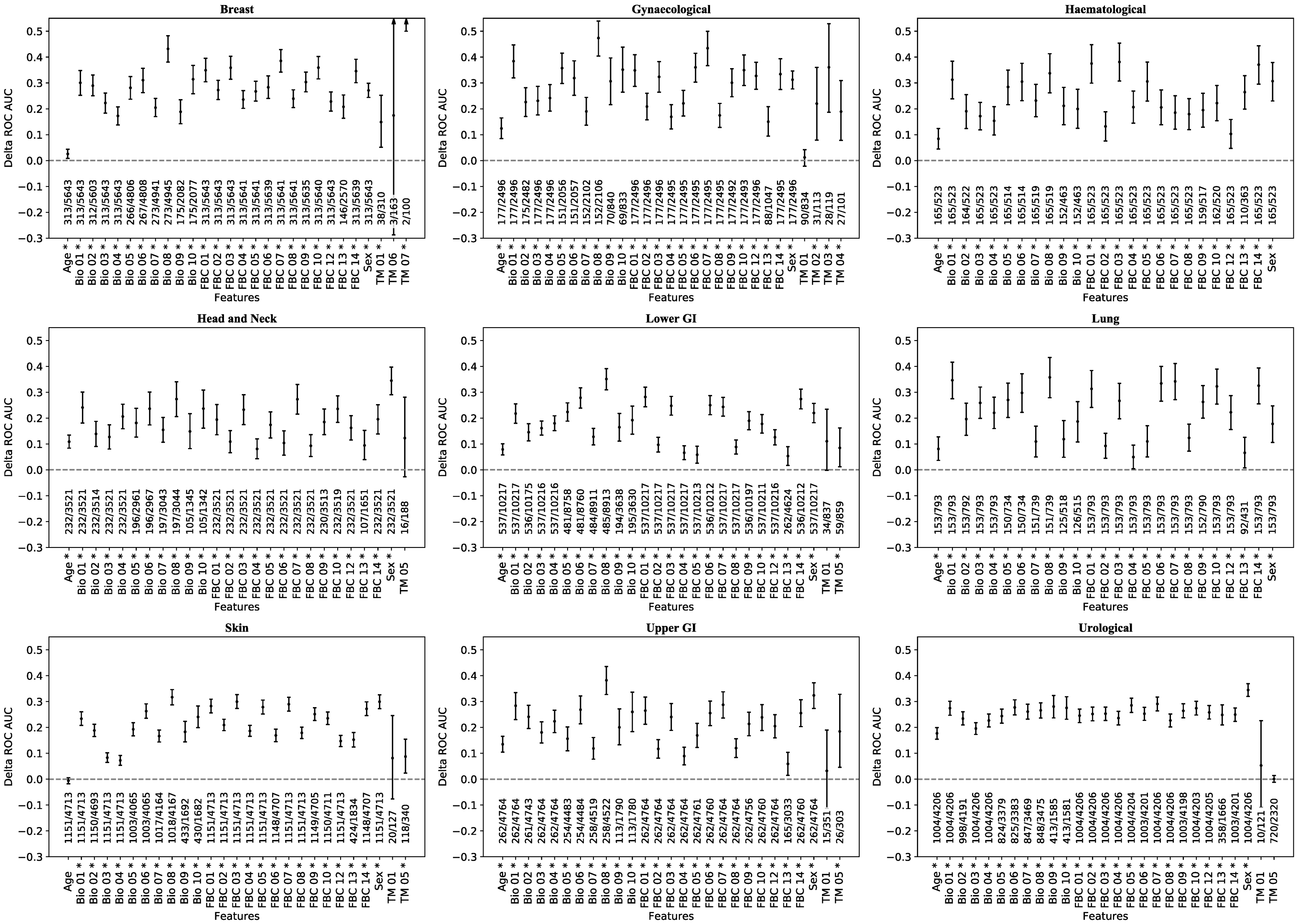

Figure S3: Plots of ΔAUC per feature per pathway. The vertical confidence intervals show the difference between ROC AUC performance for the algorithm and those that one obtains from using an individual analyte. See text for details.

Savage R, *et al. BMJ Open* 2022; 12:e053590. doi: 10.1136/bmjopen-2021-053590

ICD-10 Codes

Table S3: ICD-10 codes designated as "cancer" for the algorithms

ICD-10 code	ICD-10 text
C00-C14	Malignant neoplasms of lip, oral cavity and pharynx
C15-C26	Malignant neoplasms of digestive organs
C30-C39	Malignant neoplasms of respiratory and intrathoracic organs
C40-C41	Malignant neoplasms of bone and articular cartilage
C43-C44	Melanoma and other malignant neoplasms of skin
C45-C49	Malignant neoplasms of mesothelial and soft tissue
C50-C50	Malignant neoplasm of breast
C51-C58	Malignant neoplasms of female genital organs
C60-C63	Malignant neoplasms of male genital organs
C64-C68	Malignant neoplasms of urinary tract
C69-C72	Malignant neoplasms of eye, brain and other parts of central nervous system
C73-C75	Malignant neoplasms of thyroid and other endocrine glands
D00	Carcinoma in situ of oral cavity, oesophagus and stomach
D01	Carcinoma in situ of other and unspecified digestive organs
D02	Carcinoma in situ of middle ear and respiratory system
D03	Melanoma in situ
D04	Carcinoma in situ of skin
D05	Carcinoma in situ of breast
D07	Carcinoma in situ of other and unspecified genital organs
D09	Carcinoma in situ of other and unspecified sites

Table S4: ICD-10 codes designated as "benign" for the algorithms

ICD-10 code	ICD-10 text
D06	Carcinoma in situ of cervix uteri
D10-D36	Benign neoplasms
D37-D48	Neoplasms of uncertain or unknown behaviour

Reference Costs

Table S5 shows the reference costs for the analytes that are used as inputs to the algorithms. These costs, from the 2018-2019 reference schedule, were also used for health economics that have been performed and will be published separately.

Table S5: NHS reference costs, 2018-2019

Item	Category	Cost (2018-19 Ref Schedule)
Full Blood Counts	Haematology	£3.00
Urea & Electrolytes	Clinical Biochemistry	£1.00
CA125	Clinical Biochemistry	£1.00
CA19-9	Clinical Biochemistry	£1.00
Carcinoembryonic Antigen	Clinical Biochemistry	£1.00
CA15-3	Clinical Biochemistry	£1.00
PSA	Clinical Biochemistry	£1.00
Alpha Fetoprotein	Clinical Biochemistry	£1.00
Human Chorionic Gonadotrophin	Clinical Biochemistry	£1.00
C-Reactive Protein	Clinical Biochemistry	£1.00
Liver Function Tests	Clinical Biochemistry	£1.00
Phlebotomy	-	£4.00
**Total NHS Costs**	-	**£17.00**

Savage R, *et al. BMJ Open* 2022; 12:e053590. doi: 10.1136/bmjopen-2021-053590

Prevalence
Table S6 shows the prevalences, by pathway, for the whole cohort of patients 2011-19, including those excluded from the analyses. A comparison with Table 2 shows differences between the overall prevalences and those for the included patients, highlighting possible sources of spectrum bias. Typical prevalences for the 2WW pathways in NHSE are given for 2009-10 and 2019-20 in Smith et al. [main paper reference 17]. The right hand most column corresponds to the cancer outcomes used in the analyses in this paper, and we note that these are typically somewhat higher than 2WW prevalence rates due to the inclusion of any cancer diagnosis up to 12 months after the referral date. To illustrate this, the middle column shows the cancer prevalence when the diagnoses of the cohort of patients are restricted to only those found via the 2WW pathways, and within 62 days of referral.

Table S6: Cancer prevalence for whole cohort of patients 2011-19, including those excluded from the analyses, for two examples of diagnosis inclusion criterion. See text for details.

Pathway	Cancer prevalence (%) Restricted diagnoses (see text)	Cancer prevalence (%) All diagnoses (see text)
Breast	6.8	8.0
Lower GI	7.1	11.5
Upper GI	10.6	15.4
Gynaecological	11.3	14.3
Urological	25.0	30.6
Lung	30.0	40.4
Haematological	33.1	38.3
Head and Neck	8.8	12.6
Skin	19.4	22.3

Savage R, *et al. BMJ Open* 2022; 12:e053590. doi: 10.1136/bmjopen-2021-053590
