# Supplementary material for: Development and validation of multivariable machine learning algorithms to predict risk of cancer in symptomatic patients referred urgently from primary care: a diagnostic accuracy study
Source: BMJ Open. 2022 Apr 1;12(4):e053590. doi: 10.1136/bmjopen-2021-053590 (PMC8977764; doi:10.1136/bmjopen-2021-053590)
Supplement: Author's manuscript [file bmjopen-2021-053590.draft_revisions.pdf]

BMJ Open is committed to open peer review. As part of this commitment we make the peer review history of every article we publish publicly available.

When an article is published we post the peer reviewers' comments and the authors' responses online. We also post the versions of the paper that were used during peer review. These are the versions that the peer review comments apply to.

The versions of the paper that follow are the versions that were submitted during the peer review process. They are not the versions of record or the final published versions. They should not be cited or distributed as the published version of this manuscript.

BMJ Open is an open access journal and the full, final, typeset and author-corrected version of record of the manuscript is available on our site with no access controls, subscription charges or pay-per-view fees (http://bmjopen.bmj.com).

If you have any questions on BMJ Open's open peer review process please email info.bmjopen@bmj.com

BMJ Open

Development and validation of multivariable machine learning algorithms to predict risk of cancer in symptomatic patients referred urgently from primary care

Journal:	*BMJ Open*
Manuscript ID	bmjopen-2021-053590
Article Type:	Original research
Date Submitted by the Author:	03-Jun-2021
Complete List of Authors:	Savage, Richard; PinPoint Data Science Ltd,<br>Messenger, Mike<br>Neal, Richard<br>Ferguson, Rosie<br>Johnston, Colin<br>Lloyd, Katherine L<br>Neal, Matthew D<br>Sansom, Nigel<br>Selby, Peter<br>Sharma, Nisha<br>Shinkins, Bethany<br>Skinner, Jim R<br>Tully, Giles<br>Duffy, Sean<br>Hall, Geoff
Keywords:	Health informatics < BIOTECHNOLOGY & BIOINFORMATICS, ONCOLOGY, STATISTICS & RESEARCH METHODS

SCHOLARONE™
Manuscripts

I, the Submitting Author has the right to grant and does grant on behalf of all authors of the Work (as defined in the below author licence), an exclusive licence and/or a non-exclusive licence for contributions from authors who are: i) UK Crown employees; ii) where BMJ has agreed a CC-BY licence shall apply, and/or iii) in accordance with the terms applicable for US Federal Government officers or employees acting as part of their official duties; on a worldwide, perpetual, irrevocable, royalty-free basis to BMJ Publishing Group Ltd ("BMJ") its licensees and where the relevant Journal is co-owned by BMJ to the co-owners of the Journal, to publish the Work in this journal and any other BMJ products and to exploit all rights, as set out in our licence.

The Submitting Author accepts and understands that any supply made under these terms is made by BMJ to the Submitting Author unless you are acting as an employee on behalf of your employer or a postgraduate student of an affiliated institution which is paying any applicable article publishing charge ("APC") for Open Access articles. Where the Submitting Author wishes to make the Work available on an Open Access basis (and intends to pay the relevant APC), the terms of reuse of such Open Access shall be governed by a Creative Commons licence – details of these licences and which Creative Commons licence will apply to this Work are set out in our licence referred to above.

Other than as permitted in any relevant BMJ Author's Self Archiving Policies, I confirm this Work has not been accepted for publication elsewhere, is not being considered for publication elsewhere and does not duplicate material already published. I confirm all authors consent to publication of this Work and authorise the granting of this licence.

For peer review only

Development and validation of multivariable machine learning algorithms to predict risk of cancer in symptomatic patients referred urgently from primary care

Richard S Savage[1* +], Mike Messenger[2,5*], Richard D Neal[2,5*], Rosie Ferguson[1], Colin Johnston[3], Katherine L Lloyd[1], Matthew D Neal[1], Nigel Sansom[1], Peter Selby[2,4,5], Nisha Sharma[3], Bethany Shinkins[2,5], Jim R Skinner[1], Giles Tully[1], Sean Duffy[3**], Geoff Hall[2,3,5]**

(1) PinPoint Data Science Ltd, (2) University of Leeds, (3) Leeds Teaching Hospitals Trust, (4) Chair of the PinPoint Scientific Advisory Board, (5) NIHR MedTech and In Vitro Diagnostic Co-Operative Leeds
* Joint lead author (ORCID ID: 0000-0001-6025-1571), ** Joint last author

+ Corresponding author (Richard S Savage, rich.savage@pinpointdatascience.com)

For peer review only

Abstract

Objectives: To develop and validate tests to assess the risk of any cancer for patients referred to the NHS Urgent Suspected Cancer (Two Week Wait, 2WW) clinical pathways.

Setting: Primary and secondary care, one participating regional centre.

Participants: Retrospective analysis of data from 371,799 consecutive 2WW referrals in the Leeds region from 2011-2019. The development cohort was composed of 224,669 consecutive patients with an urgent suspected cancer referral in Leeds between January 2011 and December 2016. The diagnostic algorithms developed were then externally validated on a similar consecutive sample of 147,130 patients (between January 2017 and December 2019). All such patients over the age of 18 with a minimum set of blood counts and biochemistry measurements available were included in the cohort.

Primary and secondary outcome measures: sensitivity, specificity, NPV, PPV, ROC curve AUC, calibration curves

Results: We present results for two clinical use-cases. In use-case 1, the algorithms identify 20% of patients who do not have cancer and may not need an urgent 2WW referral. In use-case 2, they identify 90% of cancer cases with a high probability of cancer that could be prioritised for review.

Conclusions: Combining a panel of widely available blood markers produces effective blood tests for cancer for NHS 2WW patients. The tests are affordable, and can be deployed rapidly to any NHS pathology laboratory with no additional hardware requirements.

Strengths and Limitations of this Study

The principal strengths of this work are:

- It is based on well-validated, low-cost clinical assays already available at scale in NHS pathology laboratories; the tests could therefore be deployed across the UK very rapidly, with no additional hardware requirements.
- The large numbers of cases reported, and that the performance estimates are conservative due to missing data and the historical nature of the blood measurements; prospective evaluation will not suffer from these drawbacks.

The principal limitations of this work are:

- That the development and validation was done only in one centre.
- There is a possible source of bias, in that the subset of patients who had retrospective blood data may not be representative of the overall 2WW cohort.
- We have only reported the validation on a retrospective sample; a prospective evaluation is needed.

The strengths and limitations of this work are considered in greater detail in the discussion section.

For peer review only

1 Background

A major NHS cancer policy to diagnose cancer earlier led to the introduction of Urgent Suspected Cancer referrals. These referrals are predicated on the risk of symptomatic patients having cancer.[1] Trusts assess patients within two weeks ('two-week wait' (2WW) referral). The 2WW pathways have contributed to improving outcomes; higher general practice use of referrals for suspected cancer is associated with lower mortality for the four most common types of cancer (prostate, breast, lung, and colorectal).[2]

This approach places a major strain on diagnostic services on NHS England, with over 2 million 2WW referrals annually, and a 10% year-on-year increase in referrals over the past decade.[3] This highlights an unsustainable burden on existing services, workforce and financial resources. Whilst there is variation between cancer pathways, only 7% overall of 2WW referral patients are diagnosed with cancer.[3] Many patients are therefore subject to unnecessary psychological distress, as well as being exposed to diagnostic tests which may inadvertently cause harm. Clearly there is a need to improve the efficiency of these pathways.

These challenges are exacerbated by the current COVID-19 crisis. The NHS capacity to assess 2WW referrals is reduced, and a backlog of referrals continues to build.[3,4] These unprecedented challenges urgently require new solutions. COVID-19 has presented an opportunity for GPs to permanently change how they use emerging technologies.[5]

Many biomarkers have been evaluated for their use in cancer diagnosis; however only a few are currently used in either primary or secondary care settings. A systematic mapping review identified 94 ctDNA studies alone, highlighting how much more work is required prior to clinical use.[6] Companies like GRAIL and Freenome are pursuing this, with clinical trials ongoing.[7,8] There is also evidence that signals from a range of different analytes can be usefully combined via machine learning.[9]

Using such approaches to triage cancer referrals should bring benefits to patients, health-systems and the economy. For example, a *rule-out* test for symptomatic patients, like those referred to the NHS 2WW, could identify those with very low cancer risk, allowing many patients without cancer to avoid unnecessary procedures and freeing up diagnostic capacity for those at greater risk.

The work presented in this paper addresses the top three priority areas identified by Badrick et al (2019), including: a simple, non-invasive, painless and convenient test to detect cancer early; a blood test to detect some or all cancers early that can be included into routine care; and a test that is easily accessible to General Practice.[10]

We report the development and validation of a set of machine learning algorithms to provide a calibrated risk probability of cancer (a score between zero and one, higher values indicating greater risk of cancer) for triaging symptomatic patients. A calibrated risk probability has a variety of clinical uses. This paper focuses on the two use-cases for the NHS 2WW:

Use-Case 1 - a rule-out test when patient has a very low risk of cancer, allowing initial management in primary care.

Use-Case 2 - a way of identifying patients at high risk of having cancer to fast-track them for further tests.

For peer review only

2 Methods

Methodological Design and Source of Data

This work is a single centre, retrospective diagnostic prediction study (classified as a Type 2b study by the TRIPOD statement.[11] The prediction algorithms were developed and validated on a large data set from a single geographic area, split chronologically into two independent cohorts.

The data set contained 371,799 consecutive 2WW referrals in the Leeds region from 2011-2019. The development cohort was composed of 224,669 consecutive patients with an urgent suspected cancer referral in Leeds between January 2011 and December 2016. The diagnostic algorithms developed were then externally validated on a similar consecutive sample of 147,130 patients (between January 2017 and December 2019). Both development and validation sets were selected using the same inclusion and exclusion criteria and both received the same pre-processing, consisting of removing greater-than (">") symbols from blood analyte values in the data, and setting data values with less-than ("<") values to zero. This is a simple imputation for the case where a pathology laboratory returns a result outside the reportable range. Because the chosen machine learning algorithms are not sensitive to scaling of individual variables, it was not necessary to normalise the inputs.

2.1 Participants

Patients were selected because they received a 2WW referral to Leeds Teaching Hospitals NHS Trust during the above timeframe. Referrals were included for all 2WW pathways, and all patients over the age of 18 with a minimum set of blood counts and biochemistry measurements available were included in the cohort. Occasional multiple referrals of the same patient (for example to different 2WW pathways) is expected in this data set – such instances are infrequent. Patients from all 2WW pathways were included in the development set; patients from the nine 2WW pathways at LTHT considered in this paper were included in the validation set. Validation was restricted to these nine 2WW pathways (which account for ~98% of all 2WW referrals in England) because the remaining pathways, being much smaller, did not have sufficient validation data to provide useful validation. Patients not fulfilling these criteria were excluded from the analysis. All patients were followed up to 12 months after the conclusion of their referral, or until February 2020. Patients in the validation set (i.e. referred from January 2017 onwards) only required the outcome of the 2WW referral and therefore the possibility of censoring of outcomes up to 12 months did not affect the validation results.

2.2 Outcome

The algorithms were trained to predict whether or not a patient would receive a cancer diagnosis. Outcome labels were derived from ICD10 diagnostic codes from the Leeds secondary care cancer clinical database. 'Cancer' was defined as any patient diagnosed with a malignant (ICD10 'C' codes) or in situ (appropriate subset of ICD10 'D' codes) neoplasm as the result of their referral or within the subsequent 12-month period for the purposes of model development. Diagnoses as the result of an urgent referral were used as outcomes in the validation analyses, to match the intended clinical setting. Benign neoplasms were defined as 'Not Cancer'. The full list of ICD10 codes designated as 'cancer' are in the supplementary materials.

2.3 Predictors

The variables for each patient include a full blood count, a range of biochemistry measurements, a panel of standard tumour markers, plus age and sex. All predictors were included on their natural scale (i.e. they were not normalised or dichotomised).

For peer review only

As a retrospective cohort, blood measurements were used where they were available in the database up to 90 days prior to referral or up to 14 days post referral. This was done to seek a reasonable balance between missing data and possible bias (for example if blood measurements were made after a diagnosis had been established). For example, it is risky to use blood measurements taken more than 14 days post-referral as there is an increasing chance that those bloods could have been ordered by a clinician in response to a confirmed diagnosis of cancer. In routine clinical use, all model predictors would be available at the time.

2.4 Sample Size

The protocol stated the design as predicated on a goal of achieving a Negative Predictive Value (NPV) of 0.99 or greater. If we assume that we would like to determine the size of the distance from the 2.5% centile of the NPV to the point estimate (i.e. the distance between the lower bound of the 95% confidence interval (CI) and the point estimate), we can therefore determine the number of patients required in the denominator of the NPV calculation. For a 0.05 lower CI size, we require 100 patients in the denominator; for a 0.02 lower CI size we require 300 patients in the denominator. With a design goal of achieving 20% rule-out rate, this would therefore require approximately (100)/(0.2) = 500 total cases per pathway for a 0.05 lower CI size, or (300)/(0.2) = 1500 total cases per pathway for a 0.02 lower CI size.

2.5 Management of Missing Data

Missing data is a key issue for this cohort as many patients did not have bloods in this timeframe (see Tables 1, 2). Patients were identified who had full blood counts and a minimum subset of biochemistry data, and this subset was used to train the algorithms. The core algorithms use a gradient boosting model including an inbuilt method for imputing missing data which infers from the data how to handle missing data values, by learning at each decision tree node in the ensemble which branch a missing value should be assigned to. Early work during model development showed that this inbuilt method modestly outperformed (in a statistical sense) simple imputation methods, and has the advantage of simplifying the model development somewhat.

2.6 Patient and Public Involvement

Multiple public and patient consultations have been undertaken in relation to this work, initially via the NIHR-Leeds In Vitro Diagnostics Co-Operative (Leeds MIC) Public and Patient Interaction/Engagement group, expanding to Healthwatch Leeds and Healthwatch Kirklees as well as the West Yorkshire and Harrogate Cancer Alliance and CANTEST programme patient panels. Several sessions have been held and feedback gained on the clinical use of the tests presented in this work.

2.7 Statistical Analysis Methods

The goal of the algorithms is to produce a well-calibrated prediction of the probability that a patient has cancer. The type of model required is a probabilistic classifier—a model that predicts the probabilities of a given patient belonging to one of several distinct classes.

The development set was used to identify appropriate models and calibration methods and to tune the hyperparameters for those models. Methods and hyperparameters were compared using 5-fold cross-validation. This was concluded and results locked down before validation.

The model structure selected using the development set is a combination of a gradient boosting method, followed by polynomial logistic regression (i.e. a modified version of Platt scaling) to calibrate the resulting predictions. Gradient boosting was chosen for a number of pragmatic and statistical performance reasons, including statistical performance, ability to handle input variables with wildly different distributions (eg tumour markers vs blood counts), an inbuilt method for handling missing data, and modest computational load.

For peer review only - http://bmjopen.bmj.com/site/about/guidelines.xhtml

Prior to any analysis variables were selected based on: cost and relevance, availability in NHS pathology labs and prior knowledge from medical literature that they might reasonably be expected to contain some cancer-relevant information. Variable selection in the statistical sense (i.e. using the development data set) was not carried out and the gradient boosting algorithm used in this work is able to down weight any input variables which are of lesser statistical importance (in terms of contribution to making good predictions).

The validation set was used to validate the locked-down algorithms. After this no changes were made to the algorithms, results are presented below.

3 Results

Figure 1 shows a CONSORT flow diagram for this work.

Tables 1 and 2 show the total number of cases per pathway, and the number of those cases meeting the inclusion criteria. Tables 3 and 4 show the age and sex demographics of the included patients, by pathway and by development/validation set.

Table 5 shows test performance characteristics for nine urgent referral pathways for use-case 1 (rule-out). The goal here is to successfully identify 20% of non-cancer patients (a specificity of 0.2) who are at very low risk of cancer, so that other possible causes of their symptoms can be considered rather than continuing with a 2WW referral.

Table 6 shows test performance characteristics for use-case 2 (triage), to identify patients at higher risk of cancer who would be considered for priority through the urgent referral pathway. The goal here is to successfully red-flag 90% of cancer cases (a sensitivity of 0.9) for priority investigation.

Table 1: Total Number of Cases per Pathway (2011-2019)

Pathway	2011-2016	2017-2019	Total
Breast	60673	36561	97234
Lower GI	31966	22331	54297
Upper GI	18986	11938	30924
Gynaecological	16533	11599	28132
Urological	20209	13326	33535
Lung	7607	3237	10844
Haematological	2273	1323	3596
Head and Neck	22594	14558	37152
Skin	38605	29239	67844
**Key Pathways Total**	**219446**	**144112**	**363558**
**All Pathways Total**	**224669**	**147130**	**371799**

For peer review only

Table 2: Number of Cases Meeting Bloods Criteria

Pathway	Development Set			Validation Set		
	# Cancer	# Non-cancer	Prevalence	# Cancer	# Non-cancer	Prevalence
Breast	807	7571	9.6	424	5219	7.5
Lower GI	1257	11401	9.9	856	9361	8.4
Upper GI	662	5317	11.1	428	4337	9.0
Gynaecological	407	3098	11.6	218	2278	8.7
Urological	1836	4677	28.2	1143	3063	27.2
Lung	687	1380	33.2	177	616	22.3
Haematological	403	654	38.1	180	343	34.4
Head and Neck	546	4293	11.3	346	3177	9.8
Skin	1468	3910	27.3	1287	3427	27.3

Table 3: Age Demographics

Pathway	Development Set			Validation Set		
	Age 25th percentile	Age median	Age 75th percentile	Age 25th percentile	Age median	Age 75th percentile
Breast	36	48	64	35	48	62
Lower GI	59	69	78	59	69	78
Upper GI	57	68	77	55	67	76
Gynaecological	49	57	69	46	54	66
Urological	58	68	77	59	69	78
Lung	58	69	78	57	67	76
Haematological	43	63	76	43	62	75.5
Head and Neck	47	60	72	47	59	72
Skin	52	69	80	52	69	80

For peer review only

Table 4: Sex Demographics

Pathway	Development Set		Validation Set	
	# Female (%)	# Male (%)	# Female (%)	# Male (%)
Breast	7345 (87.67)	1033 (12.33)	5146 (91.19)	497 (8.82)
Lower GI	6889 (54.42)	5769 (45.58)	5529 (54.12)	4688 (45.88)
Upper GI	3346 (55.96)	2633 (44.04)	2746 (57.63)	2019 (42.37)
Gynaecological	3505 (100.00)	0 (0.00)	2495 (99.96)	1 (0.04)
Urological	1700 (26.10)	4813 (73.90)	904 (21.49)	3302 (78.51)
Lung	947 (45.82)	1120 (54.19)	363 (45.78)	430 (54.22)
Haematological	506 (47.87)	551 (52.13)	227 (43.40)	296 (56.60)
Head and Neck	2755 (56.93)	2084 (43.07)	2080 (59.04)	1443 (40.96)
Skin	2924 (54.37)	2454 (45.63)	2614 (55.45)	2100 (44.55)

Table 5: 20% Rule-out

Pathway	Proportion of non-cancers ruled-out (specificity) (95% CI)	Negative Predictive Value (95% CI)	Sensitivity (95% CI)
Breast	0.2036 (0.1926–0.2143)	0.9936 (0.9883–0.9981)	0.9776 (0.9596 - 0.9933)
Lower GI	0.2002 (0.1921–0.2081)	0.9823 (0.9762–0.9877)	0.9348 (0.9135 - 0.9543)
Upper GI	0.2017 (0.1901–0.2137)	0.9880 (0.9806–0.9946)	0.9580 (0.9323 - 0.9804)
Gynaecological	0.2040 (0.1871–0.2209)	0.9895 (0.9799–0.9979)	0.9718 (0.9462 - 0.9942)
Urological	0.2002 (0.1864–0.2141)	0.9525 (0.9358–0.9680)	0.9681 (0.9568 - 0.9785)
Lung	0.2031 (0.1704–0.2331)	0.9630 (0.9281–0.9924)	0.9673 (0.9364 - 0.9933)
Haematological	0.2095 (0.1694–0.2542)	0.9375 (0.8795–0.9868)	0.9697 (0.9408 - 0.9938)

For peer review only

Head and Neck	0.2001 (0.1862–0.2139)	0.9748 (0.9623–0.9858)	0.9267 (0.8917 - 0.9580)
Skin	0.2002 (0.1868–0.2130)	0.9406 (0.9232–0.9570)	0.9609 (0.9493 - 0.9717)

Table 6: 90% Cancer rule-in

Pathway	Proportion of non-cancers ruled-out (i.e. not red-flagged) (specificity) (95% CI)	Positive Predictive Value (95% CI)
Breast	0.4582 (0.4450–0.4715)	0.0890 (0.0793 - 0.0991)
Lower GI	0.2723 (0.2637–0.2811)	0.0642 (0.0587 - 0.0697)
Upper GI	0.3363 (0.3227–0.3503)	0.0732 (0.0644 - 0.0822)
Gynaecological	0.4674 (0.4473–0.4879)	0.1134 (0.0972 - 0.1303)
Urological	0.3548 (0.3379–0.3710)	0.3044 (0.2878 - 0.3208)
Lung	0.3625 (0.3238–0.3987)	0.2541 (0.2178 - 0.2906)
Haematological	0.4330 (0.3807–0.4849)	0.4249 (0.3722 - 0.4759)
Head and Neck	0.2733 (0.2579–0.2885)	0.0804 (0.0703 - 0.0911)
Skin	0.3905 (0.3745–0.4068)	0.3230 (0.3067 - 0.3392)

For peer review only

4 Discussion

Summary of main findings

This paper reports the development and validation of a set of statistical machine learning algorithms based on routine laboratory blood measurements that can predict cancer outcomes for symptomatic patients referred urgently from primary care for possible cancer diagnosis.

Each algorithm is trained and validated as a test to provide decision support for one of the nine NHS 2WW pathways. Each test produces a calibrated probability that the patient on that 2WW pathway has any type of cancer. These calibrated probabilities can be used in a range of clinical contexts; in this paper we consider two principal use-cases. In use-case 1, the tests are used to rule-out patients whose risk of cancer is very low, allowing clinicians to identify patients for whom investigations of possible non-cancer causes of their symptoms might be more appropriate. In use-case 2, higher-risk patients are red-flagged so that their onwards journey through the 2WW pathway can be expedited.

Table 5 shows relevant test performance characteristics for use-case 1. With a goal of 20% rule-out and corresponding Negative Predictive Values and Sensitivity, which respectively give the proportion of test-negative results which are correct (i.e. non-cancer cases) and the proportion of cancer cases that are correctly identified as cancer.

Table 6 shows relevant test performance characteristics for use-case 2. Assuming a goal of correctly red-flagging 90% of the cancer cases and presenting the proportion of non-cancer cases that are correctly not red-flagged.

More test performance characteristics can be found in Supplementary Tables S1 and S2.

Figure 2 shows an example of stratification via a test, compared with the existing standard care pathway. In this example, 500 patients present to the breast pathway, which is overloaded and only able to see 400 of these patients within two weeks of their referral. The standard care pathway is modelled as first-come first-served, and so the proportion of patients with cancer is the same in the patients seen and the patients not seen. Using the test for stratification, the patients are stratified into high, medium and low-risk groups. Patients are then seen in risk order - in this example, all of the high-risk patients are seen, and some of the medium-risk patients are seen. Under stratification, far more of the patients with cancer are seen, and of the patients not seen, a far smaller proportion have cancer. An interactive version of this is available at https://www.pinpointdatascience.com/patient-test-stratification

4.1 Discussion of main findings within the context of the literature

This work is novel, innovative, and potentially of huge importance for the management of patients referred urgently for suspected cancer. The tests are based upon a panel of routine blood measurements that: are already in common usage in NHS laboratories; work across a range of cancers; can easily be integrated with existing NHS systems. The tests have already been integrated with Mid-Yorkshire Hospitals NHS Trust Laboratory systems.

The tests can both identify patients at higher risk of cancer, such that they can be prioritised for assessment and diagnostic investigations, while also identifying a significant proportion of patients at very low risk who may not need further investigation for suspected cancer. Patients in both groups stand to benefit, either from expedited testing, or from not being exposed to iatrogenic harm and unnecessary cancer worries. The tests can be set at different thresholds in different cancers and within different health settings, making them responsive to local needs, capacity and priorities.

For peer review only

COVID has reduced diagnostic capacity and efficiency, this test could be an effective and rapid solution at this time of crisis.

An important practical note is that the criteria for 2WW changed in 2015, reducing the risk threshold warranting an urgent referral from 5% PPV to 3% PPV (i.e. towards the end of the development cohort timeframe). The validation results therefore encompass this change in clinical practice, suggesting a certain robustness to those results.

Strengths

The principal strengths of this work are:

- It is based on well-validated, low-cost clinical assays already available at scale in NHS pathology laboratories.
- The tests could therefore be deployed across the UK very rapidly, with no additional hardware requirements.
- The tests are CE marked and are currently undergoing service evaluation in the West Yorkshire and Harrogate Cancer Alliance.
- The performance estimates are conservative due to missing data and the historical nature of the blood measurements; prospective evaluation will not suffer from these drawbacks
- Even biomarkers with limited individual performance are of value in this approach if they contribute complementary information
- The algorithms are designed to be flexible, allowing thresholds to be changed according to clinical need, for example Use-Case 2 during the COVID-19 pandemic
- The large numbers reported, the robust analysis and reporting in line with TRIPOD and PROBAST.[11,12]
- There is the potential to improve performance using the pipeline of new biomarkers being developed for diagnostic, predictive or prognostic purposes.

Limitations

The principal limitations of this work are:

- That the development and validation was done only in one centre.
- There is a possible source of bias, in that the subset of patients who had retrospective blood data may not be representative of the overall 2WW cohort.
- We have only reported the validation on a retrospective sample; a prospective evaluation is needed.
- The validation set meets the defined sample size criteria (1500 total cases) for 7 of the 9 2WW. 95% CI are provided for all results to make clear the level of uncertainty present due to sample sizes.
- The remaining (smaller) 2WW pathways as recorded in the clinical data were also considered (Testicular, Brain/CNS, Sarcomas, Children's Cancer, Acute Leukaemia, HPB, Thyroid Cancer, Renal, other cancer), but we did not develop algorithms for these as the available sample sizes were judged too small to train and validate effective models.

4.2 Implications for policy research and practice

Until we have undertaken a prospective evaluation of the performance of the algorithms it is not possible to predict how this will be used. However, we do envisage use of the tool, as part of clinical

For peer review only - http://bmjopen.bmj.com/site/about/guidelines.xhtml

triage, to both prioritise those at higher levels of risk and de-prioritise those at the very lowest levels of risk, in conjunction with appropriate safety netting. We also need to fully understand the views of patients, clinicians, and commissioners on the acceptability and utility of the tests.

Acknowledgements

We thank the following people and organisations for their contributions:

- Emmylou Laird and Claire Eckert for their help with numerous aspects of this work
- The Leeds Institute for Data Analytics, in particular Phil Chambers, for all their support in providing secure computing facilities.
- The pathology and health informatics staff of Leeds Teaching Hospitals Trust and Mid-Yorkshire Hospitals NHS Trust for their help.
- The X-Lab team for help with deployment into the NHS
- The board members and partner organisations of the Leeds Centre for Personalised Medicine and Health and the Leeds Academic Health Partnership.
- The NIHR Leeds MIC and CRUK CANTEST PPI groups, especially Pete Whetstone and Margaret Johnson.
- We thank HealthWatch Leeds and Healthwatch Kirklees for patient consultation forums

Authors' contributions

RS, MM, RN, GH, RF and SD conceptualised the study, and led on the initial protocol development. GT, RF, NPS, BS and PS contributed towards funding applications and protocol refinement. RS, MN, KL and JS developed the software and algorithms, performed the data analysis and completed the CE marking process, with clinical input from RN, SD, NS, GH and PS and methodological input from BS, CJ and MM. GH led on the provision of de-identified data, assisted by CJ and RF. RF oversaw project management. All authors contributed to the interpretation of the results, writing of the manuscript and approved the final version.

Ethics statement

Data for the analysis are retrospective and fully de-identified before being released to the study team. The work was carried out under service evaluation with the formal approval of the Leeds Teaching Hospitals Trust R&I and Data Governance Committee (ref LTHT19020), and with the specific approval of the Trust Caldicott Guardian.

Data availability

The data will not be made available to others, as it is de-identified NHS patient data.

Competing interests

RS, KL, MN, JS, NPS, GT are employed by and are shareholders in PinPoint Data Science Ltd. MM has been employed as a consultant to PinPoint Data Science Ltd in October to November 2020. Both the University of Leeds and Leeds Teaching Hospitals Trust have a royalty agreement with PinPoint Data Science Ltd, meaning that those institutions are likely to benefit financially in the event of PinPoint being commercially successful.

Funding information

Aspects of this work have been supported by awards from MRC 'Proximity to Discovery', Local Enterprise Partnership, and Innovate UK. Richard Neal, Bethany Shinkins, Geoff Hall and Michael Messenger are funded by the NIHR Leeds In Vitro Diagnostic Co-operative. PinPoint Data Science Ltd funded the data science work and time contributions of Richard Savage, Matt Neal, Kat Lloyd, Jim Skinner, Giles Tully, Nigel Sansom, and Rosie Ferguson. This research is linked to the CanTest

For peer review only

Collaborative, which is funded by Cancer Research UK [C8640/A23385], of which RDN is an Associate Director, MM was a member of Senior Faculty, and BS was part-funded

TRIPOD

This work is reported in accordance with the TRIPOD statement.

Bibliography

1. Suspected Cancer: Recognition and Referral. [Internet]. National Institute for Health and Care Excellence; 2015 [cited 2020 Jul 30]. Available from: www.nice.org.uk/guidance/ng12

2. Round T, Gildea C, Asworth M, Moller H. Association between use of urgent suspected cancer referral and mortality and stage at diagnosis: a 5-year national cohort study. Br J Gen Pract. 2020;70:e389–98.

3. Cancer Waiting Time Statistics. [Internet]. NHS England; Available from: www.england.nhs.uk/statistics/statistical-work-areas/cancer-waiting-times

4. Lai AG, Pasea L, Banerjee A, Denaxas S, Katsoulis M, Chang WH, et al. Estimating excess mortality in people with cancer and multimorbidity in the COVID-19 emergency [Internet]. Oncology; 2020 Jun [cited 2020 Sep 25]. Available from: http://medrxiv.org/lookup/doi/10.1101/2020.05.27.20083287

5. Khan N, Jones D, Grice A, Alderson S, Bradley S, Carder P, et al. A brave new world: the new normal for general practice after the COVID-19 pandemic. BJGP Open. 2020 Jun 2;bjgpopen20X101103.

6. Cree IA, Uttley L, Buckley Woods H, Kikuchi H, Reiman A, et al. The evidence base for circulating tumour DNA blood-based biomarkers for the early detection of cancer: a systematic mapping review. BMC Cancer. 2017 Dec;17(1):697.

7. Liu MC, Oxnard GR, Klein EA, Swanton C, Seiden MV, Cummings SR, et al. Sensitive and specific multi-cancer detection and localization using methylation signatures in cell-free DNA. Ann Oncol. 2020 Jun;31(6):745–59.

8. Ulz P, Perakis S, Zhou Q, Moser T, Belic J, Lazzeri I, et al. Inference of transcription factor binding from cell-free DNA enables tumor subtype prediction and early detection. Nat Commun. 2019 Dec;10(1):4666.

9. Cohen JD, Li L, Wang Y, Thoburn C, Afsari B, Danilova L, et al. Detection and localization of surgically resectable cancers with a multi-analyte blood test. Science. 2018 Feb 23;359(6378):926–30.

10. Badrick E, Cresswell K, Ellis P, Renehan AG, Crosbie EJ, Crosbie P, et al. Top ten research priorities for detecting cancer early. Lancet Public Health. 2019 Nov;4(11):e551.

For peer review only

11. Collins GS, Reitsma JB, Altman DG, Moons KG. Transparent Reporting of a Multivariable Prediction Model for Individual Prognosis or Diagnosis (TRIPOD) The TRIPOD Statement. Circulation. 2015 Jan 13;131(2):211-9.

12. Wolff RF, Moons KG, Riley RD, Whiting PF, Westwood M, Collins GS, Reitsma JB, Kleijnen J, Mallett S. PROBAST: a tool to assess the risk of bias and applicability of prediction model studies. Annals of internal medicine. 2019 Jan 1;170(1):51-8.

For peer review only

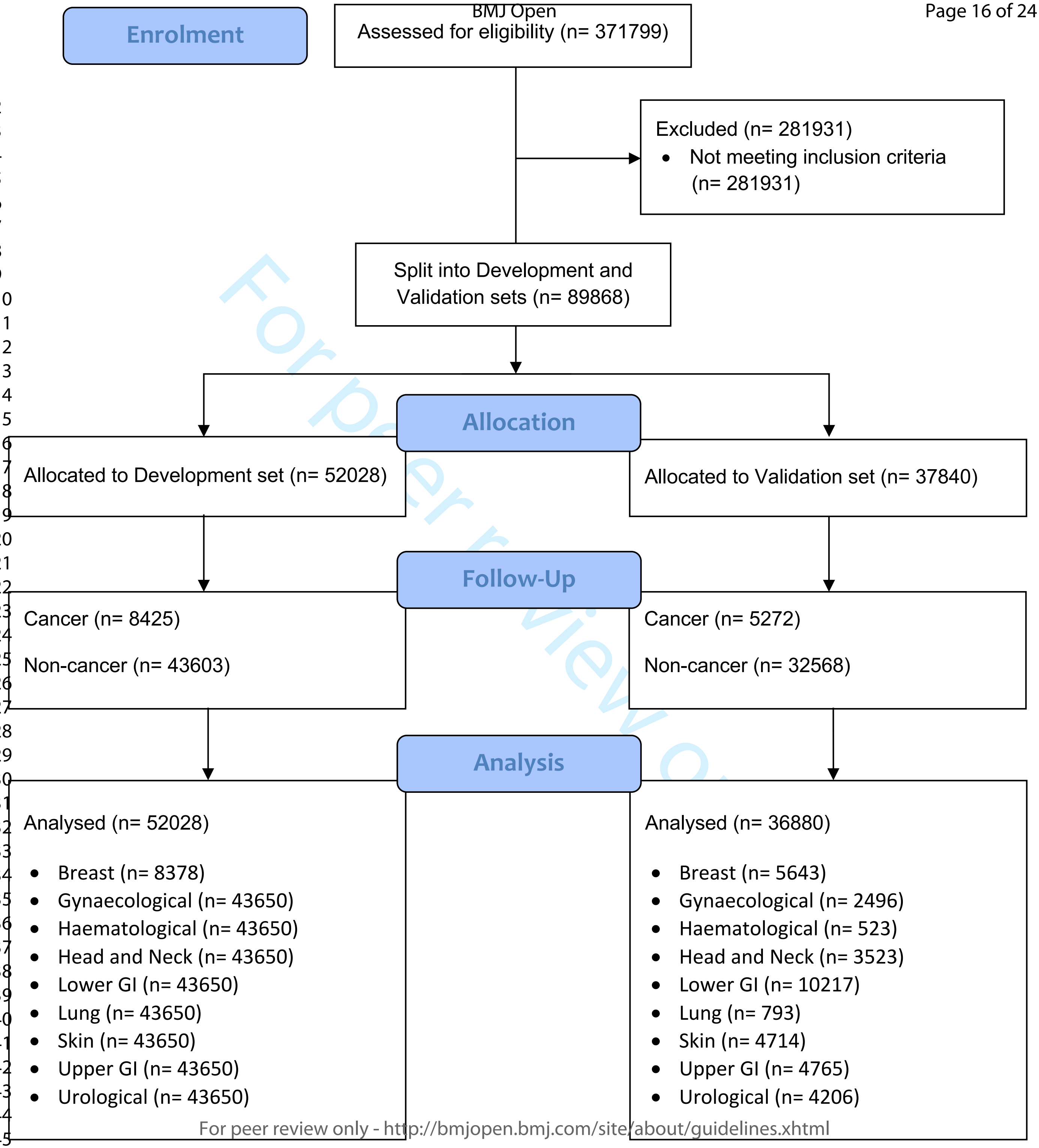

For peer review only - http://bmjopen.bmj.com/site/about/guidelines.xhtml

(Diagram adapted from CONSORT 2010 flow diagram, http://www.consort-statement.org/consort-statement/flow-diagram)

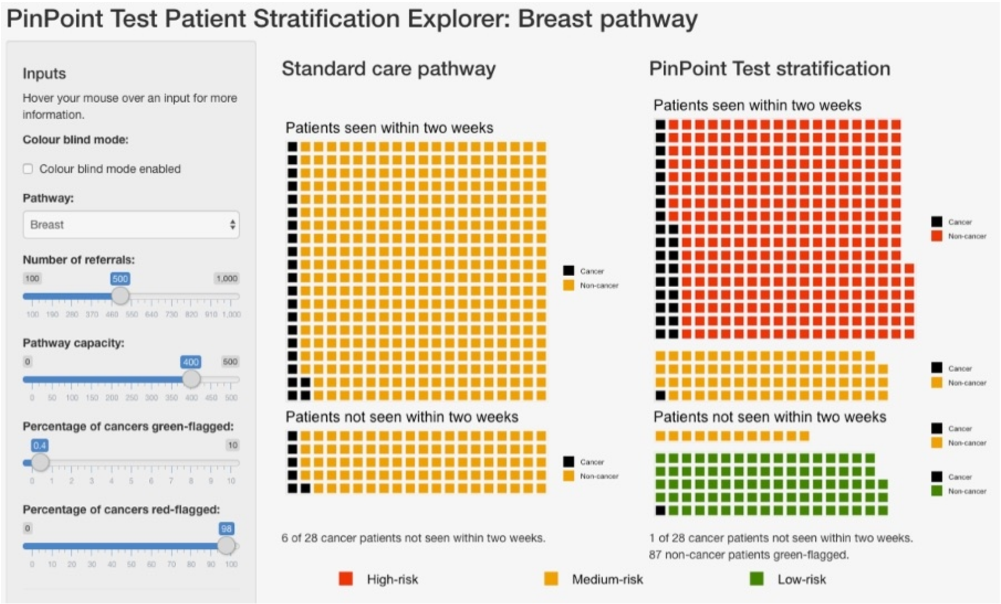

Figure 2: shows stratification of patients on the 2WW breast pathway using the relevant algorithm presented in this work, compared to the standard care pathway. Given an urgent care pathway where the number of referrals exceeds the pathway capacity to see patients within two weeks, use of the test to stratify patients into risk categories (right) leads to a larger proportion of patients with cancer being seen when compared to the standard care pathway (left), in which patients are seen on a first-come, first-served basis. Patients highlighted in red are identified as being at high-risk for cancer (red-flagged), so can be expedited for further diagnostic testing. Patients highlighted in green are identified as being at very low risk for cancer (green-flagged), allowing for initial management in primary care rather than immediate referral to secondary care.

The sliders on the left-hand side show the number of referrals, the number of patients that the pathway can handle in a given time-frame (the pathway capacity), the percentage of cancers which are green-flagged (i.e. setting a very low false negative rate, and therefore high sensitivity c.f. Table 5), and the percentage of cancers that are red-flagged (i.e. identifying cases with high-risk, so that they can be expedited for further diagnostic testing). The red-flagging slider effectively sets a sensitivity for the red-flagging process; setting sensitivity=0.9 corresponds to the results shown in Table 6. The slider for 'percentage of cancers green-flagged' can be used to set the false negative rate and see the resulting performance of the test. Collectively, this represents a possible approach to using the algorithms to improve the triage of patients referred to a 2WW pathway. An interactive version of this is available at https://www.pinpointdatascience.com/patient-test-stratification

We note that for the standard care pathway, all non-cancer patients are labelled in the same colour (yellow) to indicate that they are unstratified by the test.

159x96mm (144 x 144 DPI)

Supplementary Materials

Table of Contents

Test Performance Characteristics 1
  Table S1 1
  Table S2 2
Clinical Utility Plots 3
  Figure S1 3
Calibration 4
  Figure S2 4
Univariate Analyses 5
  Figure S3 6
ICD-10 Codes 7
  Table S3 7
  Table S4 7

Test Performance Characteristics

Table S1: Test validation set performance characteristics. Aim: 20% rule-out

Pathway	Threshold	AUC (95% CI)	NPV (95% CI)	TNR (95% CI)	FNR (95% CI)	Sensitivity (95% CI)	Specificity (95% CI)	PPV (95% CI)
Breast	0.0174	0.8007 (0.7750 – 0.8255)	0.9936 (0.9883 – 0.9981)	0.2036 (0.1926 – 0.2143)	0.0224 (0.0067 – 0.0404)	0.9776 (0.9596 – 0.9933)	0.2036 (0.1926 – 0.2143)	0.0672 (0.0601 – 0.0747)
Lower GI	0.0343	0.6798 (0.6566 – 0.7029)	0.9823 (0.9762 – 0.9877)	0.2002 (0.1921 – 0.2081)	0.0652 (0.0457 – 0.0865)	0.9348 (0.9135 – 0.9543)	0.2002 (0.1921 – 0.2081)	0.0609 (0.0559 – 0.0660)
Upper GI	0.0284	0.7323 (0.7008 – 0.7627)	0.9880 (0.9806 – 0.9946)	0.2017 (0.1901 – 0.2137)	0.0420 (0.0196 – 0.0677)	0.9580 (0.9323 – 0.9804)	0.2017 (0.1901 – 0.2137)	0.0653 (0.0576 – 0.0732)
Gynaecological	0.0392	0.8124 (0.7779 – 0.8459)	0.9895 (0.9799 – 0.9979)	0.2040 (0.1871 – 0.2209)	0.0282 (0.0058 – 0.0538)	0.9718 (0.9462 – 0.9942)	0.2040 (0.1871 – 0.2209)	0.0852 (0.0732 – 0.0980)
Urological	0.1062	0.7590 (0.7414 – 0.7757)	0.9525 (0.9358 – 0.9680)	0.2002 (0.1864 – 0.2141)	0.0319 (0.0215 – 0.0432)	0.9681 (0.9568 – 0.9785)	0.2002 (0.1864 – 0.2141)	0.2751 (0.2609 – 0.2900)
Lung	0.0876	0.7376 (0.6938 – 0.7797)	0.9630 (0.9281 – 0.9924)	0.2031 (0.1704 – 0.2331)	0.0327 (0.0067 – 0.0636)	0.9673 (0.9364 – 0.9933)	0.2031 (0.1704 – 0.2331)	0.2249 (0.1934 – 0.2571)
Haematological	0.111	0.7589 (0.7152 – 0.8006)	0.9375 (0.8795 – 0.9868)	0.2095 (0.1694 – 0.2542)	0.0303 (0.0062 – 0.0592)	0.9697 (0.9408 – 0.9938)	0.2095 (0.1694 – 0.2542)	0.3612 (0.3166 – 0.4068)
Head and Neck	0.0423	0.6996 (0.6649 – 0.7334)	0.9748 (0.9623 – 0.9858)	0.2001 (0.1862 – 0.2139)	0.0733 (0.0420 – 0.1083)	0.9267 (0.8917 – 0.9580)	0.2001 (0.1862 – 0.2139)	0.0755 (0.0657 – 0.0852)
Skin	0.0851	0.7220 (0.7057 – 0.7378)	0.9406 (0.9232 – 0.9570)	0.2002 (0.1868 – 0.2130)	0.0391 (0.0283 – 0.0507)	0.9609 (0.9493 – 0.9717)	0.2002 (0.1868 – 0.2130)	0.2796 (0.2656 – 0.2939)

For peer review only

For peer review only - http://bmjopen.bmj.com/site/about/guidelines.xhtml

Table S2: Test validation set performance characteristics. Aim: 90% rule-in

Pathway	Threshold	AUC (95% CI)	NPV (95% CI)	TNR (95% CI)	FNR (95% CI)	Sensitivity (95% CI)	Specificity (95% CI)	PPV (95% CI)
Breast	0.029	0.8007 (0.7746 – 0.8256)	0.9875 (0.9830 – 0.9916)	0.4582 (0.4450 – 0.4715)	0.0990 (0.0678 – 0.1337)	0.9010 (0.8663 – 0.9322)	0.4582 (0.4450 – 0.4715)	0.0890 (0.0793 – 0.0991)
Lower GI	0.041	0.6798 (0.6565 – 0.7029)	0.9799 (0.9745 – 0.9850)	0.2723 (0.2637 – 0.2811)	0.1006 (0.0754 – 0.1262)	0.8994 (0.8738 – 0.9246)	0.2723 (0.2637 – 0.2811)	0.0642 (0.0587 – 0.0697)
Upper GI	0.041	0.7323 (0.7012 – 0.7625)	0.9831 (0.9763 – 0.9893)	0.3363 (0.3227 – 0.3503)	0.0992 (0.0641 – 0.1389)	0.9008 (0.8611 – 0.9359)	0.3363 (0.3227 – 0.3503)	0.0732 (0.0644 – 0.0822)
Gynaecological	0.05	0.8124 (0.7768 – 0.8462)	0.9828 (0.9746 – 0.9900)	0.4674 (0.4473 – 0.4879)	0.1073 (0.0640 – 0.1553)	0.8927 (0.8447 – 0.9360)	0.4674 (0.4473 – 0.4879)	0.1134 (0.0972 – 0.1303)
Urological	0.148	0.7590 (0.7417 – 0.7762)	0.9191 (0.9035 – 0.9336)	0.3548 (0.3379 – 0.3710)	0.0996 (0.0818 – 0.1183)	0.9004 (0.8817 – 0.9182)	0.3548 (0.3379 – 0.3710)	0.3044 (0.2878 – 0.3208)
Lung	0.134	0.7376 (0.6939 – 0.7796)	0.9431 (0.9120 – 0.9702)	0.3625 (0.3238 – 0.3987)	0.0915 (0.0482 – 0.1392)	0.9085 (0.8608 – 0.9518)	0.3625 (0.3238 – 0.3987)	0.2541 (0.2178 – 0.2906)
Haematological	0.189	0.7589 (0.7143 – 0.7999)	0.9118 (0.8633 – 0.9509)	0.4330 (0.3807 – 0.4849)	0.0909 (0.0506 – 0.1412)	0.9091 (0.8588 – 0.9494)	0.4330 (0.3807 – 0.4849)	0.4249 (0.3722 – 0.4759)
Head and Neck	0.047	0.6996 (0.6648 – 0.7339)	0.9751 (0.9644 – 0.9847)	0.2733 (0.2579 – 0.2885)	0.0991 (0.0619 – 0.1393)	0.9009 (0.8607 – 0.9381)	0.2733 (0.2579 – 0.2885)	0.0804 (0.0703 – 0.0911)
Skin	0.141	0.7220 (0.7060 – 0.7380)	0.9236 (0.9100 – 0.9367)	0.3905 (0.3745 – 0.4068)	0.0999 (0.0829 – 0.1175)	0.9001 (0.8825 – 0.9171)	0.3905 (0.3745 – 0.4068)	0.3230 (0.3067 – 0.3392)

For peer review only

For peer review only - http://bmjopen.bmj.com/site/about/guidelines.xhtml

Clinical Utility Plots

Figure S1 shows negative predictive value (NPV) against the specificity, i.e. the proportion of patients ruled out, for each pathway. Bootstrap resampling with replacement with 1000 bootstraps was used to generate 95% and 68% confidence intervals on NPV. The solid line marks the median, the dark grey band indicates the 68% confidence interval, and the light grey band indicates the 95% confidence interval.

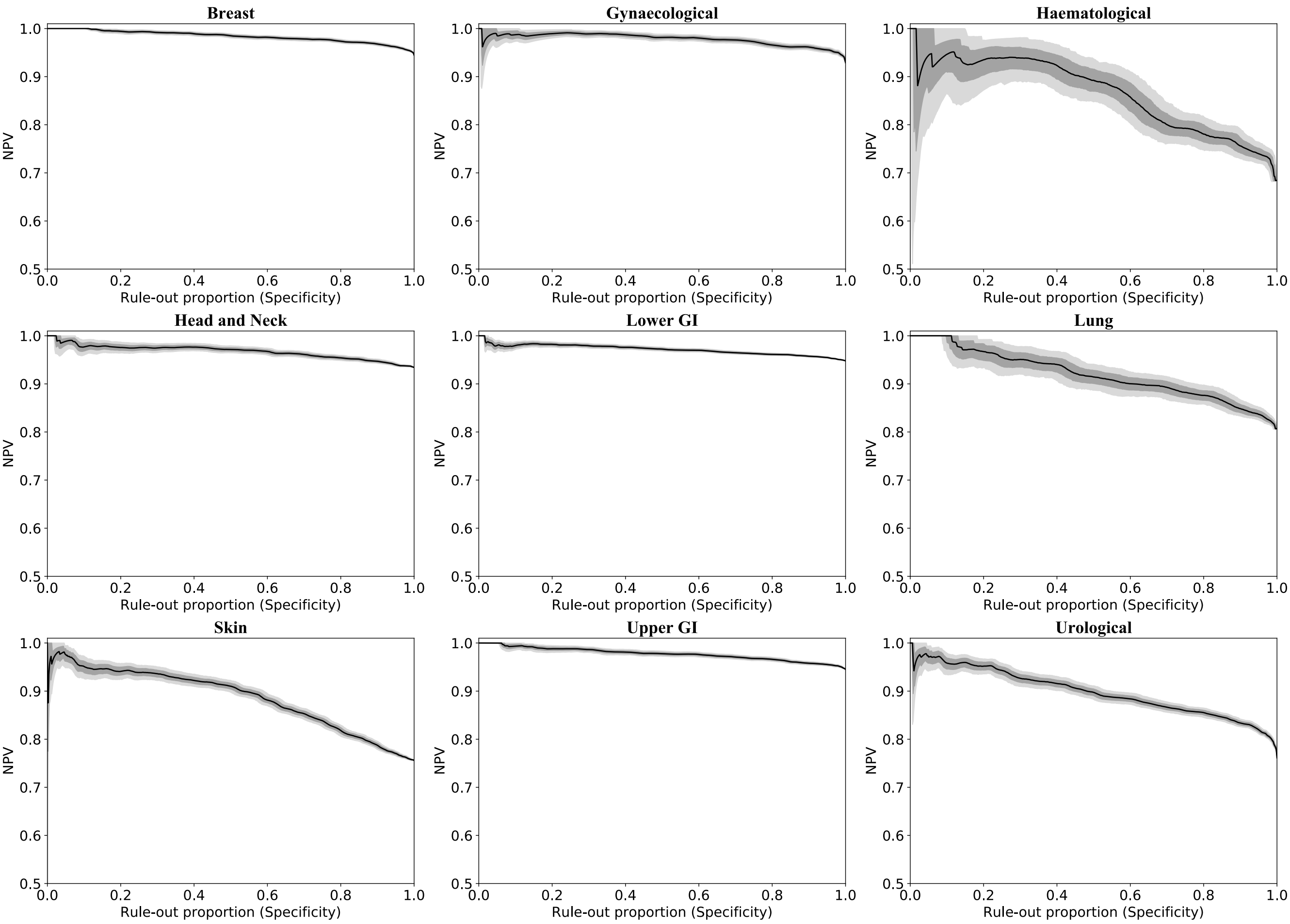

Figure S1: Plots of Negative Predictive Ability against specificity for each pathway. Light and dark grey bands indicate 68% and 95% confidence intervals. See text for details.

only

Calibration

Figure S2 shows calibration curves for validation set predictions by the algorithms for each pathway, calculated using equal occupancy bins. The error bars show the 95% binomial proportion confidence interval, calculated using the Wilson score with continuity correction. The log loss for each pathway is also included.

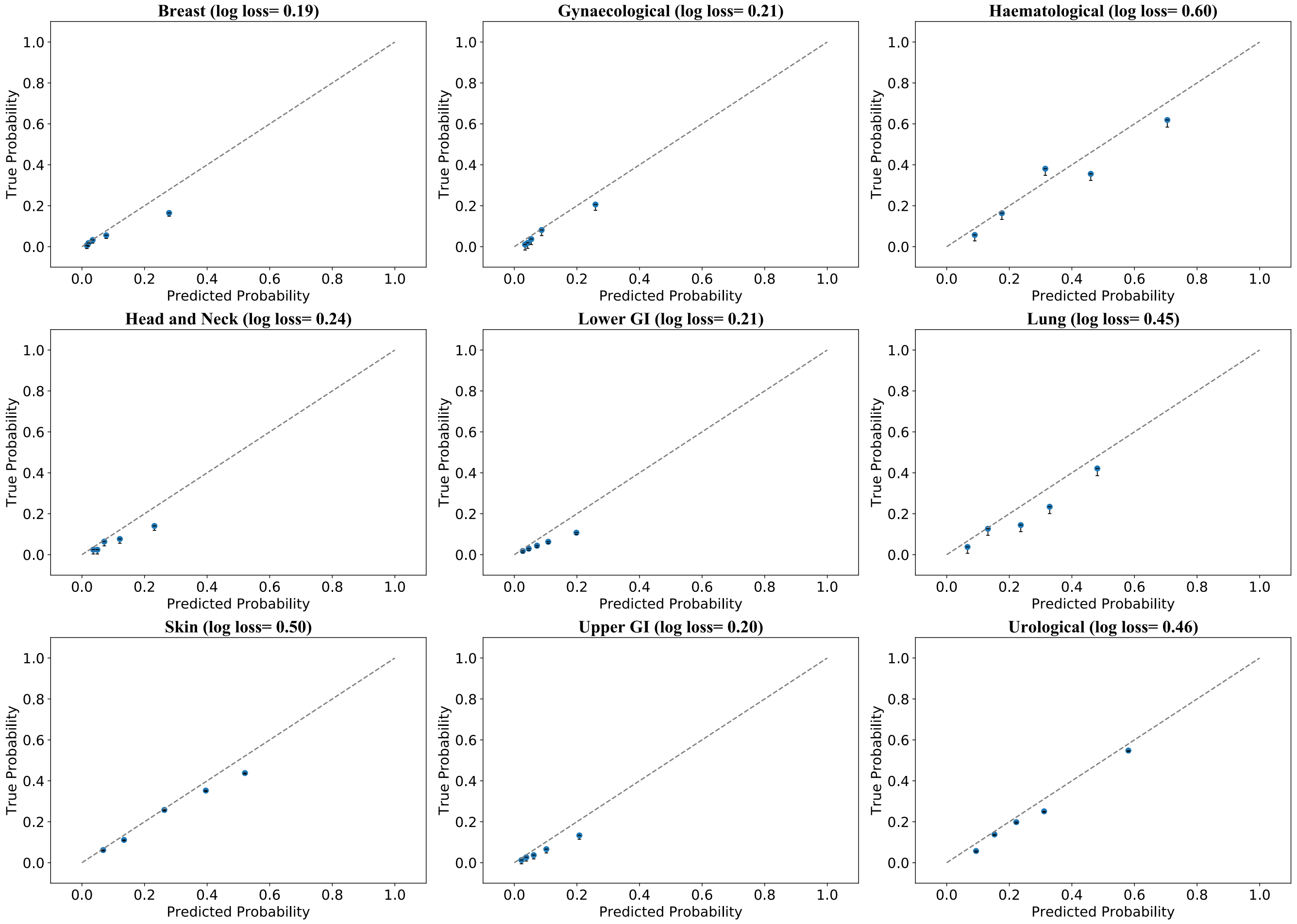

Figure S2: Plots of calibration curves per pathway. Dashed grey line indicates perfect calibration. See text for details.

For peer review only - http://bmjopen.bmj.com/site/about/guidelines.xhtml

Univariate Analyses

Validation set predicted probabilities were generated using the nine algorithms. For each input data feature, ROC AUCs were calculated for cases restricted to those for which the feature data was available, whereby the feature was used as the predictor and the binary cancer flag as the outcome. ROC AUCs were also calculated using the probabilities predicted by the algorithm, with identical restriction of cases applied to allow direct comparison. The difference between the algorithm ROC AUC and the single-feature ROC AUC was then calculated for each feature, ΔAUC.

Using this process, ΔAUCs were calculated for each feature and each pathway-specific algorithm. Bootstrap resampling with replacement with 10000 bootstraps was used to generate 95% confidence intervals on ΔAUC, where both the algorithm ROC AUC and single-feature ROC AUC were calculated on the same bootstrap samples.

Figure S3 shows the median ΔAUCs as black circles with 95% confidence intervals, for each feature and each pathway. Any features with data for less than one hundred patients for a given pathway were removed from the plot for that pathway. Arrows indicate that a confidence interval extends outside the plot area, in the direction of the arrow. The number of cancers and the number of cases were annotated for each feature at the bottom of the plot area. These are in the format "# cancers/# cases". An asterisk was appended to feature names for which the 95% confidence interval does not intersect the line $\Delta AUC = 0$. The feature names are assigned according to the category into which the blood test falls—"FBC" for blood counts, "Bio" for biochemistry, and "TM" for tumour markers—with numbers assigned arbitrarily but consistently across the subplots.

For peer review only - http://bmjopen.bmj.com/site/about/guidelines.xhtml

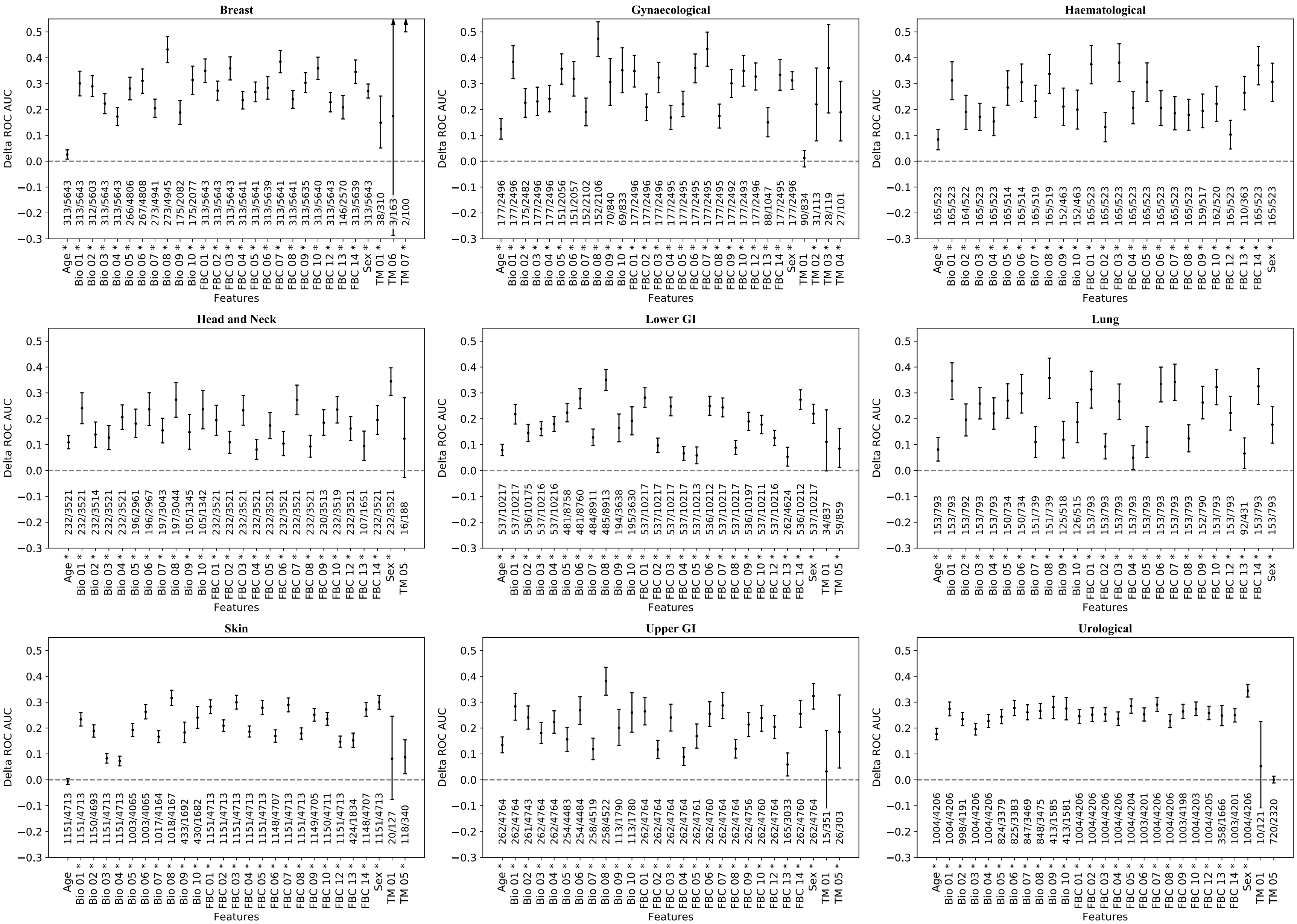

Figure S3: Plots of ΔAUC per feature per pathway. See text for details.

ICD-10 Codes

Table S3: ICD-10 codes designated as "cancer" for the algorithms

ICD-10 code	ICD-10 text
C00-C14	Malignant neoplasms of lip, oral cavity and pharynx
C15-C26	Malignant neoplasms of digestive organs
C30-C39	Malignant neoplasms of respiratory and intrathoracic organs
C40-C41	Malignant neoplasms of bone and articular cartilage
C43-C44	Melanoma and other malignant neoplasms of skin
C45-C49	Malignant neoplasms of mesothelial and soft tissue
C50-C50	Malignant neoplasm of breast
C51-C58	Malignant neoplasms of female genital organs
C60-C63	Malignant neoplasms of male genital organs
C64-C68	Malignant neoplasms of urinary tract
C69-C72	Malignant neoplasms of eye, brain and other parts of central nervous system
C73-C75	Malignant neoplasms of thyroid and other endocrine glands
D00	Carcinoma in situ of oral cavity, oesophagus and stomach
D01	Carcinoma in situ of other and unspecified digestive organs
D02	Carcinoma in situ of middle ear and respiratory system
D03	Melanoma in situ
D04	Carcinoma in situ of skin
D05	Carcinoma in situ of breast
D07	Carcinoma in situ of other and unspecified genital organs
D09	Carcinoma in situ of other and unspecified sites

Table S4: ICD-10 codes designated as "benign" for the algorithms

ICD-10 code	ICD-10 text
D06	Carcinoma in situ of cervix uteri
D10-D36	Benign neoplasms
D37-D48	Neoplasms of uncertain or unknown behaviour

For peer review only

For peer review only - http://bmjopen.bmj.com/site/about/guidelines.xhtml

TRIPOD Checklist: Prediction Model Development and Validation

Section/Topic	Item		Checklist Item	Page
**Title and abstract**				
Title	1	D;V	Identify the study as developing and/or validating a multivariable prediction model, the target population, and the outcome to be predicted.	1
Abstract	2	D;V	Provide a summary of objectives, study design, setting, participants, sample size, predictors, outcome, statistical analysis, results, and conclusions.	1
**Introduction**				
Background and objectives	3a	D;V	Explain the medical context (including whether diagnostic or prognostic) and rationale for developing or validating the multivariable prediction model, including references to existing models.	2
	3b	D;V	Specify the objectives, including whether the study describes the development or validation of the model or both.	2
**Methods**				
Source of data	4a	D;V	Describe the study design or source of data (e.g., randomized trial, cohort, or registry data), separately for the development and validation data sets, if applicable.	3
	4b	D;V	Specify the key study dates, including start of accrual; end of accrual; and, if applicable, end of follow-up.	3
Participants	5a	D;V	Specify key elements of the study setting (e.g., primary care, secondary care, general population) including number and location of centres.	3
	5b	D;V	Describe eligibility criteria for participants.	3
	5c	D;V	Give details of treatments received, if relevant.	NA
Outcome	6a	D;V	Clearly define the outcome that is predicted by the prediction model, including how and when assessed.	3
	6b	D;V	Report any actions to blind assessment of the outcome to be predicted.	3
Predictors	7a	D;V	Clearly define all predictors used in developing or validating the multivariable prediction model, including how and when they were measured.	3
	7b	D;V	Report any actions to blind assessment of predictors for the outcome and other predictors.	3
Sample size	8	D;V	Explain how the study size was arrived at.	4
Missing data	9	D;V	Describe how missing data were handled (e.g., complete-case analysis, single imputation, multiple imputation) with details of any imputation method.	4
Statistical analysis methods	10a	D	Describe how predictors were handled in the analyses.	4
	10b	D	Specify type of model, all model-building procedures (including any predictor selection), and method for internal validation.	4
	10c	V	For validation, describe how the predictions were calculated.	4
	10d	D;V	Specify all measures used to assess model performance and, if relevant, to compare multiple models.	4
	10e	V	Describe any model updating (e.g., recalibration) arising from the validation, if done.	4
Risk groups	11	D;V	Provide details on how risk groups were created, if done.	NA
Development vs. validation	12	V	For validation, identify any differences from the development data in setting, eligibility criteria, outcome, and predictors.	4
**Results**				
Participants	13a	D;V	Describe the flow of participants through the study, including the number of participants with and without the outcome and, if applicable, a summary of the follow-up time. A diagram may be helpful.	5
	13b	D;V	Describe the characteristics of the participants (basic demographics, clinical features, available predictors), including the number of participants with missing data for predictors and outcome.	4
	13c	V	For validation, show a comparison with the development data of the distribution of important variables (demographics, predictors and outcome).	6/7
Model development	14a	D	Specify the number of participants and outcome events in each analysis.	6/7
	14b	D	If done, report the unadjusted association between each candidate predictor and outcome.	supp
Model specification	15a	D	Present the full prediction model to allow predictions for individuals (i.e., all regression coefficients, and model intercept or baseline survival at a given time point).	NA
	15b	D	Explain how to the use the prediction model.	NA
Model performance	16	D;V	Report performance measures (with CIs) for the prediction model.	8/9
Model-updating	17	V	If done, report the results from any model updating (i.e., model specification, model performance).	NA
**Discussion**				
Limitations	18	D;V	Discuss any limitations of the study (such as nonrepresentative sample, few events per predictor, missing data).	12
Interpretation	19a	V	For validation, discuss the results with reference to performance in the development data, and any other validation data.	NA
	19b	D;V	Give an overall interpretation of the results, considering objectives, limitations, results from similar studies, and other relevant evidence.	11/12
Implications	20	D;V	Discuss the potential clinical use of the model and implications for future research.	11/12
**Other information**				
Supplementary information	21	D;V	Provide information about the availability of supplementary resources, such as study protocol, Web calculator, and data sets.	supp
Funding	22	D;V	Give the source of funding and the role of the funders for the present study.	4

*Items relevant only to the development of a prediction model are denoted by D, items relating solely to a validation of a prediction model are denoted by V, and items relating to both are denoted D;V. We recommend using the TRIPOD Checklist in conjunction with the TRIPOD Explanation and Elaboration document.

Development and validation of multivariable machine learning algorithms to predict risk of cancer in symptomatic patients referred urgently from primary care

Journal:	*BMJ Open*
Manuscript ID	bmjopen-2021-053590.R1
Article Type:	Original research
Date Submitted by the Author:	08-Dec-2021
Complete List of Authors:	Savage, Richard; PinPoint Data Science Ltd<br>Messenger, Mike; University of Leeds<br>Neal, Richard; University of Leeds<br>Ferguson, Rosie; PinPoint Data Science Ltd<br>Johnston, Colin; University of Leeds<br>Lloyd, Katherine L; PinPoint Data Science Ltd<br>Neal, Matthew D; PinPoint Data Science Ltd<br>Sansom, Nigel; PinPoint Data Science Ltd<br>Selby, Peter; University of Leeds<br>Sharma, Nisha; Leeds Teaching Hospitals NHS Trust<br>Shinkins, Bethany; University of Leeds<br>Skinner, Jim R; PinPoint Data Science Ltd<br>Tully, Giles; PinPoint Data Science Ltd<br>Duffy, Sean; Leeds Teaching Hospitals NHS Trust<br>Hall, Geoff; University of Leeds
**Primary Subject Heading**:	Oncology
Secondary Subject Heading:	Diagnostics, Health informatics
Keywords:	Health informatics < BIOTECHNOLOGY & BIOINFORMATICS, ONCOLOGY, STATISTICS & RESEARCH METHODS

SCHOLARONE™
Manuscripts

I, the Submitting Author has the right to grant and does grant on behalf of all authors of the Work (as defined in the below author licence), an exclusive licence and/or a non-exclusive licence for contributions from authors who are: i) UK Crown employees; ii) where BMJ has agreed a CC-BY licence shall apply, and/or iii) in accordance with the terms applicable for US Federal Government officers or employees acting as part of their official duties; on a worldwide, perpetual, irrevocable, royalty-free basis to BMJ Publishing Group Ltd ("BMJ") its licensees and where the relevant Journal is co-owned by BMJ to the co-owners of the Journal, to publish the Work in this journal and any other BMJ products and to exploit all rights, as set out in our licence.

The Submitting Author accepts and understands that any supply made under these terms is made by BMJ to the Submitting Author unless you are acting as an employee on behalf of your employer or a postgraduate student of an affiliated institution which is paying any applicable article publishing charge ("APC") for Open Access articles. Where the Submitting Author wishes to make the Work available on an Open Access basis (and intends to pay the relevant APC), the terms of reuse of such Open Access shall be governed by a Creative Commons licence – details of these licences and which Creative Commons licence will apply to this Work are set out in our licence referred to above.

Other than as permitted in any relevant BMJ Author's Self Archiving Policies, I confirm this Work has not been accepted for publication elsewhere, is not being considered for publication elsewhere and does not duplicate material already published. I confirm all authors consent to publication of this Work and authorise the granting of this licence.

For peer review only

Development and validation of multivariable machine learning algorithms to predict risk of cancer in symptomatic patients referred urgently from primary care

Richard S Savage[1* +], Mike Messenger[2,5*], Richard D Neal[2,5*], Rosie Ferguson[1], Colin Johnston[3], Katherine L Lloyd[1], Matthew D Neal[1], Nigel Sansom[1], Peter Selby[2,4,5], Nisha Sharma[3], Bethany Shinkins[2,5], Jim R Skinner[1], Giles Tully[1], Sean Duffy[3**], Geoff Hall[2,3,5**]

(1) PinPoint Data Science Ltd, (2) University of Leeds, (3) Leeds Teaching Hospitals Trust, (4) Chair of the PinPoint Scientific Advisory Board, (5) NIHR MedTech and In Vitro Diagnostic Co-Operative Leeds
* Joint lead author (ORCID ID: 0000-0001-6025-1571), ** Joint last author

+ Corresponding author (Richard S Savage, rich.savage@pinpointdatascience.com)

For peer review only

Abstract

Objectives: To develop and validate tests to assess the risk of any cancer for patients referred to the NHS Urgent Suspected Cancer (Two Week Wait, 2WW) clinical pathways.

Setting: Primary and secondary care, one participating regional centre.

Participants: Retrospective analysis of data from 371,799 consecutive 2WW referrals in the Leeds region from 2011-2019. The development cohort was composed of 224,669 consecutive patients with an urgent suspected cancer referral in Leeds between January 2011 and December 2016. The diagnostic algorithms developed were then externally validated on a similar consecutive sample of 147,130 patients (between January 2017 and December 2019). All such patients over the age of 18 with a minimum set of blood counts and biochemistry measurements available were included in the cohort.

Primary and secondary outcome measures: sensitivity, specificity, NPV, PPV, ROC curve AUC, calibration curves

Results: We present results for two clinical use-cases. In use-case 1, the algorithms identify 20% of patients who do not have cancer and may not need an urgent 2WW referral. In use-case 2, they identify 90% of cancer cases with a high probability of cancer that could be prioritised for review.

Conclusions: Combining a panel of widely available blood markers produces effective blood tests for cancer for NHS 2WW patients. The tests are affordable, and can be deployed rapidly to any NHS pathology laboratory with no additional hardware requirements.

Strengths and Limitations of this Study

The principal strengths of this work are:

- It is based on well-validated, low-cost clinical assays already available at scale in NHS pathology laboratories; the tests could therefore be deployed across the UK very rapidly, with no additional hardware requirements.
- The large numbers of cases reported, and that the performance estimates are conservative due to missing data and the historical nature of the blood measurements; prospective evaluation will not suffer from these drawbacks.

The principal limitations of this work are:

- That the development and validation was done only in one centre.
- There is a possible source of bias, in that the subset of patients who had retrospective blood data may not be representative of the overall 2WW cohort.
- We have only reported the validation on a retrospective sample; a prospective evaluation is needed.

For peer review only

1 Background

A major NHS cancer policy to diagnose cancer earlier led to the introduction of Urgent Suspected Cancer referrals. These referrals are predicated on the risk of symptomatic patients having cancer.[1] Trusts assess patients within two weeks ('two-week wait' (2WW) referral). The 2WW pathways have contributed to improving outcomes; higher general practice use of referrals for suspected cancer is associated with lower mortality for the four most common types of cancer (prostate, breast, lung, and colorectal).[2]

This approach places a major strain on diagnostic services on NHS England, with over 2 million 2WW referrals annually, and a 10% year-on-year increase in referrals over the past decade.[3] This highlights an unsustainable burden on existing services, workforce and financial resources. Whilst there is variation between cancer pathways, only 7% overall of 2WW referral patients are diagnosed with cancer.[3] Many patients are therefore subject to unnecessary psychological distress, as well as being exposed to diagnostic tests which may inadvertently cause harm. Clearly there is a need to improve the efficiency of these pathways.

These challenges are exacerbated by the current COVID-19 crisis. The NHS capacity to assess 2WW referrals is reduced, and a backlog of referrals continues to build.[3,4] These unprecedented challenges urgently require new solutions. COVID-19 has presented an opportunity for GPs to permanently change how they use emerging technologies.[5]

Many biomarkers have been evaluated for their use in cancer diagnosis; however only a few are currently used in either primary or secondary care settings. A systematic mapping review identified 94 ctDNA studies alone, highlighting how much more work is required prior to clinical use.[6] Companies like GRAIL and Freenome are pursuing this, with clinical trials ongoing.[7,8] There is also evidence that signals from a range of different analytes can be usefully combined via machine learning.[9]

Using such approaches to triage cancer referrals should bring benefits to patients, health-systems and the economy. For example, a *rule-out* test for symptomatic patients, like those referred to the NHS 2WW, could identify those with very low cancer risk, allowing many patients without cancer to avoid unnecessary procedures and freeing up diagnostic capacity for those at greater risk.

The work presented in this paper addresses the top three priority areas identified by Badrick et al (2019), including: a simple, non-invasive, painless and convenient test to detect cancer early; a blood test to detect some or all cancers early that can be included into routine care; and a test that is easily accessible to General Practice.[10]

We report the development and validation of a set of machine learning algorithms to provide a calibrated risk probability of cancer (a score between zero and one, higher values indicating greater risk of cancer) for triaging symptomatic patients. A calibrated risk probability has a variety of clinical uses. This paper focuses on the two use-cases for the NHS 2WW:

Use-Case 1 - a rule-out test when patient has a very low risk of cancer, allowing initial management in primary care.

Use-Case 2 - a way of identifying patients at high risk of having cancer to fast-track them for further tests.

For peer review only - http://bmjopen.bmj.com/site/about/guidelines.xhtml

2 Methods

Methodological Design and Source of Data

This work is a single centre, retrospective diagnostic prediction study (classified as a Type 2b study by the TRIPOD statement.[11] The prediction algorithms were developed and validated on a large data set from a single geographic area, split chronologically into two independent cohorts.

The data set contained 371,799 consecutive 2WW referrals in the Leeds region from 2011-2019. The development cohort was composed of 224,669 consecutive patients with an urgent suspected cancer referral in Leeds between January 2011 and December 2016. The diagnostic algorithms developed were then externally validated on a similar consecutive sample of 147,130 patients (between January 2017 and December 2019). Both development and validation sets were selected using the same inclusion and exclusion criteria and both received the same pre-processing, consisting of removing greater-than (">") symbols from blood analyte values in the data, and setting data values with less-than ("<") values to zero. This is a simple imputation for the case where a pathology laboratory returns a result outside the reportable range. Because the chosen machine learning algorithms are not sensitive to scaling of individual variables, it was not necessary to normalise the inputs.

2.1 Participants

Patients were selected because they received a 2WW referral to Leeds Teaching Hospitals NHS Trust during the above timeframe. Referrals were included for all 2WW pathways, and all patients over the age of 18 with a minimum set of blood counts and biochemistry measurements available were included in the cohort. Occasional multiple referrals of the same patient (for example to different 2WW pathways) is expected in this data set – such instances are infrequent, and are not modelled any differently from other referrals. While information about repeated referral could, in principle, aid the algorithm, this would make the algorithm much harder to deploy in practice as it would need reliable access to an electronic healthcare record, rather than just being linked directly to the Laboratory Information Management System (LIMS) which handles the pathology lab data flows. We have therefore avoided this on practical grounds, for the time being.

Patients from all 2WW pathways were included in the development set; patients from the nine 2WW pathways at LTHT considered in this paper were included in the validation set. The reason for including all cases in the development set is that our goal was to train algorithms that could assist with pan-cancer diagnosis, including cancer cases which have not been referred down the correct pathway. Validation was restricted to these nine 2WW pathways (which account for ~98% of all 2WW referrals in England) because the remaining pathways, being much smaller, did not have sufficient validation data to provide useful validation. Patients not fulfilling these criteria were excluded from the analysis. All patients were followed up to 12 months after the conclusion of their referral, or until February 2020. Patients in the validation set (i.e. referred from January 2017 onwards) only required the outcome of the 2WW referral and therefore the possibility of censoring of outcomes up to 12 months did not affect the validation results.

We note that differences in the blood tests GPs are likely to provide in the lead up to/as part of a 2WW referral typically vary significantly depending on pathway. This is likely to be an important factor in explaining the difference in patient inclusion rates for each pathway we see for this work (see Tables 1 and 2).

2.2 Outcome

The algorithms were trained to predict whether or not a patient would receive a cancer diagnosis. Outcome labels were derived from ICD10 diagnostic codes from the Leeds secondary care cancer

For peer review only - http://bmjopen.bmj.com/site/about/guidelines.xhtml

clinical database. 'Cancer' was defined as any patient diagnosed with a malignant (ICD10 'C' codes) or in situ (appropriate subset of ICD10 'D' codes) neoplasm as the result of their referral or within the subsequent 12-month period for the purposes of model development. Diagnoses as the result of an urgent referral were used as outcomes in the validation analyses, to match the intended clinical setting. Benign neoplasms were defined as 'Not Cancer'. The full list of ICD10 codes designated as 'cancer' are in the supplementary materials.

2.3 Predictors

The variables for each patient include a full blood count, a range of biochemistry measurements, a panel of standard tumour markers, plus age and sex. All predictors were included on their natural scale (i.e. they were not normalised or dichotomised).

As a retrospective cohort, blood measurements were used where they were available in the database up to 90 days prior to referral or up to 14 days post referral. This was done to seek a reasonable balance between missing data and possible bias (for example if blood measurements were made after a diagnosis had been established). For example, it is risky to use blood measurements taken more than 14 days post-referral as there is an increasing chance that those bloods could have been ordered by a clinician in response to a confirmed diagnosis of cancer. In routine clinical use, all model predictors would be available at the time.

2.4 Sample Size

The protocol for this work stated a goal of achieving a Negative Predictive Value (NPV) of 0.99 or greater for the rule-out use-case. Because NPVs below 0.99 are undesirable, we consider sample sizes as they impact the lower half of the 95% CI for NPV. For a 0.05 lower CI size, we require 100 total patients being ruled-out; for a 0.02 lower CI size we require 300 patients. With a design goal of achieving a 20% rule-out rate, this would therefore require approximately (100)/(0.2) = 500 total cases per pathway for a 0.05 lower CI size, or (300)/(0.2) = 1500 total cases per pathway for a 0.02 lower CI size.

The validation set meets the above sample size criteria for 7 of the 9 2WW pathways for which results are presented. The other two pathways (lung and haematological) are high prevalence pathways (see Table 2), and so it was decided to also include results for these two pathways as the 95% CI are provided for all results to make clear the level of uncertainty present due to sample sizes. The remaining (smaller) 2WW pathways as recorded in the clinical data were also considered (Testicular, Brain/CNS, Sarcomas, Children's Cancer, Acute Leukaemia, other cancer), but we did not develop algorithms for these as the available sample sizes were judged too small to train and validate effective models.

2.5 Management of Missing Data

Missing data is a key issue for this cohort as many patients did not have bloods in this timeframe (see Tables 1, 2). Patients were identified who had full blood counts and a minimum subset of biochemistry data, and this subset was used to train the algorithms. The core algorithms use a gradient boosting model including an inbuilt method for imputing missing data which infers from the data how to handle missing data values, by learning at each decision tree node in the ensemble which branch a missing value should be assigned to. Early work during model development showed that this inbuilt method modestly outperformed (in a statistical sense) simple imputation methods, and has the advantage of simplifying the model development somewhat.

2.6 Patient and Public Involvement

For peer review only

Multiple public and patient consultations have been undertaken in relation to this work, initially via the NIHR-Leeds In Vitro Diagnostics Co-Operative (Leeds MIC) Public and Patient Interaction/Engagement group, expanding to Healthwatch Leeds and Healthwatch Kirklees as well as the West Yorkshire and Harrogate Cancer Alliance and CANTEST programme patient panels. Several sessions have been held and feedback gained on the clinical use of the tests presented in this work.

2.7 Statistical Analysis Methods

The goal of the algorithms is to produce a well-calibrated prediction of the probability that a patient has cancer. The type of model required is a probabilistic classifier—a model that predicts the probabilities of a given patient belonging to one of several distinct classes.

The development set was used to identify appropriate models and calibration methods and to tune the hyperparameters for those models. Methods and hyperparameters were compared and tuned using 5-fold cross-validation. This was concluded and results locked down before validation.

The model structure selected using the development set is a combination of a core machine learning algorithm with good predictive performance(gradient boosting), plus a calibration step (polynomial logistic regression, a modified version of Platt Scaling [14]). Gradient boosting was chosen for a number of pragmatic and statistical performance reasons. It is generally seen to perform very well in comparison to other methods on structured data sets such as are used in this paper and we observed the same thing during early development work. Gradient Boosting using decision trees is also able to straightforwardly handle input variables with wildly different distributions (e.g. tumour markers vs blood counts). There are several very good Python packages available that implement gradient boosting (we use XGBoost [15] and LightGBM [16]), and these packages have built-in methods for handling missing data. Gradient boosting also has a modest computational load for both training and prediction. Platt Scaling is a standard calibration method which uses logistic regression. We have modified this to use polynomial logistic regression because we found this gave better calibration performance with the outputs of our gradient boosting algorithms.

The outcome classes for this work are significantly imbalanced, with substantially fewer cancers than non-cancers (see prevalences in Table 2). The imbalanced classes are accounted for via upweighting the importance of the cancer patients in the gradient boosting algorithms. The same weight is applied to all cancer patients, and this is tuned as a hyperparameter during the development work (i.e. using cross-validation on the development set).

Prior to any analysis variables were selected based on: cost and relevance, availability in NHS pathology labs and prior knowledge from medical literature that they might reasonably be expected to contain some cancer-relevant information. Variable selection in the statistical sense (i.e. using the development data set) was not carried out and the gradient boosting algorithm used in this work is able to down-weight any input variables which are of lesser statistical importance (in terms of contribution to making good predictions).

The validation set was used to validate the locked-down algorithms. After this no changes were made to the algorithms, results are presented below.

3 Results

Figure 1 shows a CONSORT flow diagram for this work.

For peer review only - http://bmjopen.bmj.com/site/about/guidelines.xhtml

Tables 1 and 2 show the total number of cases per pathway, and the number of those cases meeting the inclusion criteria. Tables 3 and 4 show the age and sex demographics of the included patients, by pathway and by development/validation set.

Table 5 shows test performance characteristics for nine urgent referral pathways for use-case 1 (rule-out). The goal here is to successfully identify 20% of non-cancer patients (a specificity of 0.2) who are at very low risk of cancer, so that other possible causes of their symptoms can be considered rather than continuing with a 2WW referral.

Table 6 shows test performance characteristics for use-case 2 (triage), to identify patients at higher risk of cancer who would be considered for priority through the urgent referral pathway. The goal here is to successfully red-flag 90% of cancer cases (a sensitivity of 0.9) for priority investigation.

Table 1: Total Number of Cases per Pathway (2011-2019)

Pathway	2011-2016	2017-2019	Total
Breast	60673	36561	97234
Lower GI	31966	22331	54297
Upper GI	18986	11938	30924
Gynaecological	16533	11599	28132
Urological	20209	13326	33535
Lung	7607	3237	10844
Haematological	2273	1323	3596
Head and Neck	22594	14558	37152
Skin	38605	29239	67844
**Key Pathways Total**	**219446**	**144112**	**363558**
**All Pathways Total**	**224669**	**147130**	**371799**

Table 2: Number of Cases Meeting Bloods Criteria

Pathway	Development Set			Validation Set		
	# Cancer	# Non-cancer	Prevalence	# Cancer	# Non-cancer	Prevalence
Breast	807	7571	9.6	424	5219	7.5
Lower GI	1257	11401	9.9	856	9361	8.4
Upper GI	662	5317	11.1	428	4337	9.0

For peer review only

Gynaecological	407	3098	11.6	218	2278	8.7
Urological	1836	4677	28.2	1143	3063	27.2
Lung	687	1380	33.2	177	616	22.3
Haematological	403	654	38.1	180	343	34.4
Head and Neck	546	4293	11.3	346	3177	9.8
Skin	1468	3910	27.3	1287	3427	27.3

Table 2: Details of the cases which meet the acceptance criteria for the analyses presented in this paper. Prevalence is calculated only for those cases meeting the criteria, and not for all patients entering a given pathway.

Table 3: Age Demographics

Pathway	Development Set			Validation Set		
	Age 25th percentile	Age median	Age 75th percentile	Age 25th percentile	Age median	Age 75th percentile
Breast	36	48	64	35	48	62
Lower GI	59	69	78	59	69	78
Upper GI	57	68	77	55	67	76
Gynaecological	49	57	69	46	54	66
Urological	58	68	77	59	69	78
Lung	58	69	78	57	67	76
Haematological	43	63	76	43	62	75.5
Head and Neck	47	60	72	47	59	72
Skin	52	69	80	52	69	80

Table 4: Sex Demographics

Pathway	Development Set		Validation Set	
	# Female (%)	# Male (%)	# Female (%)	# Male (%)
Breast	7345 (87.67)	1033 (12.33)	5146 (91.19)	497 (8.82)
Lower GI	6889 (54.42)	5769 (45.58)	5529 (54.12)	4688 (45.88)
Upper GI	3346 (55.96)	2633 (44.04)	2746 (57.63)	2019 (42.37)

For peer review only

Gynaecological	3505 (100.00)	0 (0.00)	2495 (99.96)	1 (0.04)
Urological	1700 (26.10)	4813 (73.90)	904 (21.49)	3302 (78.51)
Lung	947 (45.82)	1120 (54.19)	363 (45.78)	430 (54.22)
Haematological	506 (47.87)	551 (52.13)	227 (43.40)	296 (56.60)
Head and Neck	2755 (56.93)	2084 (43.07)	2080 (59.04)	1443 (40.96)
Skin	2924 (54.37)	2454 (45.63)	2614 (55.45)	2100 (44.55)

Table 5: 20% Rule-out

**Pathway**	**Proportion of non-cancers ruled-out (specificity) (95% CI)**	**Negative Predictive Value (95% CI)**	**Sensitivity (95% CI)**
Breast	0.2036 (0.1926–0.2143)	0.9936 (0.9883–0.9981)	0.9776 (0.9596 - 0.9933)
Lower GI	0.2002 (0.1921–0.2081)	0.9823 (0.9762–0.9877)	0.9348 (0.9135 - 0.9543)
Upper GI	0.2017 (0.1901–0.2137)	0.9880 (0.9806–0.9946)	0.9580 (0.9323 - 0.9804)
Gynaecological	0.2040 (0.1871–0.2209)	0.9895 (0.9799–0.9979)	0.9718 (0.9462 - 0.9942)
Urological	0.2002 (0.1864–0.2141)	0.9525 (0.9358–0.9680)	0.9681 (0.9568 - 0.9785)
Lung	0.2031 (0.1704–0.2331)	0.9630 (0.9281–0.9924)	0.9673 (0.9364 - 0.9933)
Haematological	0.2095 (0.1694–0.2542)	0.9375 (0.8795–0.9868)	0.9697 (0.9408 - 0.9938)
Head and Neck	0.2001 (0.1862–0.2139)	0.9748 (0.9623–0.9858)	0.9267 (0.8917 - 0.9580)
Skin	0.2002 (0.1868–0.2130)	0.9406 (0.9232–0.9570)	0.9609 (0.9493 - 0.9717)

For peer review only

Table 6: 90% Cancer rule-in

Pathway	Proportion of non-cancers ruled-out (i.e. not red-flagged) (specificity) (95% CI)	Positive Predictive Value (95% CI)
Breast	0.4582 (0.4450–0.4715)	0.0890 (0.0793 - 0.0991)
Lower GI	0.2723 (0.2637–0.2811)	0.0642 (0.0587 - 0.0697)
Upper GI	0.3363 (0.3227–0.3503)	0.0732 (0.0644 - 0.0822)
Gynaecological	0.4674 (0.4473–0.4879)	0.1134 (0.0972 - 0.1303)
Urological	0.3548 (0.3379–0.3710)	0.3044 (0.2878 - 0.3208)
Lung	0.3625 (0.3238–0.3987)	0.2541 (0.2178 - 0.2906)
Haematological	0.4330 (0.3807–0.4849)	0.4249 (0.3722 - 0.4759)
Head and Neck	0.2733 (0.2579–0.2885)	0.0804 (0.0703 - 0.0911)
Skin	0.3905 (0.3745–0.4068)	0.3230 (0.3067 - 0.3392)

For peer review only

4 Discussion

Summary of main findings

This paper reports the development and validation of a set of statistical machine learning algorithms based on routine laboratory blood measurements that can predict cancer outcomes for symptomatic patients referred urgently from primary care for possible cancer diagnosis.

Each algorithm is trained and validated as a test to provide decision support for one of the nine NHS 2WW pathways. Each test produces a calibrated probability that the patient on that 2WW pathway

has any type of cancer. These calibrated probabilities can be used in a range of clinical contexts; in this paper we consider two principal use-cases. In use-case 1, the tests are used to rule-out patients whose risk of cancer is very low, allowing clinicians to identify patients for whom investigations of possible non-cancer causes of their symptoms might be more appropriate. In use-case 2, higher-risk patients are red-flagged so that their onwards journey through the 2WW pathway can be expedited.

Table 5 shows relevant test performance characteristics for use-case 1. With a goal of 20% rule-out and corresponding Negative Predictive Values and Sensitivity, which respectively give the proportion of test-negative results which are correct (i.e. non-cancer cases) and the proportion of cancer cases that are correctly identified as cancer.

Table 6 shows relevant test performance characteristics for use-case 2. Assuming a goal of correctly red-flagging 90% of the cancer cases and presenting the proportion of non-cancer cases that are correctly not red-flagged.

More test performance characteristics can be found in Supplementary Tables S1 and S2.

Figure 2 shows an example of stratification via a test, compared with the existing standard care pathway. In this example, 500 patients present to the breast pathway, which is overloaded and only able to see 400 of these patients within two weeks of their referral. The standard care pathway is modelled as first-come first-served, and so the proportion of patients with cancer is the same in the patients seen and the patients not seen. Using the test for stratification, the patients are stratified into high, medium and low-risk groups. Patients are then seen in risk order - in this example, all of the high-risk patients are seen, and some of the medium-risk patients are seen. Under stratification, far more of the patients with cancer are seen, and of the patients not seen, a far smaller proportion have cancer. An interactive version of this is available at https://www.pinpointdatascience.com/patient-test-stratification

4.1 Discussion of main findings within the context of the literature

This work is novel, innovative, and potentially of huge importance for the management of patients referred urgently for suspected cancer. The tests are based upon a panel of routine blood measurements that: are already in common usage in NHS laboratories; work across a range of cancers; can easily be integrated with existing NHS systems. The tests have already been integrated with Mid-Yorkshire Hospitals NHS Trust Laboratory systems.

The tests can both identify patients at higher risk of cancer, such that they can be prioritised for assessment and diagnostic investigations, while also identifying a significant proportion of patients at very low risk who may not need further investigation for suspected cancer. Patients in both groups stand to benefit, either from expedited testing, or from not being exposed to iatrogenic harm and unnecessary cancer worries. The tests can be set at different thresholds in different cancers and within different health settings, making them responsive to local needs, capacity and priorities. COVID has reduced diagnostic capacity and efficiency, this test could be an effective and rapid solution at this time of crisis.

An important practical note is that the criteria for 2WW changed in 2015, reducing the risk threshold warranting an urgent referral from 5% PPV to 3% PPV (i.e. towards the end of the development cohort timeframe). The validation results therefore encompass this change in clinical practice, suggesting a certain robustness to those results.

For peer review only

Strengths

This work is based on well-validated, low-cost clinical assays (see Table S5) already available at scale in NHS pathology laboratories. The tests could therefore be deployed across the UK very rapidly, with no additional hardware requirements. These tests are CE marked and are currently undergoing service evaluation in the West Yorkshire and Harrogate Cancer Alliance. The use of low-cost assays means that these tests are very affordable in comparison to typical per-patient 2WW referral costs.[13]

The performance estimates are conservative due to missing data and the historical nature of the blood measurements; prospective evaluation will not suffer from these drawbacks. Even biomarkers with limited individual performance are of value in this approach if they contribute complementary information. The algorithms are designed to be flexible, allowing thresholds to be changed according to clinical need, for example Use-Case 2 during the COVID-19 pandemic. The large numbers reported, the robust analysis and reporting in line with TRIPOD and PROBAST.[11,12] There is the potential to improve performance using the pipeline of new biomarkers being developed for diagnostic, predictive or prognostic purposes.

Limitations

The development and validation was done only in one centre, albeit a large regional cancer centre. We have also only reported the validation on a retrospective sample - a prospective multi-centre evaluation is needed to provide confidence in the generalisability of the model.

We note that the validation set meets the defined sample size criteria (1500 total cases) for 7 of the 9 2WW. 95% CI are provided for all results to make clear the level of uncertainty present due to sample sizes. The remaining (smaller) 2WW pathways as recorded in the clinical data were also considered (Testicular, Brain/CNS, Sarcomas, Children's Cancer, Acute Leukaemia, other cancer), but we did not develop algorithms for these as the available sample sizes were judged too small to train and validate effective models.

There is a possible source of bias, in that the subset of patients who had retrospective blood data may not be representative of the overall 2WW cohort. Different pathways have different conventions as to what blood tests are performed as part of a 2WW referral. For example, we note that the proportion of men with a breast 2WW referral meeting the inclusion criteria (see Table 4) is unusually high compared to that which would be expected for the pathway as a whole. Many breast cancer pathways specifically ask for a panel of blood tests to be performed by GPs prior to two week wait referrals in males (for the investigation of gynaecomastia) which is not required for female referrals, suggesting bias.

The choice to use blood measurements from up to 90 days prior to and up to 14 days post-referral is also a possible source of bias. Bloods taken significantly before referral can be biased because if the patient does have cancer, any tumour could be smaller or even not yet present at the time the blood test was administered. And bloods taken post-referral begin to run the risk that the decision was taken to order the blood test using information not available at the time of referral. We have chosen this timeframe as a reasonable balance between missing data and these potential biases. We note that for both values (90 days prior, 14 days post) we performed a sensitivity analysis during

For peer review only

algorithm development where we varied these parameters and re-ran otherwise identical cross-validations. This showed that the choice of (90 days prior, 14 days post) was reasonably stable, and in particular we did not see any significant gains in algorithm performance unless the post-referral cut-off was increased past 21 days, suggesting that while that source of bias does exist, it is not a significant factor with a 14 days post-referral cut-off.

4.2 Implications for policy research and practice

Until we have undertaken a prospective evaluation of the performance of the algorithms it is not possible to predict how this will be used. However, we do envisage use of the tool, as part of clinical triage, to both prioritise those at higher levels of risk and de-prioritise those at the very lowest levels of risk, in conjunction with appropriate safety netting. We also need to fully understand the views of patients, clinicians, and commissioners on the acceptability and utility of the tests. We note that each 2WW pathway is distinct, with its own challenges and priorities, as well as differing prevalences of cancer (see e.g. Smith et al [17]) - these issues will likely require detailed consideration by all the key stakeholders on a pathway-by-pathway basis.

Acknowledgements

We thank the following people and organisations for their contributions:

- Emmylou Laird and Claire Eckert for their help with numerous aspects of this work
- The Leeds Institute for Data Analytics, in particular Phil Chambers, for all their support in providing secure computing facilities.
- The pathology and health informatics staff of Leeds Teaching Hospitals Trust and Mid-Yorkshire Hospitals NHS Trust for their help.
- The X-Lab team for help with deployment into the NHS
- The board members and partner organisations of the Leeds Centre for Personalised Medicine and Health and the Leeds Academic Health Partnership.
- The NIHR Leeds MIC and CRUK CANTEST PPI groups, especially Pete Whetstone and Margaret Johnson.
- We thank HealthWatch Leeds and Healthwatch Kirklees for patient consultation forums

Authors' contributions

RS, MM, RN, GH, RF and SD conceptualised the study, and led on the initial protocol development. GT, RF, NPS, BS and PS contributed towards funding applications and protocol refinement. RS, MN, KL and JS developed the software and algorithms, performed the data analysis and completed the CE marking process, with clinical input from RN, SD, NS, GH and PS and methodological input from BS, CJ and MM. GH led on the provision of de-identified data, assisted by CJ and RF. RF oversaw project management. All authors contributed to the interpretation of the results, writing of the manuscript and approved the final version.

Ethics statement

Data for the analysis are retrospective and fully de-identified before being released to the study team. The work was carried out under service evaluation with the formal approval of the Leeds Teaching Hospitals Trust R&I and Data Governance Committee (ref LTHT19020), and with the specific approval of the Trust Caldicott Guardian.

Data availability

For peer review only - http://bmjopen.bmj.com/site/about/guidelines.xhtml

The data will not be made available to others, as it is de-identified NHS patient data.

Competing interests

RS, KL, MN, JS, NPS, GT are employed by and are shareholders in PinPoint Data Science Ltd. MM has been employed as a consultant to PinPoint Data Science Ltd in October to November 2020. Both the University of Leeds and Leeds Teaching Hospitals Trust have a royalty agreement with PinPoint Data Science Ltd, meaning that those institutions are likely to benefit financially in the event of PinPoint being commercially successful.

Funding information

Aspects of this work have been supported by awards from MRC 'Proximity to Discovery', Local Enterprise Partnership, and Innovate UK. Richard Neal, Bethany Shinkins, Geoff Hall and Michael Messenger are funded by the NIHR Leeds In Vitro Diagnostic Co-operative. PinPoint Data Science Ltd funded the data science work and time contributions of Richard Savage, Matt Neal, Kat Lloyd, Jim Skinner, Giles Tully, Nigel Sansom, and Rosie Ferguson. This research is linked to the CanTest Collaborative, which is funded by Cancer Research UK [C8640/A23385], of which RDN is an Associate Director, MM was a member of Senior Faculty, and BS was part-funded

TRIPOD

This work is reported in accordance with the TRIPOD statement.

Bibliography

1. Suspected Cancer: Recognition and Referral. [Internet]. National Institute for Health and Care Excellence; 2015 [cited 2020 Jul 30]. Available from: www.nice.org.uk/guidance/ng12

2. Round T, Gildea C, Asworth M, Moller H. Association between use of urgent suspected cancer referral and mortality and stage at diagnosis: a 5-year national cohort study. Br J Gen Pract. 2020;70:e389–98.

3. Cancer Waiting Time Statistics. [Internet]. NHS England; Available from: www.england.nhs.uk/statistics/statistical-work-areas/cancer-waiting-times

4. Lai AG, Pasea L, Banerjee A, Denaxas S, Katsoulis M, Chang WH, et al. Estimating excess mortality in people with cancer and multimorbidity in the COVID-19 emergency [Internet]. Oncology; 2020 Jun [cited 2020 Sep 25]. Available from: http://medrxiv.org/lookup/doi/10.1101/2020.05.27.20083287

5. Khan N, Jones D, Grice A, Alderson S, Bradley S, Carder P, et al. A brave new world: the new normal for general practice after the COVID-19 pandemic. BJGP Open. 2020 Jun 2;bjgpopen20X101103.

6. Cree IA, Uttley L, Buckley Woods H, Kikuchi H, Reiman A, et al. The evidence base for circulating tumour DNA blood-based biomarkers for the early detection of cancer: a systematic mapping review. BMC Cancer. 2017 Dec;17(1):697.

For peer review only - http://bmjopen.bmj.com/site/about/guidelines.xhtml

7. Liu MC, Oxnard GR, Klein EA, Swanton C, Seiden MV, Cummings SR, et al. Sensitive and specific multi-cancer detection and localization using methylation signatures in cell-free DNA. Ann Oncol. 2020 Jun;31(6):745–59.

8. Ulz P, Perakis S, Zhou Q, Moser T, Belic J, Lazzeri I, et al. Inference of transcription factor binding from cell-free DNA enables tumor subtype prediction and early detection. Nat Commun. 2019 Dec;10(1):4666.

9. Cohen JD, Li L, Wang Y, Thoburn C, Afsari B, Danilova L, et al. Detection and localization of surgically resectable cancers with a multi-analyte blood test. Science. 2018 Feb 23;359(6378):926–30.

10. Badrick E, Cresswell K, Ellis P, Renehan AG, Crosbie EJ, Crosbie P, et al. Top ten research priorities for detecting cancer early. Lancet Public Health. 2019 Nov;4(11):e551.

11. Collins GS, Reitsma JB, Altman DG, Moons KG. Transparent Reporting of a Multivariable Prediction Model for Individual Prognosis or Diagnosis (TRIPOD) The TRIPOD Statement. Circulation. 2015 Jan 13;131(2):211-9.

12. Wolff RF, Moons KG, Riley RD, Whiting PF, Westwood M, Collins GS, Reitsma JB, Kleijnen J, Mallett S. PROBAST: a tool to assess the risk of bias and applicability of prediction model studies. Annals of internal medicine. 2019 Jan 1;170(1):51-8.

13. CRUK report, Saving Lives, Averting Costs, 2014, https://www.cancerresearchuk.org/sites/default/files/saving_lives_averting_costs.pdf

14. Platt, J Probabilistic outputs for support vector machines and comparisons to regularized likelihood methods. Advances in Large Margin Classifiers. 1999 10 (3): 61–74.

15. XGBoost documentation https://xgboost.readthedocs.io/en/stable/

16. LightGBM documentation https://lightgbm.readthedocs.io/en/latest/

17. Smith L Sansom N Hempihill S Bradley S Shinkins B Wheatstone P Hamilton W Neal R. Trends and variation in urgent referrals for suspected cancer 2009/10 - 2019/20. Accepted at British Journal of General Practice

For peer review only

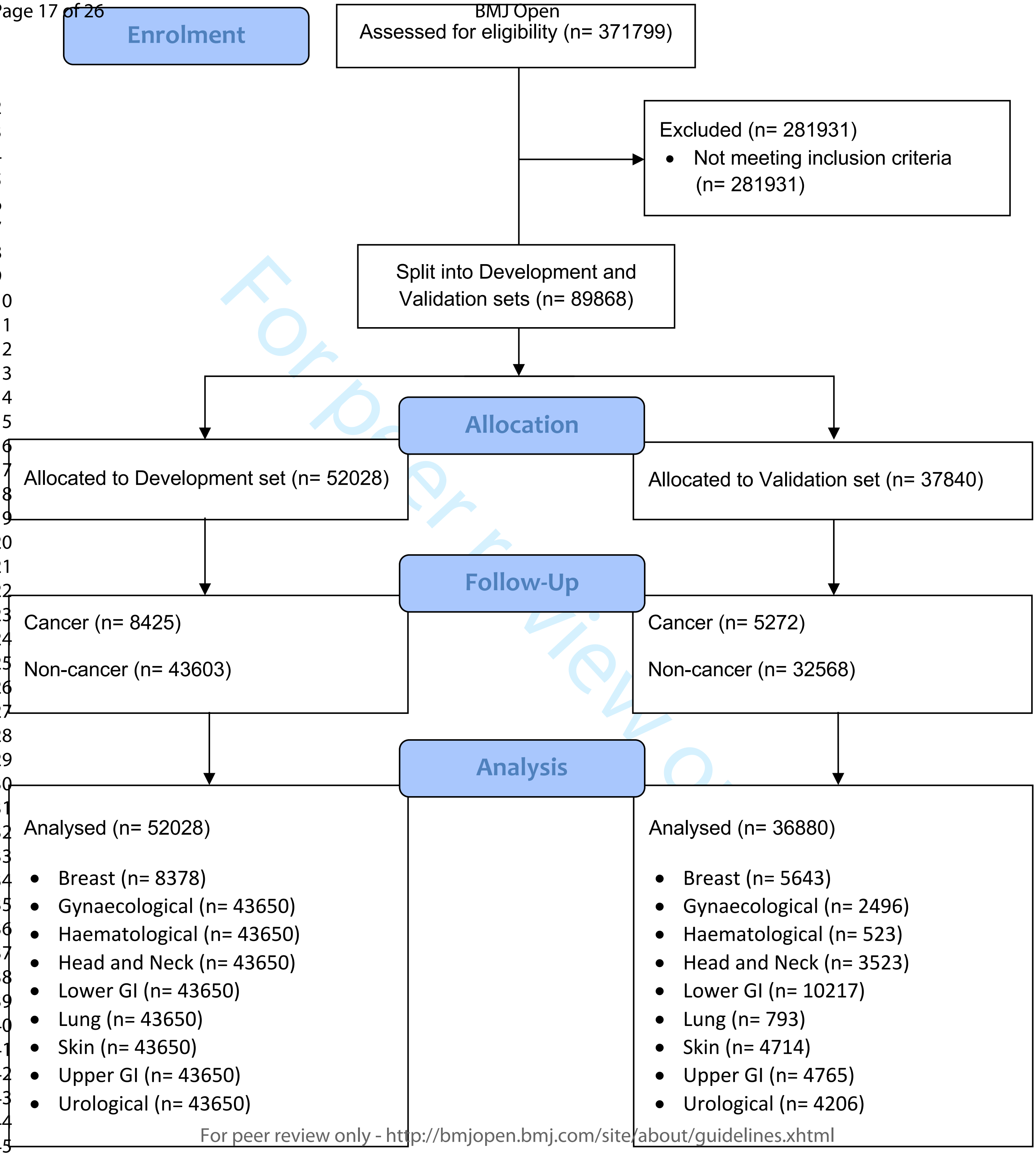

For peer review only - http://bmjopen.bmj.com/site/about/guidelines.xhtml

(Diagram adapted from CONSORT 2010 flow diagram, http://www.consort-statement.org/consort-statement/flow-diagram)

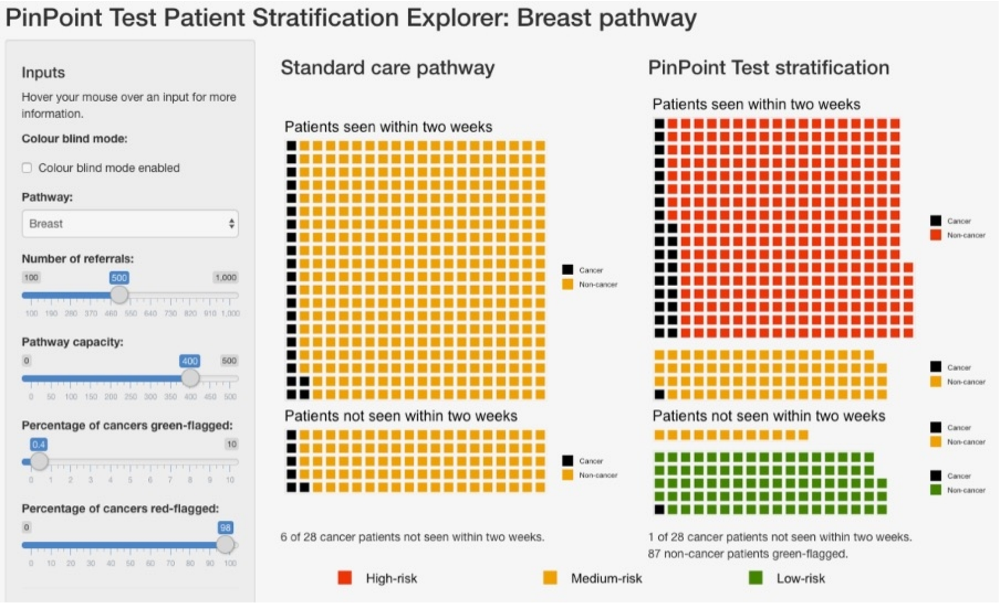

Figure 2: shows stratification of patients on the 2WW breast pathway using the relevant algorithm presented in this work, compared to the standard care pathway. Given an urgent care pathway where the number of referrals exceeds the pathway capacity to see patients within two weeks, use of the test to stratify patients into risk categories (right) leads to a larger proportion of patients with cancer being seen when compared to the standard care pathway (left), in which patients are seen on a first-come, first-served basis. Patients highlighted in red are identified as being at high-risk for cancer (red-flagged), so can be expedited for further diagnostic testing. Patients highlighted in green are identified as being at very low risk for cancer (green-flagged), allowing for initial management in primary care rather than immediate referral to secondary care.

The sliders on the left-hand side show the number of referrals, the number of patients that the pathway can handle in a given time-frame (the pathway capacity), the percentage of cancers which are green-flagged (i.e. setting a very low false negative rate, and therefore high sensitivity c.f. Table 5), and the percentage of cancers that are red-flagged (i.e. identifying cases with high-risk, so that they can be expedited for further diagnostic testing). The red-flagging slider effectively sets a sensitivity for the red-flagging process; setting sensitivity=0.9 corresponds to the results shown in Table 6. The slider for 'percentage of cancers green-flagged' can be used to set the false negative rate and see the resulting performance of the test. Collectively, this represents a possible approach to using the algorithms to improve the triage of patients referred to a 2WW pathway. An interactive version of this is available at https://www.pinpointdatascience.com/patient-test-stratification

We note that for the standard care pathway, all non-cancer patients are labelled in the same colour (yellow) to indicate that they are unstratified by the test.

159x96mm (144 x 144 DPI)

Supplementary Materials

Table of Contents

Test Performance Characteristics **1**
Table S1 .... *1*
Table S2 .... *2*
Clinical Utility Plots **3**
Figure S1 .... *3*
Calibration **4**
Figure S2 .... *4*
Univariate Analyses **5**
Figure S3 .... *6*
ICD-10 Codes **7**
Table S3 .... *7*
Table S4 .... *7*
Reference Costs **7**
Table S5 .... *7*
Prevalence **8**
Table S6 .... *8*

Test Performance Characteristics

In Tables S1 and S2, the "Threshold" column refers to the probability threshold that is applied to the test result for a given pathway in order to get the test performance characteristics given in the corresponding row of the table.

Table S1: Test validation set performance characteristics. Aim: 20% rule-out

Pathway	Threshold	AUC (95% CI)	NPV (95% CI)	TNR (95% CI)	FNR (95% CI)	Sensitivity (95% CI)	Specificity (95% CI)	PPV (95% CI)
Breast	0.0174	0.8007 (0.7750 – 0.8255)	0.9936 (0.9883 – 0.9981)	0.2036 (0.1926 – 0.2143)	0.0224 (0.0067 – 0.0404)	0.9776 (0.9596 – 0.9933)	0.2036 (0.1926 – 0.2143)	0.0672 (0.0601 – 0.0747)
Lower GI	0.0343	0.6798 (0.6566 – 0.7029)	0.9823 (0.9762 – 0.9877)	0.2002 (0.1921 – 0.2081)	0.0652 (0.0457 – 0.0865)	0.9348 (0.9135 – 0.9543)	0.2002 (0.1921 – 0.2081)	0.0609 (0.0559 – 0.0660)
Upper GI	0.0284	0.7323 (0.7008 – 0.7627)	0.9880 (0.9806 – 0.9946)	0.2017 (0.1901 – 0.2137)	0.0420 (0.0196 – 0.0677)	0.9580 (0.9323 – 0.9804)	0.2017 (0.1901 – 0.2137)	0.0653 (0.0576 – 0.0732)
Gynaecological	0.0392	0.8124 (0.7779 – 0.8459)	0.9895 (0.9799 – 0.9979)	0.2040 (0.1871 – 0.2209)	0.0282 (0.0058 – 0.0538)	0.9718 (0.9462 – 0.9942)	0.2040 (0.1871 – 0.2209)	0.0852 (0.0732 – 0.0980)
Urological	0.1062	0.7590 (0.7414 – 0.7757)	0.9525 (0.9358 – 0.9680)	0.2002 (0.1864 – 0.2141)	0.0319 (0.0215 – 0.0432)	0.9681 (0.9568 – 0.9785)	0.2002 (0.1864 – 0.2141)	0.2751 (0.2609 – 0.2900)
Lung	0.0876	0.7376 (0.6938 – 0.7797)	0.9630 (0.9281 – 0.9924)	0.2031 (0.1704 – 0.2331)	0.0327 (0.0067 – 0.0636)	0.9673 (0.9364 – 0.9933)	0.2031 (0.1704 – 0.2331)	0.2249 (0.1934 – 0.2571)
Haematological	0.111	0.7589 (0.7152 – 0.8006)	0.9375 (0.8795 – 0.9868)	0.2095 (0.1694 – 0.2542)	0.0303 (0.0062 – 0.0592)	0.9697 (0.9408 – 0.9938)	0.2095 (0.1694 – 0.2542)	0.3612 (0.3166 – 0.4068)
Head and Neck	0.0423	0.6996 (0.6649 – 0.7334)	0.9748 (0.9623 – 0.9858)	0.2001 (0.1862 – 0.2139)	0.0733 (0.0420 – 0.1083)	0.9267 (0.8917 – 0.9580)	0.2001 (0.1862 – 0.2139)	0.0755 (0.0657 – 0.0852)
Skin	0.0851	0.7220 (0.7057 – 0.7378)	0.9406 (0.9232 – 0.9570)	0.2002 (0.1868 – 0.2130)	0.0391 (0.0283 – 0.0507)	0.9609 (0.9493 – 0.9717)	0.2002 (0.1868 – 0.2130)	0.2796 (0.2656 – 0.2939)

For peer review only

For peer review only - http://bmjopen.bmj.com/site/about/guidelines.xhtml

Table S2: Test validation set performance characteristics. Aim: 90% rule-in

Pathway	Threshold	AUC (95% CI)	NPV (95% CI)	TNR (95% CI)	FNR (95% CI)	Sensitivity (95% CI)	Specificity (95% CI)	PPV (95% CI)
Breast	0.029	0.8007 (0.7746 – 0.8256)	0.9875 (0.9830 – 0.9916)	0.4582 (0.4450 – 0.4715)	0.0990 (0.0678 – 0.1337)	0.9010 (0.8663 – 0.9322)	0.4582 (0.4450 – 0.4715)	0.0890 (0.0793 – 0.0991)
Lower GI	0.041	0.6798 (0.6565 – 0.7029)	0.9799 (0.9745 – 0.9850)	0.2723 (0.2637 – 0.2811)	0.1006 (0.0754 – 0.1262)	0.8994 (0.8738 – 0.9246)	0.2723 (0.2637 – 0.2811)	0.0642 (0.0587 – 0.0697)
Upper GI	0.041	0.7323 (0.7012 – 0.7625)	0.9831 (0.9763 – 0.9893)	0.3363 (0.3227 – 0.3503)	0.0992 (0.0641 – 0.1389)	0.9008 (0.8611 – 0.9359)	0.3363 (0.3227 – 0.3503)	0.0732 (0.0644 – 0.0822)
Gynaecological	0.05	0.8124 (0.7768 – 0.8462)	0.9828 (0.9746 – 0.9900)	0.4674 (0.4473 – 0.4879)	0.1073 (0.0640 – 0.1553)	0.8927 (0.8447 – 0.9360)	0.4674 (0.4473 – 0.4879)	0.1134 (0.0972 – 0.1303)
Urological	0.148	0.7590 (0.7417 – 0.7762)	0.9191 (0.9035 – 0.9336)	0.3548 (0.3379 – 0.3710)	0.0996 (0.0818 – 0.1183)	0.9004 (0.8817 – 0.9182)	0.3548 (0.3379 – 0.3710)	0.3044 (0.2878 – 0.3208)
Lung	0.134	0.7376 (0.6939 – 0.7796)	0.9431 (0.9120 – 0.9702)	0.3625 (0.3238 – 0.3987)	0.0915 (0.0482 – 0.1392)	0.9085 (0.8608 – 0.9518)	0.3625 (0.3238 – 0.3987)	0.2541 (0.2178 – 0.2906)
Haematological	0.189	0.7589 (0.7143 – 0.7999)	0.9118 (0.8633 – 0.9509)	0.4330 (0.3807 – 0.4849)	0.0909 (0.0506 – 0.1412)	0.9091 (0.8588 – 0.9494)	0.4330 (0.3807 – 0.4849)	0.4249 (0.3722 – 0.4759)
Head and Neck	0.047	0.6996 (0.6648 – 0.7339)	0.9751 (0.9644 – 0.9847)	0.2733 (0.2579 – 0.2885)	0.0991 (0.0619 – 0.1393)	0.9009 (0.8607 – 0.9381)	0.2733 (0.2579 – 0.2885)	0.0804 (0.0703 – 0.0911)
Skin	0.141	0.7220 (0.7060 – 0.7380)	0.9236 (0.9100 – 0.9367)	0.3905 (0.3745 – 0.4068)	0.0999 (0.0829 – 0.1175)	0.9001 (0.8825 – 0.9171)	0.3905 (0.3745 – 0.4068)	0.3230 (0.3067 – 0.3392)

For peer review only

Clinical Utility Plots

Figure S1 shows negative predictive value (NPV) against the specificity, i.e. the proportion of patients ruled out, for each pathway. This shows the trade-off for a given pathway between avoiding erroneously ruling out patients who in fact have cancer (high NPV is better) vs the proportion of patients referred who are ruled out of the pathway.

Bootstrap resampling with replacement with 1000 bootstraps was used to generate 95% and 68% confidence intervals on NPV. The solid line marks the median, the dark grey band indicates the 68% confidence interval, and the light grey band indicates the 95% confidence interval.

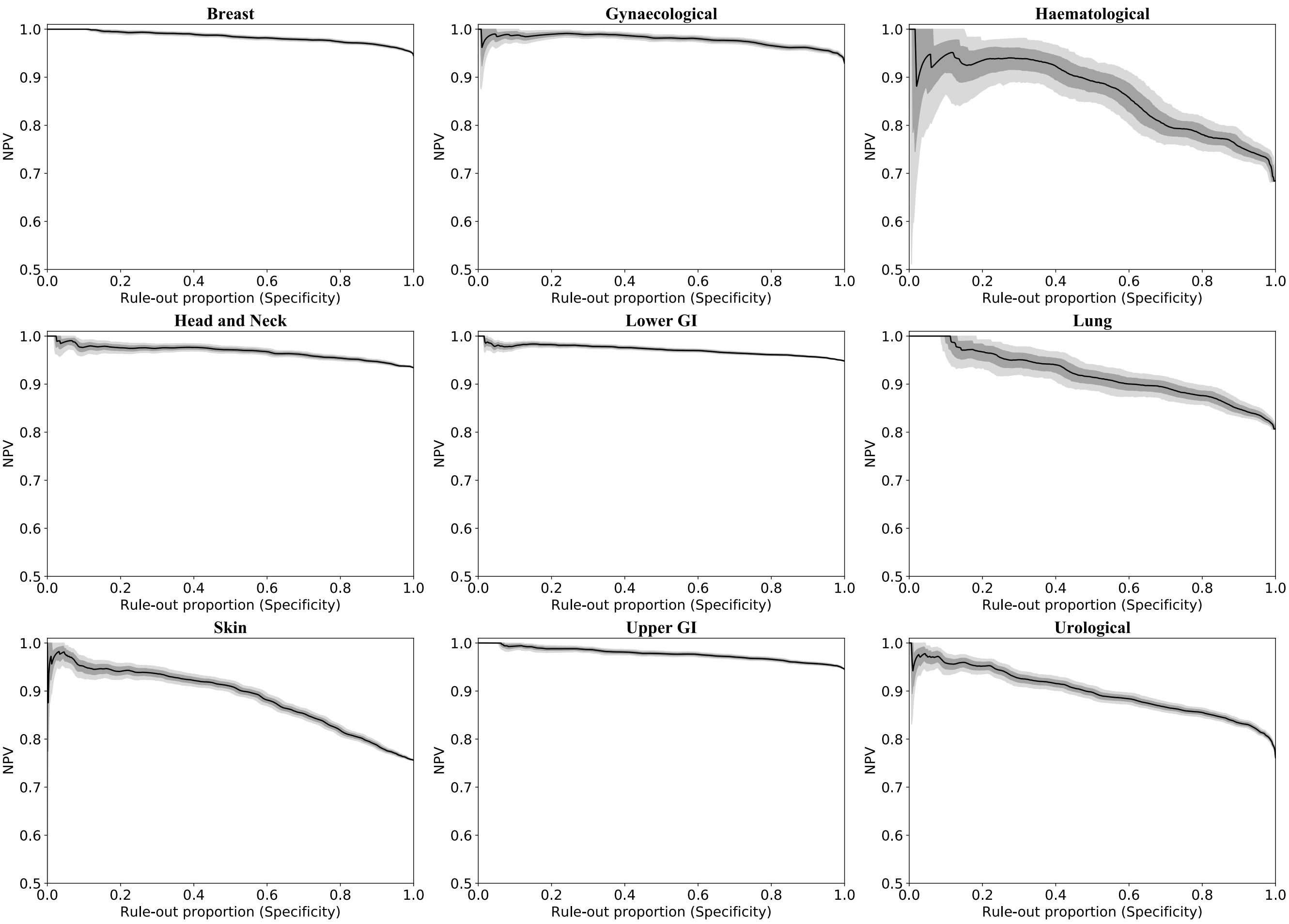

Figure S1: Plots of Negative Predictive Ability against specificity for each pathway. Light and dark grey bands indicate 68% and 95% confidence intervals. See text for details.

For peer review only - http://bmjopen.bmj.com/site/about/guidelines.xhtml

Calibration

Figure S2 shows calibration curves for validation set predictions by the algorithms for each pathway, calculated using equal occupancy bins. Good calibration means that the algorithm results can be interpreted as being the probability of a given patient having cancer and is indicated by the points lying along the dashed diagonal line.

The error bars show the 95% binomial proportion confidence interval, calculated using the Wilson score with continuity correction. The log loss for each pathway is also included.

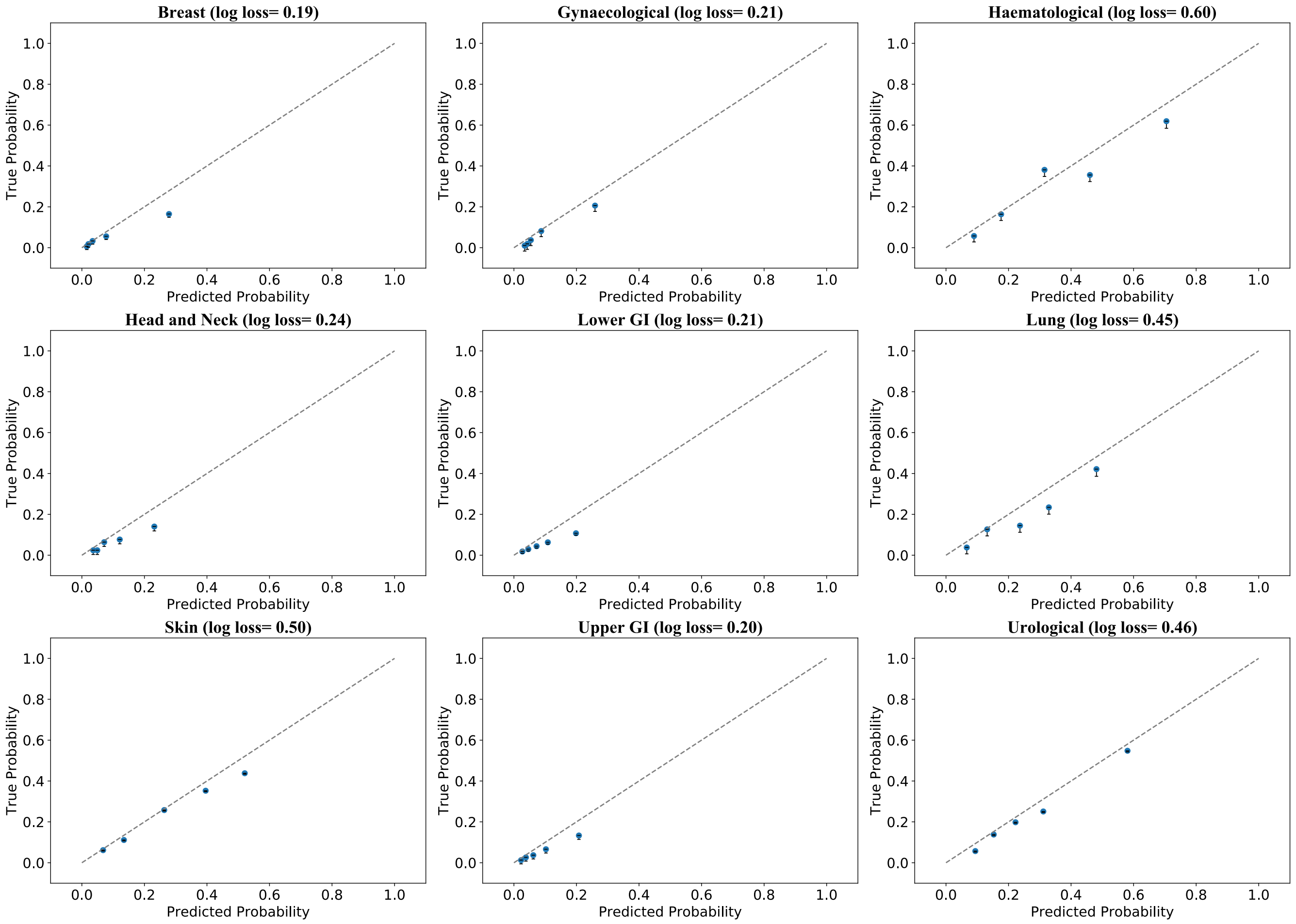

Figure S2: Plots of calibration curves per pathway. Dashed grey line indicates perfect calibration. See text for details.

For peer review only - http://bmjopen.bmj.com/site/about/guidelines.xhtml

Univariate Analyses

Validation set predicted probabilities were generated using the nine algorithms. For each input data feature, ROC AUCs were calculated for cases restricted to those for which the feature data was available, whereby the feature was used as the predictor and the binary cancer flag as the outcome. ROC AUCs were also calculated using the probabilities predicted by the algorithm, with identical restriction of cases applied to allow direct comparison. The difference between the algorithm ROC AUC and the single-feature ROC AUC was then calculated for each feature, ΔAUC.

Using this process, ΔAUCs were calculated for each feature and each pathway-specific algorithm. Bootstrap resampling with replacement with 10000 bootstraps was used to generate 95% confidence intervals on ΔAUC, where both the algorithm ROC AUC and single-feature ROC AUC were calculated on the same bootstrap samples.

Figure S3 shows the median ΔAUCs as black circles with 95% confidence intervals, for each feature and each pathway. Any features with data for less than one hundred patients for a given pathway were removed from the plot for that pathway. Arrows indicate that a confidence interval extends outside the plot area, in the direction of the arrow. The number of cancers and the number of cases were annotated for each feature at the bottom of the plot area. These are in the format "# cancers/# cases". An asterisk was appended to feature names for which the 95% confidence interval does not intersect the line $\Delta AUC = 0$. The feature names are assigned according to the category into which the blood test falls—"FBC" for blood counts, "Bio" for biochemistry, and "TM" for tumour markers—with numbers assigned arbitrarily but consistently across the subplots.

For peer review only - http://bmjopen.bmj.com/site/about/guidelines.xhtml

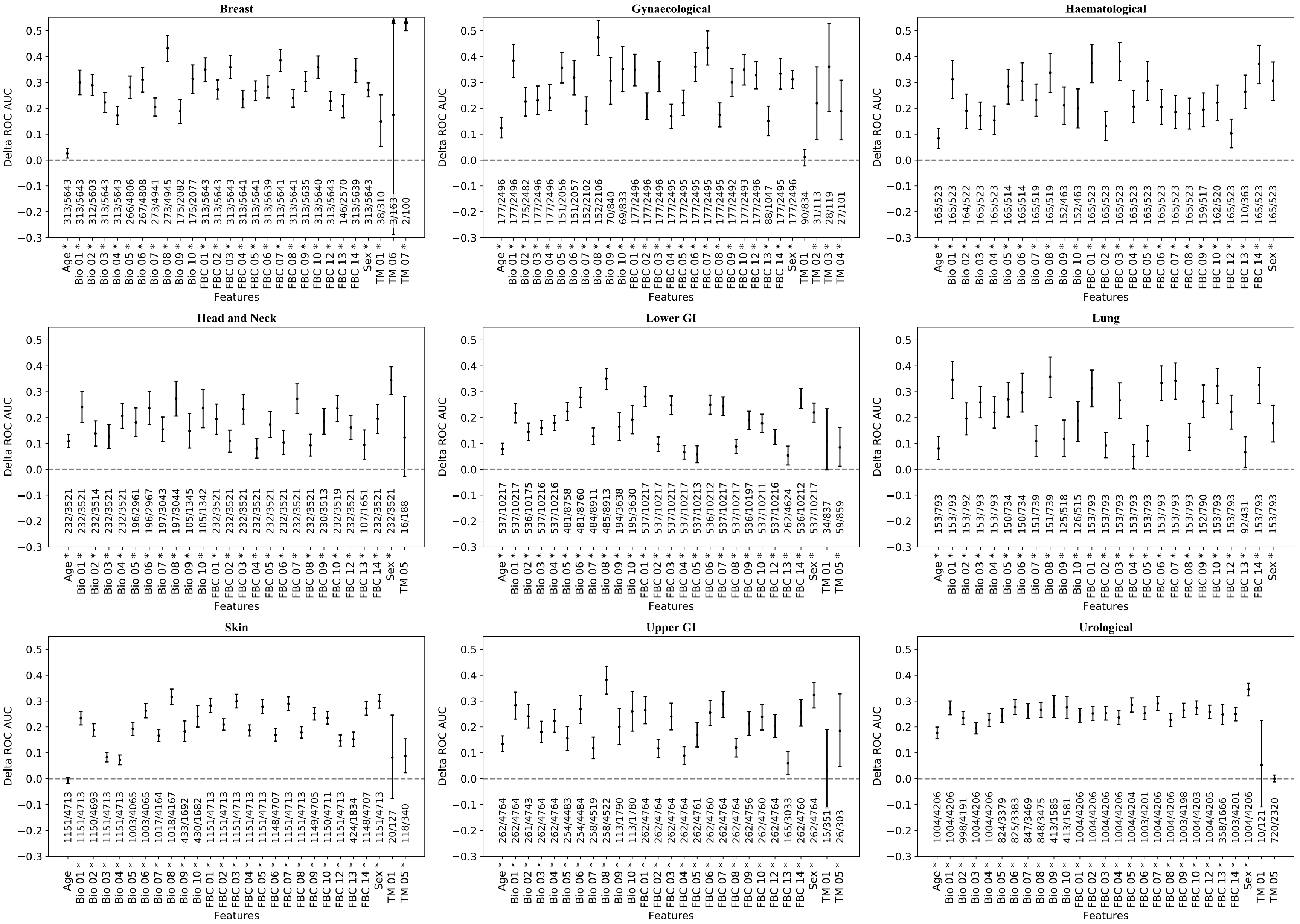

Figure S3: Plots of ΔAUC per feature per pathway. The vertical confidence intervals show the difference between ROC AUC performance for the algorithm and those that one obtains from using an individual analyte. See text for details.

ICD-10 Codes

Table S3: ICD-10 codes designated as "cancer" for the algorithms

ICD-10 code	ICD-10 text
C00-C14	Malignant neoplasms of lip, oral cavity and pharynx
C15-C26	Malignant neoplasms of digestive organs
C30-C39	Malignant neoplasms of respiratory and intrathoracic organs
C40-C41	Malignant neoplasms of bone and articular cartilage
C43-C44	Melanoma and other malignant neoplasms of skin
C45-C49	Malignant neoplasms of mesothelial and soft tissue
C50-C50	Malignant neoplasm of breast
C51-C58	Malignant neoplasms of female genital organs
C60-C63	Malignant neoplasms of male genital organs
C64-C68	Malignant neoplasms of urinary tract
C69-C72	Malignant neoplasms of eye, brain and other parts of central nervous system
C73-C75	Malignant neoplasms of thyroid and other endocrine glands
D00	Carcinoma in situ of oral cavity, oesophagus and stomach
D01	Carcinoma in situ of other and unspecified digestive organs
D02	Carcinoma in situ of middle ear and respiratory system
D03	Melanoma in situ
D04	Carcinoma in situ of skin
D05	Carcinoma in situ of breast
D07	Carcinoma in situ of other and unspecified genital organs
D09	Carcinoma in situ of other and unspecified sites

Table S4: ICD-10 codes designated as "benign" for the algorithms

ICD-10 code	ICD-10 text
D06	Carcinoma in situ of cervix uteri
D10-D36	Benign neoplasms
D37-D48	Neoplasms of uncertain or unknown behaviour

Reference Costs

Table S5 shows the reference costs for the analytes that are used as inputs to the algorithms. These costs, from the 2018-2019 reference schedule, were also used for health economics that have been performed and will be published separately.

Table S5: NHS reference costs, 2018-2019

Item	Category	Cost (2018-19 Ref Schedule)
Full Blood Counts	Haematology	£3.00
Urea & Electrolytes	Clinical Biochemistry	£1.00
CA125	Clinical Biochemistry	£1.00
CA19-9	Clinical Biochemistry	£1.00
Carcinoembryonic Antigen	Clinical Biochemistry	£1.00
CA15-3	Clinical Biochemistry	£1.00
PSA	Clinical Biochemistry	£1.00
Alpha Fetoprotein	Clinical Biochemistry	£1.00
Human Chorionic Gonadotrophin	Clinical Biochemistry	£1.00
C-Reactive Protein	Clinical Biochemistry	£1.00
Liver Function Tests	Clinical Biochemistry	£1.00
Phlebotomy	-	£4.00
**Total NHS Costs**	-	**£17.00**

For peer review only - http://bmjopen.bmj.com/site/about/guidelines.xhtml

Prevalence
Table S6 shows the prevalences, by pathway, for the whole cohort of patients 2011-19, including those excluded from the analyses. A comparison with Table 2 shows differences between the overall prevalences and those for the included patients, highlighting possible sources of spectrum bias. Typical prevalences for the 2WW pathways in NHSE are given for 2009-10 and 2019-20 in Smith et al. [main paper reference 17]. The right hand most column corresponds to the cancer outcomes used in the analyses in this paper, and we note that these are typically somewhat higher than 2WW prevalence rates due to the inclusion of any cancer diagnosis up to 12 months after the referral date. To illustrate this, the middle column shows the cancer prevalence when the diagnoses of the cohort of patients are restricted to only those found via the 2WW pathways, and within 62 days of referral.

Table S6: Cancer prevalence for whole cohort of patients 2011-19, including those excluded from the analyses, for two examples of diagnosis inclusion criterion. See text for details.

Pathway	Cancer prevalence (%) Restricted diagnoses (see text)	Cancer prevalence (%) All diagnoses (see text)
Breast	6.8	8.0
Lower GI	7.1	11.5
Upper GI	10.6	15.4
Gynaecological	11.3	14.3
Urological	25.0	30.6
Lung	30.0	40.4
Haematological	33.1	38.3
Head and Neck	8.8	12.6
Skin	19.4	22.3

For peer review only

For peer review only - http://bmjopen.bmj.com/site/about/guidelines.xhtml

TRIPOD Checklist: Prediction Model Development and Validation

Section/Topic	Item		Checklist Item	Page
**Title and abstract**				
Title	1	D;V	Identify the study as developing and/or validating a multivariable prediction model, the target population, and the outcome to be predicted.	1
Abstract	2	D;V	Provide a summary of objectives, study design, setting, participants, sample size, predictors, outcome, statistical analysis, results, and conclusions.	1
**Introduction**				
Background and objectives	3a	D;V	Explain the medical context (including whether diagnostic or prognostic) and rationale for developing or validating the multivariable prediction model, including references to existing models.	2
	3b	D;V	Specify the objectives, including whether the study describes the development or validation of the model or both.	2
**Methods**				
Source of data	4a	D;V	Describe the study design or source of data (e.g., randomized trial, cohort, or registry data), separately for the development and validation data sets, if applicable.	3
	4b	D;V	Specify the key study dates, including start of accrual; end of accrual; and, if applicable, end of follow-up.	3
Participants	5a	D;V	Specify key elements of the study setting (e.g., primary care, secondary care, general population) including number and location of centres.	3
	5b	D;V	Describe eligibility criteria for participants.	3
	5c	D;V	Give details of treatments received, if relevant.	NA
Outcome	6a	D;V	Clearly define the outcome that is predicted by the prediction model, including how and when assessed.	3
	6b	D;V	Report any actions to blind assessment of the outcome to be predicted.	3
Predictors	7a	D;V	Clearly define all predictors used in developing or validating the multivariable prediction model, including how and when they were measured.	3
	7b	D;V	Report any actions to blind assessment of predictors for the outcome and other predictors.	3
Sample size	8	D;V	Explain how the study size was arrived at.	4
Missing data	9	D;V	Describe how missing data were handled (e.g., complete-case analysis, single imputation, multiple imputation) with details of any imputation method.	4
Statistical analysis methods	10a	D	Describe how predictors were handled in the analyses.	4
	10b	D	Specify type of model, all model-building procedures (including any predictor selection), and method for internal validation.	4
	10c	V	For validation, describe how the predictions were calculated.	4
	10d	D;V	Specify all measures used to assess model performance and, if relevant, to compare multiple models.	4
	10e	V	Describe any model updating (e.g., recalibration) arising from the validation, if done.	4
Risk groups	11	D;V	Provide details on how risk groups were created, if done.	NA
Development vs. validation	12	V	For validation, identify any differences from the development data in setting, eligibility criteria, outcome, and predictors.	4
**Results**				
Participants	13a	D;V	Describe the flow of participants through the study, including the number of participants with and without the outcome and, if applicable, a summary of the follow-up time. A diagram may be helpful.	5
	13b	D;V	Describe the characteristics of the participants (basic demographics, clinical features, available predictors), including the number of participants with missing data for predictors and outcome.	4
	13c	V	For validation, show a comparison with the development data of the distribution of important variables (demographics, predictors and outcome).	6/7
Model development	14a	D	Specify the number of participants and outcome events in each analysis.	6/7
	14b	D	If done, report the unadjusted association between each candidate predictor and outcome.	supp
Model specification	15a	D	Present the full prediction model to allow predictions for individuals (i.e., all regression coefficients, and model intercept or baseline survival at a given time point).	NA
	15b	D	Explain how to the use the prediction model.	NA
Model performance	16	D;V	Report performance measures (with CIs) for the prediction model.	8/9
Model-updating	17	V	If done, report the results from any model updating (i.e., model specification, model performance).	NA
**Discussion**				
Limitations	18	D;V	Discuss any limitations of the study (such as nonrepresentative sample, few events per predictor, missing data).	12
Interpretation	19a	V	For validation, discuss the results with reference to performance in the development data, and any other validation data.	NA
	19b	D;V	Give an overall interpretation of the results, considering objectives, limitations, results from similar studies, and other relevant evidence.	11/12
Implications	20	D;V	Discuss the potential clinical use of the model and implications for future research.	11/12
**Other information**				
Supplementary information	21	D;V	Provide information about the availability of supplementary resources, such as study protocol, Web calculator, and data sets.	supp
Funding	22	D;V	Give the source of funding and the role of the funders for the present study.	4

*Items relevant only to the development of a prediction model are denoted by D, items relating solely to a validation of a prediction model are denoted by V, and items relating to both are denoted D;V. We recommend using the TRIPOD Checklist in conjunction with the TRIPOD Explanation and Elaboration document.

BMJ Open

Development and validation of multivariable machine learning algorithms to predict risk of cancer in symptomatic patients referred urgently from primary care

Journal:	*BMJ Open*
Manuscript ID	bmjopen-2021-053590.R2
Article Type:	Original research
Date Submitted by the Author:	09-Feb-2022
Complete List of Authors:	Savage, Richard; PinPoint Data Science Ltd, Nexus, Discovery Way<br>Messenger, Mike; University of Leeds<br>Neal, Richard; University of Leeds; University of Exeter<br>Ferguson, Rosie; PinPoint Data Science Ltd<br>Johnston, Colin; University of Leeds<br>Lloyd, Katherine L; PinPoint Data Science Ltd<br>Neal, Matthew D; PinPoint Data Science Ltd<br>Sansom, Nigel; PinPoint Data Science Ltd<br>Selby, Peter; University of Leeds<br>Sharma, Nisha; Leeds Teaching Hospitals NHS Trust<br>Shinkins, Bethany; University of Leeds<br>Skinner, Jim R; PinPoint Data Science Ltd<br>Tully, Giles; PinPoint Data Science Ltd<br>Duffy, Sean; Leeds Teaching Hospitals NHS Trust<br>Hall, Geoff; University of Leeds
**Primary Subject Heading**:	Oncology
Secondary Subject Heading:	Diagnostics, Health informatics
Keywords:	Health informatics < BIOTECHNOLOGY & BIOINFORMATICS, ONCOLOGY, STATISTICS & RESEARCH METHODS

SCHOLARONE™
Manuscripts

I, the Submitting Author has the right to grant and does grant on behalf of all authors of the Work (as defined in the below author licence), an exclusive licence and/or a non-exclusive licence for contributions from authors who are: i) UK Crown employees; ii) where BMJ has agreed a CC-BY licence shall apply, and/or iii) in accordance with the terms applicable for US Federal Government officers or employees acting as part of their official duties; on a worldwide, perpetual, irrevocable, royalty-free basis to BMJ Publishing Group Ltd ("BMJ") its licensees and where the relevant Journal is co-owned by BMJ to the co-owners of the Journal, to publish the Work in this journal and any other BMJ products and to exploit all rights, as set out in our licence.

The Submitting Author accepts and understands that any supply made under these terms is made by BMJ to the Submitting Author unless you are acting as an employee on behalf of your employer or a postgraduate student of an affiliated institution which is paying any applicable article publishing charge ("APC") for Open Access articles. Where the Submitting Author wishes to make the Work available on an Open Access basis (and intends to pay the relevant APC), the terms of reuse of such Open Access shall be governed by a Creative Commons licence – details of these licences and which Creative Commons licence will apply to this Work are set out in our licence referred to above.

Other than as permitted in any relevant BMJ Author's Self Archiving Policies, I confirm this Work has not been accepted for publication elsewhere, is not being considered for publication elsewhere and does not duplicate material already published. I confirm all authors consent to publication of this Work and authorise the granting of this licence.

For peer review only

Development and validation of multivariable machine learning algorithms to predict risk of cancer in symptomatic patients referred urgently from primary care

Richard S Savage[1* +], Mike Messenger[2,5*], Richard D Neal[2,5, 6*], Rosie Ferguson[1], Colin Johnston[3], Katherine L Lloyd[1], Matthew D Neal[1], Nigel Sansom[1], Peter Selby[2,4,5], Nisha Sharma[3], Bethany Shinkins[2,5], Jim R Skinner[1], Giles Tully[1], Sean Duffy[3**], Geoff Hall[2,3,5]**

(1) PinPoint Data Science Ltd, (2) University of Leeds, (3) Leeds Teaching Hospitals Trust, (4) Chair of the PinPoint Scientific Advisory Board, (5) NIHR MedTech and In Vitro Diagnostic Co-Operative Leeds (6) University of Exeter
* Joint lead author (ORCID ID: 0000-0001-6025-1571), ** Joint last author

+ Corresponding author (Richard S Savage, rich.savage@pinpointdatascience.com)

For peer review only

Abstract

Objectives: To develop and validate tests to assess the risk of any cancer for patients referred to the NHS Urgent Suspected Cancer (Two Week Wait, 2WW) clinical pathways.

Setting: Primary and secondary care, one participating regional centre.

Participants: Retrospective analysis of data from 371,799 consecutive 2WW referrals in the Leeds region from 2011-2019. The development cohort was composed of 224,669 consecutive patients with an urgent suspected cancer referral in Leeds between January 2011 and December 2016. The diagnostic algorithms developed were then externally validated on a similar consecutive sample of 147,130 patients (between January 2017 and December 2019). All such patients over the age of 18 with a minimum set of blood counts and biochemistry measurements available were included in the cohort.

Primary and secondary outcome measures: sensitivity, specificity, NPV, PPV, ROC curve AUC, calibration curves

Results: We present results for two clinical use-cases. In use-case 1, the algorithms identify 20% of patients who do not have cancer and may not need an urgent 2WW referral. In use-case 2, they identify 90% of cancer cases with a high probability of cancer that could be prioritised for review.

Conclusions: Combining a panel of widely available blood markers produces effective blood tests for cancer for NHS 2WW patients. The tests are affordable, and can be deployed rapidly to any NHS pathology laboratory with no additional hardware requirements.

Strengths and Limitations of this Study

The principal strengths of this work are:

- It is based on well-validated, low-cost clinical assays already available at scale in NHS pathology laboratories; the tests could therefore be deployed across the UK very rapidly, with no additional hardware requirements.
- The large numbers of cases reported, and that the performance estimates are conservative due to missing data and the historical nature of the blood measurements; prospective evaluation will not suffer from these drawbacks.

The principal limitations of this work are:

- That the development and validation was done only in one centre.
- There is a possible source of bias, in that the subset of patients who had retrospective blood data may not be representative of the overall 2WW cohort.
- We have only reported the validation on a retrospective sample; a prospective evaluation is needed.

For peer review only

1 Background

A major NHS cancer policy to diagnose cancer earlier led to the introduction of Urgent Suspected Cancer referrals. These referrals are predicated on the risk of symptomatic patients having cancer.[1] Trusts assess patients within two weeks (‘two-week wait’ (2WW) referral). The 2WW pathways have contributed to improving outcomes; higher general practice use of referrals for suspected cancer is associated with lower mortality for the four most common types of cancer (prostate, breast, lung, and colorectal).[2]

This approach places a major strain on diagnostic services on NHS England, with over 2 million 2WW referrals annually, and a 10% year-on-year increase in referrals over the past decade.[3] This highlights an unsustainable burden on existing services, workforce and financial resources. Whilst there is variation between cancer pathways, only 7% overall of 2WW referral patients are diagnosed with cancer.[3] Many patients are therefore subject to unnecessary psychological distress, as well as being exposed to diagnostic tests which may inadvertently cause harm. Clearly there is a need to improve the efficiency of these pathways.

These challenges are exacerbated by the current COVID-19 crisis. The NHS capacity to assess 2WW referrals is reduced, and a backlog of referrals continues to build.[3,4] These unprecedented challenges urgently require new solutions. COVID-19 has presented an opportunity for GPs to permanently change how they use emerging technologies.[5]

Many biomarkers have been evaluated for their use in cancer diagnosis; however only a few are currently used in either primary or secondary care settings. A systematic mapping review identified 94 ctDNA studies alone, highlighting how much more work is required prior to clinical use.[6] Companies like GRAIL and Freenome are pursuing this, with clinical trials ongoing.[7,8] There is also evidence that signals from a range of different analytes can be usefully combined via machine learning.[9]

Using such approaches to triage cancer referrals should bring benefits to patients, health-systems and the economy. For example, a *rule-out* test for symptomatic patients, like those referred to the NHS 2WW, could identify those with very low cancer risk, allowing many patients without cancer to avoid unnecessary procedures and freeing up diagnostic capacity for those at greater risk.

The work presented in this paper addresses the top three priority areas identified by Badrick et al (2019), including: a simple, non-invasive, painless and convenient test to detect cancer early; a blood test to detect some or all cancers early that can be included into routine care; and a test that is easily accessible to General Practice.[10]

We report the development and validation of a set of machine learning algorithms to provide a calibrated risk probability of cancer (a score between zero and one, higher values indicating greater risk of cancer) for triaging symptomatic patients. A calibrated risk probability has a variety of clinical uses. This paper focuses on the two use-cases for the NHS 2WW:

Use-Case 1 - a rule-out test when patient has a very low risk of cancer, allowing initial management in primary care.

Use-Case 2 - a way of identifying patients at high risk of having cancer to fast-track them for further tests.

For peer review only

2 Methods

Methodological Design and Source of Data

This work is a single centre, retrospective diagnostic prediction study (classified as a Type 2b study by the TRIPOD statement.[11] The prediction algorithms were developed and validated on a large data set from a single geographic area, split chronologically into two independent cohorts.

The data set contained 371,799 consecutive 2WW referrals in the Leeds region from 2011-2019. The development cohort was composed of 224,669 consecutive patients with an urgent suspected cancer referral in Leeds between January 2011 and December 2016. The diagnostic algorithms developed were then externally validated on a similar consecutive sample of 147,130 patients (between January 2017 and December 2019). Both development and validation sets were selected using the same inclusion and exclusion criteria and both received the same pre-processing, consisting of removing greater-than (">") symbols from blood analyte values in the data, and setting data values with less-than ("<") values to zero. This is a simple imputation for the case where a pathology laboratory returns a result outside the reportable range. Because the chosen machine learning algorithms are not sensitive to scaling of individual variables, it was not necessary to normalise the inputs.

2.1 Participants

Patients were selected because they received a 2WW referral to Leeds Teaching Hospitals NHS Trust during the above timeframe. Referrals were included for all 2WW pathways, and all patients over the age of 18 with a minimum set of blood counts and biochemistry measurements available were included in the cohort. Occasional multiple referrals of the same patient (for example to different 2WW pathways) is expected in this data set – such instances are infrequent, and are not modelled any differently from other referrals. While information about repeated referral could, in principle, aid the algorithm, this would make the algorithm much harder to deploy in practice as it would need reliable access to an electronic healthcare record, rather than just being linked directly to the Laboratory Information Management System (LIMS) which handles the pathology lab data flows. We have therefore avoided this on practical grounds, for the time being.

Patients from all 2WW pathways were included in the development set; patients from the nine 2WW pathways at LTHT considered in this paper were included in the validation set. The reason for including all cases in the development set is that our goal was to train algorithms that could assist with pan-cancer diagnosis, including cancer cases which have not been referred down the correct pathway. Validation was restricted to these nine 2WW pathways (which account for ~98% of all 2WW referrals in England) because the remaining pathways, being much smaller, did not have sufficient validation data to provide useful validation. Patients not fulfilling these criteria were excluded from the analysis. All patients were followed up to 12 months after the conclusion of their referral, or until February 2020. Patients in the validation set (i.e. referred from January 2017 onwards) only required the outcome of the 2WW referral and therefore the possibility of censoring of outcomes up to 12 months did not affect the validation results.

2.2 Outcome

The algorithms were trained to predict whether or not a patient would receive a cancer diagnosis. Outcome labels were derived from ICD10 diagnostic codes from the Leeds secondary care cancer clinical database. 'Cancer' was defined as any patient diagnosed with a malignant (ICD10 'C' codes) or in situ (appropriate subset of ICD10 'D' codes) neoplasm as the result of their referral or within the subsequent 12-month period for the purposes of model development. Diagnoses as the result of an urgent referral were used as outcomes in the validation analyses, to match the intended

For peer review only

clinical setting. Benign neoplasms were defined as 'Not Cancer'. The full list of ICD10 codes designated as 'cancer' are in the supplementary materials.

2.3 Predictors

The variables for each patient include a full blood count, a range of biochemistry measurements, a panel of standard tumour markers, plus age and sex. All predictors were included on their natural scale (i.e. they were not normalised or dichotomised).

As a retrospective cohort, blood measurements were used where they were available in the database up to 90 days prior to referral or up to 14 days post referral. This was done to seek a reasonable balance between missing data and possible bias (for example if blood measurements were made after a diagnosis had been established). For example, it is risky to use blood measurements taken more than 14 days post-referral as there is an increasing chance that those bloods could have been ordered by a clinician in response to a confirmed diagnosis of cancer. In routine clinical use, all model predictors would be available at the time.

2.4 Sample Size

The protocol for this work stated a goal of achieving a Negative Predictive Value (NPV) of 0.99 or greater for the rule-out use-case. Because NPVs below 0.99 are undesirable, we consider sample sizes as they impact the lower half of the 95% CI for NPV. For a 0.05 lower CI size, we require 100 total patients being ruled-out; for a 0.02 lower CI size we require 300 patients. With a design goal of achieving a 20% rule-out rate, this would therefore require approximately (100)/(0.2) = 500 total cases per pathway for a 0.05 lower CI size, or (300)/(0.2) = 1500 total cases per pathway for a 0.02 lower CI size.

The validation set meets the above sample size criteria for 7 of the 9 2WW pathways for which results are presented. The other two pathways (lung and haematological) are high prevalence pathways (see Table 1, 2), and so it was decided to also include results for these two pathways as the 95% CI are provided for all results to make clear the level of uncertainty present due to sample sizes. The remaining (smaller) 2WW pathways as recorded in the clinical data were also considered (Testicular, Brain/CNS, Sarcomas, Children's Cancer, Acute Leukaemia, other cancer), but we did not develop algorithms for these as the available sample sizes were judged too small to train and validate effective models.

2.5 Management of Missing Data

Missing data is a key issue for this cohort as many patients did not have bloods in this timeframe (see Tables 1, 2). Patients were identified who had full blood counts and a minimum subset of biochemistry data, and this subset was used to train the algorithms. The core algorithms use a gradient boosting model including an inbuilt method for imputing missing data which infers from the data how to handle missing data values, by learning at each decision tree node in the ensemble which branch a missing value should be assigned to. Early work during model development showed that this inbuilt method modestly outperformed (in a statistical sense) simple imputation methods, and has the advantage of simplifying the model development somewhat.

2.6 Patient and Public Involvement

Multiple public and patient consultations have been undertaken in relation to this work, initially via the NIHR-Leeds In Vitro Diagnostics Co-Operative (Leeds MIC) Public and Patient Interaction/Engagement group, expanding to Healthwatch Leeds and Healthwatch Kirklees as well as the West Yorkshire and Harrogate Cancer Alliance and CANTEST programme patient panels. Several sessions have been held and feedback gained on the clinical use of the tests presented in this work.

For peer review only

2.7 Statistical Analysis Methods

The goal of the algorithms is to produce a well-calibrated prediction of the probability that a patient has cancer. The type of model required is a probabilistic classifier—a model that predicts the probabilities of a given patient belonging to one of several distinct classes.

The development set was used to identify appropriate models and calibration methods and to tune the hyperparameters for those models. Methods and hyperparameters were compared and tuned using 5-fold cross-validation. This was concluded and results locked down before validation.

The model structure selected using the development set is a combination of a core machine learning algorithm with good predictive performance(gradient boosting), plus a calibration step (polynomial logistic regression, a modified version of Platt Scaling [12]). Gradient boosting was chosen for a number of pragmatic and statistical performance reasons. It is generally seen to perform very well in comparison to other methods on structured data sets such as are used in this paper and we observed the same thing during early development work. Gradient Boosting using decision trees is also able to straightforwardly handle input variables with wildly different distributions (e.g. tumour markers vs blood counts). There are several very good Python packages available that implement gradient boosting (we use XGBoost [13] and LightGBM [14]), and these packages have built-in methods for handling missing data. Gradient boosting also has a modest computational load for both training and prediction. Platt Scaling is a standard calibration method which uses logistic regression. We have modified this to use polynomial logistic regression because we found this gave better calibration performance with the outputs of our gradient boosting algorithms.

The outcome classes for this work are significantly imbalanced, with substantially fewer cancers than non-cancers (see prevalences in Table 2). The imbalanced classes are accounted for via upweighting the importance of the cancer patients in the gradient boosting algorithms. The same weight is applied to all cancer patients, and this is tuned as a hyperparameter during the development work (i.e. using cross-validation on the development set).

Prior to any analysis variables were selected based on: cost and relevance, availability in NHS pathology labs and prior knowledge from medical literature that they might reasonably be expected to contain some cancer-relevant information. Variable selection in the statistical sense (i.e. using the development data set) was not carried out and the gradient boosting algorithm used in this work is able to down-weight any input variables which are of lesser statistical importance (in terms of contribution to making good predictions).

The validation set was used to validate the locked-down algorithms. After this no changes were made to the algorithms, results are presented below.

3 Results

Figure 1 shows a CONSORT flow diagram for this work.

Tables 1 and 2 show the total number of cases per pathway, and the number of those cases meeting the inclusion criteria. Tables 3 and 4 show the age and sex demographics of the included patients, by pathway and by development/validation set.

Table 5 shows test performance characteristics for nine urgent referral pathways for use-case 1 (rule-out). The goal here is to successfully identify 20% of non-cancer patients (a specificity of 0.2)

For peer review only - http://bmjopen.bmj.com/site/about/guidelines.xhtml

who are at very low risk of cancer, so that other possible causes of their symptoms can be considered rather than continuing with a 2WW referral.

Table 6 shows test performance characteristics for use-case 2 (triage), to identify patients at higher risk of cancer who would be considered for priority through the urgent referral pathway. The goal here is to successfully red-flag 90% of cancer cases (a sensitivity of 0.9) for priority investigation.

Figure 2 shows an example of stratification via a test, compared with the existing standard care pathway. In this example, 500 patients present to the breast pathway, which is overloaded and only able to see 400 of these patients within two weeks of their referral. The standard care pathway is modelled as first-come first-served, and so the proportion of patients with cancer is the same in the patients seen and the patients not seen. Using the test for stratification, the patients are stratified into high, medium and low-risk groups. Patients are then seen in risk order - in this example, all of the high-risk patients are seen, and some of the medium-risk patients are seen. Under stratification, far more of the patients with cancer are seen, and of the patients not seen, a far smaller proportion have cancer. An interactive version of this is available at https://www.pinpointdatascience.com/patient-test-stratification

For peer review only

Table 1: Total Number of Cases per Pathway (2011-2019)

Pathway	2011-2016	2017-2019	Total
Breast	60673	36561	97234
Lower GI	31966	22331	54297
Upper GI	18986	11938	30924
Gynaecological	16533	11599	28132
Urological	20209	13326	33535
Lung	7607	3237	10844
Haematological	2273	1323	3596
Head and Neck	22594	14558	37152
Skin	38605	29239	67844
**Key Pathways Total**	**219446**	**144112**	**363558**
**All Pathways Total**	**224669**	**147130**	**371799**

Table 2: Number of Cases Meeting Bloods Criteria

**Pathway**	**Development Set**			**Validation Set**		
	**# Cancer**	**# Non-cancer**	**Prevalence**	**# Cancer**	**# Non-cancer**	**Prevalence**

Breast	807	7571	9.6	424	5219	7.5
Lower GI	1257	11401	9.9	856	9361	8.4
Upper GI	662	5317	11.1	428	4337	9.0
Gynaecological	407	3098	11.6	218	2278	8.7
Urological	1836	4677	28.2	1143	3063	27.2
Lung	687	1380	33.2	177	616	22.3
Haematological	403	654	38.1	180	343	34.4
Head and Neck	546	4293	11.3	346	3177	9.8
Skin	1468	3910	27.3	1287	3427	27.3

Table 2: Details of the cases which meet the acceptance criteria for the analyses presented in this paper. Prevalence is calculated only for those cases meeting the criteria, and not for all patients entering a given pathway.

For peer review only

Table 3: Age Demographics

**Pathway**	**Development Set**			**Validation Set**		
	**Age 25th percentile**	**Age median**	**Age 75th percentile**	**Age 25th percentile**	**Age median**	**Age 75th percentile**
Breast	36	48	64	35	48	62
Lower GI	59	69	78	59	69	78
Upper GI	57	68	77	55	67	76
Gynaecological	49	57	69	46	54	66
Urological	58	68	77	59	69	78
Lung	58	69	78	57	67	76
Haematological	43	63	76	43	62	75.5
Head and Neck	47	60	72	47	59	72
Skin	52	69	80	52	69	80

Table 4: Sex Demographics

**Pathway**	**Development Set**		**Validation Set**	
	**# Female (%)**	**# Male (%)**	**# Female (%)**	**# Male (%)**

Breast	7345 (87.67)	1033 (12.33)	5146 (91.19)	497 (8.82)
Lower GI	6889 (54.42)	5769 (45.58)	5529 (54.12)	4688 (45.88)
Upper GI	3346 (55.96)	2633 (44.04)	2746 (57.63)	2019 (42.37)
Gynaecological	3505 (100.00)	0 (0.00)	2495 (99.96)	1 (0.04)
Urological	1700 (26.10)	4813 (73.90)	904 (21.49)	3302 (78.51)
Lung	947 (45.82)	1120 (54.19)	363 (45.78)	430 (54.22)
Haematological	506 (47.87)	551 (52.13)	227 (43.40)	296 (56.60)
Head and Neck	2755 (56.93)	2084 (43.07)	2080 (59.04)	1443 (40.96)
Skin	2924 (54.37)	2454 (45.63)	2614 (55.45)	2100 (44.55)

Table 5: 20% Rule-out

Pathway	Proportion of non-cancers ruled-out (specificity) (95% CI)	Negative Predictive Value (95% CI)	Sensitivity (95% CI)
Breast	0.2036 (0.1926–0.2143)	0.9936 (0.9883–0.9981)	0.9776 (0.9596 - 0.9933)
Lower GI	0.2002 (0.1921–0.2081)	0.9823 (0.9762–0.9877)	0.9348 (0.9135 - 0.9543)
Upper GI	0.2017 (0.1901–0.2137)	0.9880 (0.9806–0.9946)	0.9580 (0.9323 - 0.9804)
Gynaecological	0.2040 (0.1871–0.2209)	0.9895 (0.9799–0.9979)	0.9718 (0.9462 - 0.9942)
Urological	0.2002 (0.1864–0.2141)	0.9525 (0.9358–0.9680)	0.9681 (0.9568 - 0.9785)
Lung	0.2031 (0.1704–0.2331)	0.9630 (0.9281–0.9924)	0.9673 (0.9364 - 0.9933)
Haematological	0.2095 (0.1694–0.2542)	0.9375 (0.8795–0.9868)	0.9697 (0.9408 - 0.9938)
Head and Neck	0.2001 (0.1862–0.2139)	0.9748 (0.9623–0.9858)	0.9267 (0.8917 - 0.9580)
Skin	0.2002 (0.1868–0.2130)	0.9406 (0.9232–0.9570)	0.9609 (0.9493 - 0.9717)

For peer review only

Table 6: 90% Cancer rule-in

Pathway	Proportion of non-cancers ruled-out (i.e. not red-flagged) (specificity) (95% CI)	Positive Predictive Value (95% CI)
Breast	0.4582 (0.4450–0.4715)	0.0890 (0.0793 - 0.0991)
Lower GI	0.2723 (0.2637–0.2811)	0.0642 (0.0587 - 0.0697)
Upper GI	0.3363 (0.3227–0.3503)	0.0732 (0.0644 - 0.0822)
Gynaecological	0.4674 (0.4473–0.4879)	0.1134 (0.0972 - 0.1303)
Urological	0.3548 (0.3379–0.3710)	0.3044 (0.2878 - 0.3208)
Lung	0.3625 (0.3238–0.3987)	0.2541 (0.2178 - 0.2906)
Haematological	0.4330 (0.3807–0.4849)	0.4249 (0.3722 - 0.4759)
Head and Neck	0.2733 (0.2579–0.2885)	0.0804 (0.0703 - 0.0911)
Skin	0.3905 (0.3745–0.4068)	0.3230 (0.3067 - 0.3392)

For peer review only

4 Discussion

Summary of main findings

The NHS 2WW pathways are a major route through which symptomatic patients in the UK are assessed for possible cancer diagnoses. These pathways have been very successful in helping contribute to earlier cancer detection, but the number of 2WW referrals has doubled over the last decade and this has placed a major strain on diagnostic services. These challenges have been exacerbated by the current COVID-19 crisis, with the NHS capacity to assess 2WW referrals reduced, and a backlog of referrals continuing to build.

New diagnostic technologies have the potential to play a role in solving this challenge. This paper reports the development and validation of a set of statistical machine learning algorithms based on routine laboratory blood measurements that can predict cancer outcomes for symptomatic patients referred urgently from primary care for possible cancer diagnosis.

Each algorithm is trained and validated as a test to provide decision support for one of the nine NHS 2WW pathways. Each test produces a calibrated probability that the patient on that 2WW pathway has any type of cancer. These calibrated probabilities can be used in a range of clinical contexts; in this paper we consider two principal use-cases. In use-case 1, the tests are used to rule-out patients whose risk of cancer is very low, allowing clinicians to identify patients for whom investigations of possible non-cancer causes of their symptoms might be more appropriate. In use-case 2, higher-risk patients are red-flagged so that their onwards journey through the 2WW pathway can be expedited.

The main findings of this work are that it is possible to combine a panel of widely available blood markers to produce effective blood tests for cancer for NHS 2WW patients. Such tests are affordable, and can be deployed rapidly to any NHS pathology laboratory with no additional hardware requirements.

4.1 Discussion of main findings within the context of the literature

This work is novel, innovative, and potentially of huge importance for the management of patients referred urgently for suspected cancer. The tests are based upon a panel of routine blood measurements that: are already in common usage in NHS laboratories; work across a range of cancers; can easily be integrated with existing NHS systems. The tests have already been integrated with Mid-Yorkshire Hospitals NHS Trust Laboratory systems.

The tests can both identify patients at higher risk of cancer, such that they can be prioritised for assessment and diagnostic investigations, while also identifying a significant proportion of patients at very low risk who may not need further investigation for suspected cancer. Patients in both groups stand to benefit, either from expedited testing, or from not being exposed to iatrogenic harm and unnecessary cancer worries. The tests can be set at different thresholds in different cancers and within different health settings, making them responsive to local needs, capacity and priorities. COVID has reduced diagnostic capacity and efficiency, this test could be an effective and rapid solution at this time of crisis.

An important practical note is that the criteria for 2WW changed in 2015, reducing the risk threshold warranting an urgent referral from 5% PPV to 3% PPV (i.e. towards the end of the development cohort timeframe). The validation results therefore encompass this change in clinical practice, suggesting a certain robustness to those results.

Strengths

This work is based on well-validated, low-cost clinical assays (see Table S5) already available at scale in NHS pathology laboratories. The tests could therefore be deployed across the UK very rapidly,

For peer review only - http://bmjopen.bmj.com/site/about/guidelines.xhtml

with no additional hardware requirements. These tests are CE marked and are currently undergoing service evaluation in the West Yorkshire and Harrogate Cancer Alliance. The use of low-cost assays means that these tests are very affordable in comparison to typical per-patient 2WW referral costs.[15]

The performance estimates are conservative due to missing data and the historical nature of the blood measurements; prospective evaluation will not suffer from these drawbacks. Even biomarkers with limited individual performance are of value in this approach if they contribute complementary information. The algorithms are designed to be flexible, allowing thresholds to be changed according to clinical need, for example Use-Case 2 during the COVID-19 pandemic. The large numbers reported, the robust analysis and reporting in line with TRIPOD and PROBAST.[11,16] There is the potential to improve performance using the pipeline of new biomarkers being developed for diagnostic, predictive or prognostic purposes.

For peer review only

Limitations

The development and validation was done only in one centre, albeit a large regional cancer centre. We have also only reported the validation on a retrospective sample - a prospective multi-centre evaluation is needed to provide confidence in the generalisability of the model.

We note that the validation set meets the defined sample size criteria (1500 total cases) for 7 of the 9 2WW. 95% CI are provided for all results to make clear the level of uncertainty present due to sample sizes. The remaining (smaller) 2WW pathways as recorded in the clinical data were also considered (Testicular, Brain/CNS, Sarcomas, Children's Cancer, Acute Leukaemia, other cancer), but we did not develop algorithms for these as the available sample sizes were judged too small to train and validate effective models.

There is a possible source of bias, in that the subset of patients who had retrospective blood data may not be representative of the overall 2WW cohort. Different pathways have different conventions as to what blood tests are performed as part of a 2WW referral. For example, we note that the proportion of men with a breast 2WW referral meeting the inclusion criteria (see Table 4) is unusually high compared to that which would be expected for the pathway as a whole. Many breast cancer pathways specifically ask for a panel of blood tests to be performed by GPs prior to two week wait referrals in males (for the investigation of gynaecomastia) which is not required for female referrals, suggesting bias.

We note that differences in the blood tests GPs are likely to provide in the lead up to/as part of a 2WW referral typically vary significantly depending on pathway. This is likely to be an important factor in explaining the difference in patient inclusion rates for each pathway we see for this work (see Tables 1 and 2).

The choice to use blood measurements from up to 90 days prior to and up to 14 days post-referral is also a possible source of bias. Bloods taken significantly before referral can be biased because if the patient does have cancer, any tumour could be smaller or even not yet present at the time the blood test was administered. And bloods taken post-referral begin to run the risk that the decision was taken to order the blood test using information not available at the time of referral. We have chosen this timeframe as a reasonable balance between missing data and these potential biases. We note that for both values (90 days prior, 14 days post) we performed a sensitivity analysis during

algorithm development where we varied these parameters and re-ran otherwise identical cross-validations. This showed that the choice of (90 days prior, 14 days post) was reasonably stable, and in particular we did not see any significant gains in algorithm performance unless the post-referral cut-off was increased past 21 days, suggesting that while that source of bias does exist, it is not a significant factor with a 14 days post-referral cut-off.

4.2 Implications for policy research and practice

Until we have undertaken a prospective evaluation of the performance of the algorithms it is not possible to predict how this will be used. However, we do envisage use of the tool, as part of clinical triage, to both prioritise those at higher levels of risk and de-prioritise those at the very lowest levels of risk, in conjunction with appropriate safety netting. We also need to fully understand the views of patients, clinicians, and commissioners on the acceptability and utility of the tests. We note that each 2WW pathway is distinct, with its own challenges and priorities, as well as differing prevalences of cancer (see e.g. Smith et al [17]) - these issues will likely require detailed consideration by all the key stakeholders on a pathway-by-pathway basis.

The 2WW pathways are an effective and well-used route for earlier cancer diagnosis in the NHS. However, the pressures resulting from this increased use and the current COVID-19 crisis mean that business-as-usual is no longer an option, and the NHS must adapt. New diagnostic technologies can be a part of this solution, giving clinicians better tools with which to triage patients and facilitate appropriate onward journeys through the healthcare system.

Acknowledgements

We thank the following people and organisations for their contributions:

- Emmylou Laird and Claire Eckert for their help with numerous aspects of this work
- The Leeds Institute for Data Analytics, in particular Phil Chambers, for all their support in providing secure computing facilities.
- The pathology and health informatics staff of Leeds Teaching Hospitals Trust and Mid-Yorkshire Hospitals NHS Trust for their help.
- The X-Lab team for help with deployment into the NHS
- The board members and partner organisations of the Leeds Centre for Personalised Medicine and Health and the Leeds Academic Health Partnership.
- The NIHR Leeds MIC and CRUK CANTEST PPI groups, especially Pete Whetstone and Margaret Johnson.
- We thank HealthWatch Leeds and Healthwatch Kirklees for patient consultation forums

Authors' contributions

RS, MM, RN, GH, RF and SD conceptualised the study, and led on the initial protocol development. GT, RF, NSa, BS and PS contributed towards funding applications and protocol refinement. RS, MN, KL and JS developed the software and algorithms, performed the data analysis and completed the CE marking process, with clinical input from RN, SD, NSh, GH and PS and methodological input from BS, CJ and MM. GH led on the provision of de-identified data, assisted by CJ and RF. RF oversaw project management. All authors contributed to the interpretation of the results, writing of the manuscript and approved the final version.

Ethics statement

For peer review only - http://bmjopen.bmj.com/site/about/guidelines.xhtml

Data for the analysis are retrospective and fully de-identified before being released to the study team. The work was carried out under service evaluation with the formal approval of the Leeds Teaching Hospitals Trust R&I and Data Governance Committee (ref LTHT19020), and with the specific approval of the Trust Caldicott Guardian.

Data availability

The data will not be made available to others, as it is de-identified NHS patient data.

Competing interests

RS, KL, MN, JS, NPS, GT are employed by and are shareholders in PinPoint Data Science Ltd. MM has been employed as a consultant to PinPoint Data Science Ltd in October to November 2020. Both the University of Leeds and Leeds Teaching Hospitals Trust have a royalty agreement with PinPoint Data Science Ltd, meaning that those institutions are likely to benefit financially in the event of PinPoint being commercially successful.

Funding information

Aspects of this work have been supported by awards from MRC 'Proximity to Discovery' [MC_PC_17193], Local Enterprise Partnership [Leeds, 109550], and Innovate UK [33772]. Richard Neal, Bethany Shinkins, Geoff Hall and Michael Messenger are funded by the NIHR Leeds In Vitro Diagnostic Co-operative [MIC-2016-015]. PinPoint Data Science Ltd funded the data science work and time contributions of Richard Savage, Matt Neal, Kat Lloyd, Jim Skinner, Giles Tully, Nigel Sansom, and Rosie Ferguson. This research is linked to the CanTest Collaborative, which is funded by Cancer Research UK [C8640/A23385], of which RDN is an Associate Director, MM was a member of Senior Faculty, and BS was part-funded

TRIPOD

This work is reported in accordance with the TRIPOD statement.

Figure 1 caption:

We note that the development set analysed numbers of data points (bottom left) are the same for all pathways with the exception of breast. We discovered during development that modest performance gains could be achieved by using just the 2WW breast pathway data for the breast algorithm, and using the data for all other pathways for each of the other 8 algorithms (hence the same training data were used for all pathways except breast).

Figure 2 caption:

Figure 2 shows stratification of patients on the 2WW breast pathway using the relevant algorithm presented in this work, compared to the standard care pathway. Given an urgent care pathway where the number of referrals exceeds the pathway capacity to see patients within two weeks, use of the test to stratify patients into risk categories (right) leads to a larger proportion of patients with cancer being seen when compared to the standard care pathway (left), in which patients are seen on a first-come, first-served basis. Patients highlighted in red are identified as being at high-risk for cancer (red-flagged), so can be expedited for further diagnostic testing. Patients highlighted in green are identified as being at very low risk for cancer (green-flagged), allowing for initial management in primary care rather than immediate referral to secondary care.

The sliders on the left-hand side show the number of referrals, the number of patients that the pathway can handle in a given time-frame (the pathway capacity), the percentage of cancers which are green-flagged (i.e. setting a very low false negative rate, and therefore high sensitivity c.f. Table 5), and the percentage of cancers that are red-flagged (i.e. identifying cases with high-risk, so that

For peer review only

they can be expedited for further diagnostic testing). The red-flagging slider effectively sets a sensitivity for the red-flagging process; setting sensitivity=0.9 corresponds to the results shown in Table 6. The slider for 'percentage of cancers green-flagged' can be used to set the false negative rate and see the resulting performance of the test. Collectively, this represents a possible approach to using the algorithms to improve the triage of patients referred to a 2WW pathway. An interactive version of this is available at https://www.pinpointdatascience.com/patient-test-stratification

We note that for the standard care pathway, all non-cancer patients are labelled in the same colour (yellow) to indicate that they are unstratified by the test.

Bibliography

1. Suspected Cancer: Recognition and Referral. [Internet]. National Institute for Health and Care Excellence; 2015 [cited 2020 Jul 30]. Available from: www.nice.org.uk/guidance/ng12

2. Round T, Gildea C, Asworth M, Moller H. Association between use of urgent suspected cancer referral and mortality and stage at diagnosis: a 5-year national cohort study. Br J Gen Pract. 2020;70:e389–98.

3. Cancer Waiting Time Statistics. [Internet]. NHS England; Available from: www.england.nhs.uk/statistics/statistical-work-areas/cancer-waiting-times

4. Lai AG, Pasea L, Banerjee A, Denaxas S, Katsoulis M, Chang WH, et al. Estimating excess mortality in people with cancer and multimorbidity in the COVID-19 emergency [Internet]. Oncology; 2020 Jun [cited 2020 Sep 25]. Available from: http://medrxiv.org/lookup/doi/10.1101/2020.05.27.20083287

5. Khan N, Jones D, Grice A, Alderson S, Bradley S, Carder P, et al. A brave new world: the new normal for general practice after the COVID-19 pandemic. BJGP Open. 2020 Jun 2;bjgpopen20X101103.

6. Cree IA, Uttley L, Buckley Woods H, Kikuchi H, Reiman A, et al. The evidence base for circulating tumour DNA blood-based biomarkers for the early detection of cancer: a systematic mapping review. BMC Cancer. 2017 Dec;17(1):697.

7. Liu MC, Oxnard GR, Klein EA, Swanton C, Seiden MV, Cummings SR, et al. Sensitive and specific multi-cancer detection and localization using methylation signatures in cell-free DNA. Ann Oncol. 2020 Jun;31(6):745–59.

8. Ulz P, Perakis S, Zhou Q, Moser T, Belic J, Lazzeri I, et al. Inference of transcription factor binding from cell-free DNA enables tumor subtype prediction and early detection. Nat Commun. 2019 Dec;10(1):4666.

9. Cohen JD, Li L, Wang Y, Thoburn C, Afsari B, Danilova L, et al. Detection and localization of surgically resectable cancers with a multi-analyte blood test. Science. 2018 Feb 23;359(6378):926–30.

10. Badrick E, Cresswell K, Ellis P, Renehan AG, Crosbie EJ, Crosbie P, et al. Top ten research priorities for detecting cancer early. Lancet Public Health. 2019 Nov;4(11):e551.

For peer review only - http://bmjopen.bmj.com/site/about/guidelines.xhtml

11. Collins GS, Reitsma JB, Altman DG, Moons KG. Transparent Reporting of a Multivariable Prediction Model for Individual Prognosis or Diagnosis (TRIPOD) The TRIPOD Statement. Circulation. 2015 Jan 13;131(2):211-9.

12. Platt, J Probabilistic outputs for support vector machines and comparisons to regularized likelihood methods. Advances in Large Margin Classifiers. 1999 10 (3): 61–74.

13. XGBoost documentation https://xgboost.readthedocs.io/en/stable/

14. LightGBM documentation https://lightgbm.readthedocs.io/en/latest/

15. CRUK report, Saving Lives, Averting Costs, 2014, https://www.cancerresearchuk.org/sites/default/files/saving_lives_averting_costs.pdf

16. Wolff RF, Moons KG, Riley RD, Whiting PF, Westwood M, Collins GS, Reitsma JB, Kleijnen J, Mallett S. PROBAST: a tool to assess the risk of bias and applicability of prediction model studies. Annals of internal medicine. 2019 Jan 1;170(1):51-8.

17. Smith L Sansom N Hempihill S Bradley S Shinkins B Wheatstone P Hamilton W Neal R. Trends and variation in urgent referrals for suspected cancer 2009/10 - 2019/20. Accepted at British Journal of General Practice

For peer review only

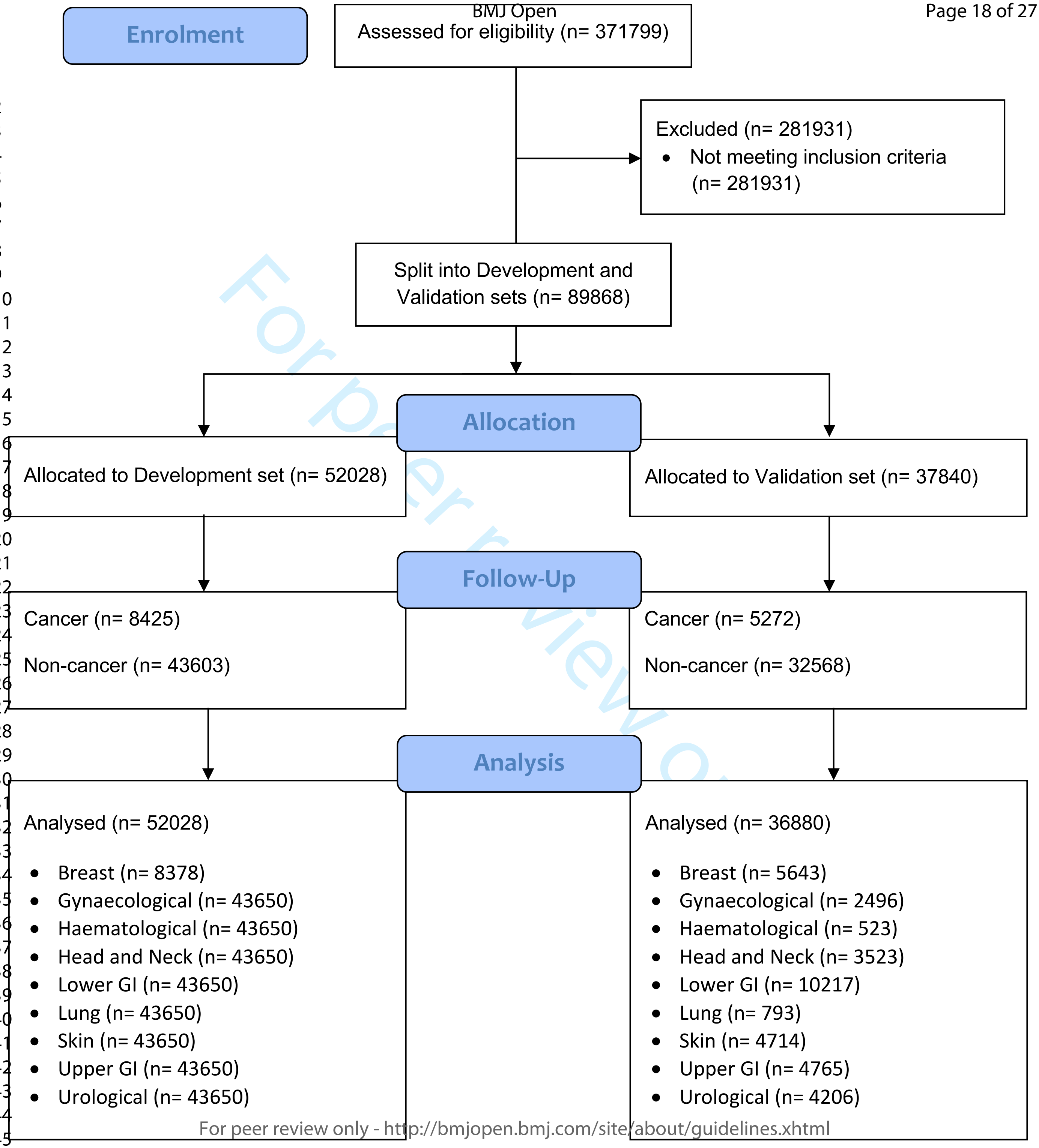

For peer review only - http://bmjopen.bmj.com/site/about/guidelines.xhtml

(Diagram adapted from CONSORT 2010 flow diagram, http://www.consort-statement.org/consort-statement/flow-diagram)

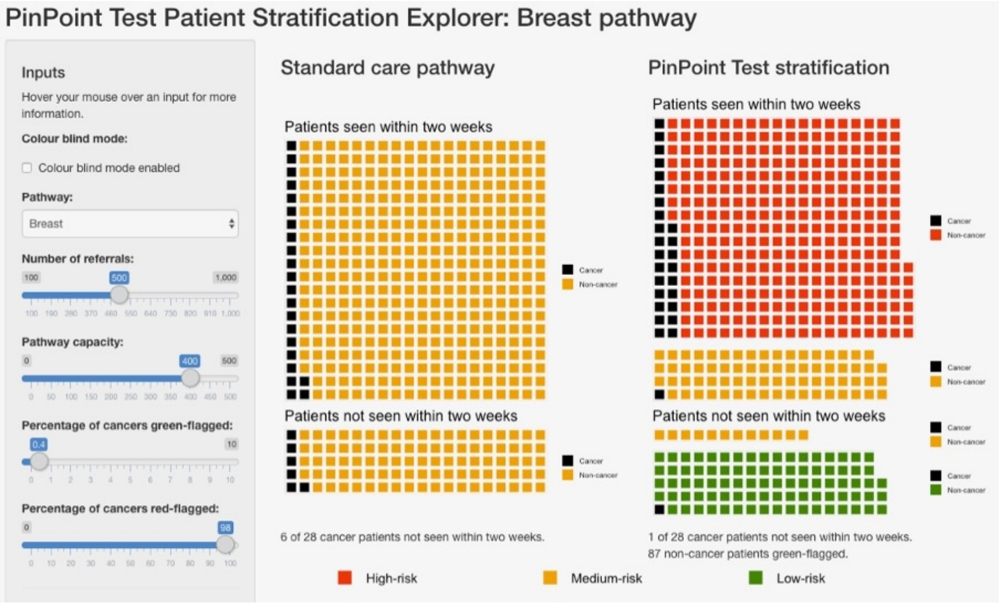

Figure 2 shows stratification of patients on the 2WW breast pathway using the relevant algorithm presented in this work, compared to the standard care pathway. Given an urgent care pathway where the number of referrals exceeds the pathway capacity to see patients within two weeks, use of the test to stratify patients into risk categories (right) leads to a larger proportion of patients with cancer being seen when compared to the standard care pathway (left), in which patients are seen on a first-come, first-served basis. Patients highlighted in red are identified as being at high-risk for cancer (red-flagged), so can be expedited for further diagnostic testing. Patients highlighted in green are identified as being at very low risk for cancer (green-flagged), allowing for initial management in primary care rather than immediate referral to secondary care.

The sliders on the left-hand side show the number of referrals, the number of patients that the pathway can handle in a given time-frame (the pathway capacity), the percentage of cancers which are green-flagged (i.e. setting a very low false negative rate, and therefore high sensitivity c.f. Table 5), and the percentage of cancers that are red-flagged (i.e. identifying cases with high-risk, so that they can be expedited for further diagnostic testing). The red-flagging slider effectively sets a sensitivity for the red-flagging process; setting sensitivity=0.9 corresponds to the results shown in Table 6. The slider for 'percentage of cancers green-flagged' can be used to set the false negative rate and see the resulting performance of the test. Collectively, this represents a possible approach to using the algorithms to improve the triage of patients referred to a 2WW pathway. An interactive version of this is available at https://www.pinpointdatascience.com/patient-test-stratification

We note that for the standard care pathway, all non-cancer patients are labelled in the same colour (yellow) to indicate that they are unstratified by the test.

159x96mm (144 x 144 DPI)

Supplementary Materials

Table of Contents

Test Performance Characteristics **1**
Table S1 .... *1*
Table S2 .... *2*
Clinical Utility Plots **3**
Figure S1 .... *3*
Calibration **4**
Figure S2 .... *4*
Univariate Analyses **5**
Figure S3 .... *6*
ICD-10 Codes **7**
Table S3 .... *7*
Table S4 .... *7*
Reference Costs **7**
Table S5 .... *7*
Prevalence **8**
Table S6 .... *8*

Test Performance Characteristics

In Tables S1 and S2, the "Threshold" column refers to the probability threshold that is applied to the test result for a given pathway in order to get the test performance characteristics given in the corresponding row of the table.

Table S1: Test validation set performance characteristics. Aim: 20% rule-out

Pathway	Threshold	AUC (95% CI)	NPV (95% CI)	TNR (95% CI)	FNR (95% CI)	Sensitivity (95% CI)	Specificity (95% CI)	PPV (95% CI)
Breast	0.0174	0.8007 (0.7750 – 0.8255)	0.9936 (0.9883 – 0.9981)	0.2036 (0.1926 – 0.2143)	0.0224 (0.0067 – 0.0404)	0.9776 (0.9596 – 0.9933)	0.2036 (0.1926 – 0.2143)	0.0672 (0.0601 – 0.0747)
Lower GI	0.0343	0.6798 (0.6566 – 0.7029)	0.9823 (0.9762 – 0.9877)	0.2002 (0.1921 – 0.2081)	0.0652 (0.0457 – 0.0865)	0.9348 (0.9135 – 0.9543)	0.2002 (0.1921 – 0.2081)	0.0609 (0.0559 – 0.0660)
Upper GI	0.0284	0.7323 (0.7008 – 0.7627)	0.9880 (0.9806 – 0.9946)	0.2017 (0.1901 – 0.2137)	0.0420 (0.0196 – 0.0677)	0.9580 (0.9323 – 0.9804)	0.2017 (0.1901 – 0.2137)	0.0653 (0.0576 – 0.0732)
Gynaecological	0.0392	0.8124 (0.7779 – 0.8459)	0.9895 (0.9799 – 0.9979)	0.2040 (0.1871 – 0.2209)	0.0282 (0.0058 – 0.0538)	0.9718 (0.9462 – 0.9942)	0.2040 (0.1871 – 0.2209)	0.0852 (0.0732 – 0.0980)
Urological	0.1062	0.7590 (0.7414 – 0.7757)	0.9525 (0.9358 – 0.9680)	0.2002 (0.1864 – 0.2141)	0.0319 (0.0215 – 0.0432)	0.9681 (0.9568 – 0.9785)	0.2002 (0.1864 – 0.2141)	0.2751 (0.2609 – 0.2900)
Lung	0.0876	0.7376 (0.6938 – 0.7797)	0.9630 (0.9281 – 0.9924)	0.2031 (0.1704 – 0.2331)	0.0327 (0.0067 – 0.0636)	0.9673 (0.9364 – 0.9933)	0.2031 (0.1704 – 0.2331)	0.2249 (0.1934 – 0.2571)
Haematological	0.111	0.7589 (0.7152 – 0.8006)	0.9375 (0.8795 – 0.9868)	0.2095 (0.1694 – 0.2542)	0.0303 (0.0062 – 0.0592)	0.9697 (0.9408 – 0.9938)	0.2095 (0.1694 – 0.2542)	0.3612 (0.3166 – 0.4068)
Head and Neck	0.0423	0.6996 (0.6649 – 0.7334)	0.9748 (0.9623 – 0.9858)	0.2001 (0.1862 – 0.2139)	0.0733 (0.0420 – 0.1083)	0.9267 (0.8917 – 0.9580)	0.2001 (0.1862 – 0.2139)	0.0755 (0.0657 – 0.0852)
Skin	0.0851	0.7220 (0.7057 – 0.7378)	0.9406 (0.9232 – 0.9570)	0.2002 (0.1868 – 0.2130)	0.0391 (0.0283 – 0.0507)	0.9609 (0.9493 – 0.9717)	0.2002 (0.1868 – 0.2130)	0.2796 (0.2656 – 0.2939)

For peer review only - http://bmjopen.bmj.com/site/about/guidelines.xhtml

Table S2: Test validation set performance characteristics. Aim: 90% rule-in

Pathway	Threshold	AUC (95% CI)	NPV (95% CI)	TNR (95% CI)	FNR (95% CI)	Sensitivity (95% CI)	Specificity (95% CI)	PPV (95% CI)
Breast	0.029	0.8007 (0.7746 – 0.8256)	0.9875 (0.9830 – 0.9916)	0.4582 (0.4450 – 0.4715)	0.0990 (0.0678 – 0.1337)	0.9010 (0.8663 – 0.9322)	0.4582 (0.4450 – 0.4715)	0.0890 (0.0793 – 0.0991)
Lower GI	0.041	0.6798 (0.6565 – 0.7029)	0.9799 (0.9745 – 0.9850)	0.2723 (0.2637 – 0.2811)	0.1006 (0.0754 – 0.1262)	0.8994 (0.8738 – 0.9246)	0.2723 (0.2637 – 0.2811)	0.0642 (0.0587 – 0.0697)
Upper GI	0.041	0.7323 (0.7012 – 0.7625)	0.9831 (0.9763 – 0.9893)	0.3363 (0.3227 – 0.3503)	0.0992 (0.0641 – 0.1389)	0.9008 (0.8611 – 0.9359)	0.3363 (0.3227 – 0.3503)	0.0732 (0.0644 – 0.0822)
Gynaecological	0.05	0.8124 (0.7768 – 0.8462)	0.9828 (0.9746 – 0.9900)	0.4674 (0.4473 – 0.4879)	0.1073 (0.0640 – 0.1553)	0.8927 (0.8447 – 0.9360)	0.4674 (0.4473 – 0.4879)	0.1134 (0.0972 – 0.1303)
Urological	0.148	0.7590 (0.7417 – 0.7762)	0.9191 (0.9035 – 0.9336)	0.3548 (0.3379 – 0.3710)	0.0996 (0.0818 – 0.1183)	0.9004 (0.8817 – 0.9182)	0.3548 (0.3379 – 0.3710)	0.3044 (0.2878 – 0.3208)
Lung	0.134	0.7376 (0.6939 – 0.7796)	0.9431 (0.9120 – 0.9702)	0.3625 (0.3238 – 0.3987)	0.0915 (0.0482 – 0.1392)	0.9085 (0.8608 – 0.9518)	0.3625 (0.3238 – 0.3987)	0.2541 (0.2178 – 0.2906)
Haematological	0.189	0.7589 (0.7143 – 0.7999)	0.9118 (0.8633 – 0.9509)	0.4330 (0.3807 – 0.4849)	0.0909 (0.0506 – 0.1412)	0.9091 (0.8588 – 0.9494)	0.4330 (0.3807 – 0.4849)	0.4249 (0.3722 – 0.4759)
Head and Neck	0.047	0.6996 (0.6648 – 0.7339)	0.9751 (0.9644 – 0.9847)	0.2733 (0.2579 – 0.2885)	0.0991 (0.0619 – 0.1393)	0.9009 (0.8607 – 0.9381)	0.2733 (0.2579 – 0.2885)	0.0804 (0.0703 – 0.0911)
Skin	0.141	0.7220 (0.7060 – 0.7380)	0.9236 (0.9100 – 0.9367)	0.3905 (0.3745 – 0.4068)	0.0999 (0.0829 – 0.1175)	0.9001 (0.8825 – 0.9171)	0.3905 (0.3745 – 0.4068)	0.3230 (0.3067 – 0.3392)

For peer review only

For peer review only - http://bmjopen.bmj.com/site/about/guidelines.xhtml

Clinical Utility Plots

Figure S1 shows negative predictive value (NPV) against the specificity, i.e. the proportion of patients ruled out, for each pathway. This shows the trade-off for a given pathway between avoiding erroneously ruling out patients who in fact have cancer (high NPV is better) vs the proportion of patients referred who are ruled out of the pathway.

Bootstrap resampling with replacement with 1000 bootstraps was used to generate 95% and 68% confidence intervals on NPV. The solid line marks the median, the dark grey band indicates the 68% confidence interval, and the light grey band indicates the 95% confidence interval.

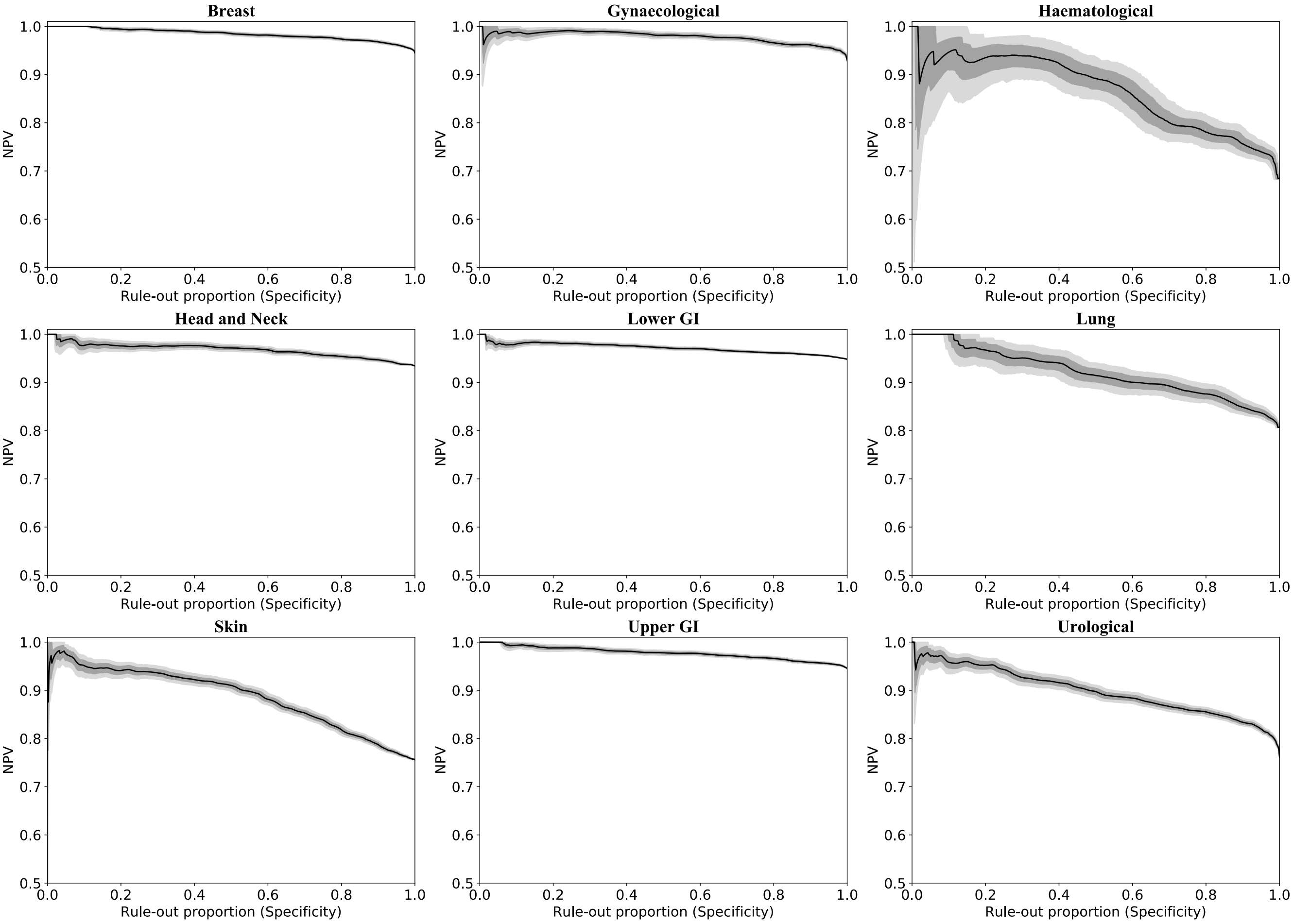

Figure S1: Plots of Negative Predictive Ability against specificity for each pathway. Light and dark grey bands indicate 68% and 95% confidence intervals. See text for details.

For peer review only - http://bmjopen.bmj.com/site/about/guidelines.xhtml

Calibration

Figure S2 shows calibration curves for validation set predictions by the algorithms for each pathway, calculated using equal occupancy bins. Good calibration means that the algorithm results can be interpreted as being the probability of a given patient having cancer and is indicated by the points lying along the dashed diagonal line.

The error bars show the 95% binomial proportion confidence interval, calculated using the Wilson score with continuity correction. The log loss for each pathway is also included.

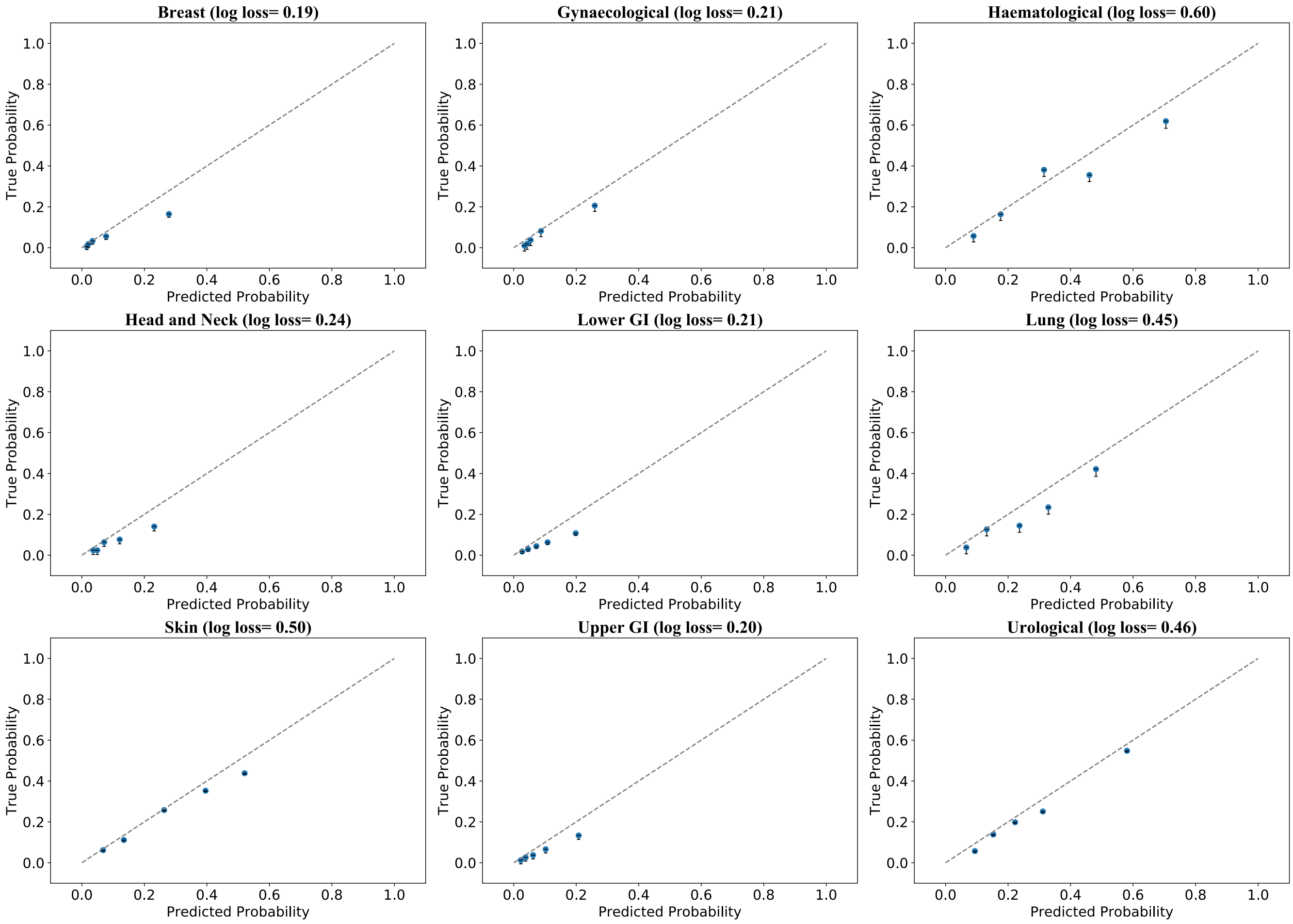

Figure S2: Plots of calibration curves per pathway. Dashed grey line indicates perfect calibration. See text for details.

Univariate Analyses

Validation set predicted probabilities were generated using the nine algorithms. For each input data feature, ROC AUCs were calculated for cases restricted to those for which the feature data was available, whereby the feature was used as the predictor and the binary cancer flag as the outcome. ROC AUCs were also calculated using the probabilities predicted by the algorithm, with identical restriction of cases applied to allow direct comparison. The difference between the algorithm ROC AUC and the single-feature ROC AUC was then calculated for each feature, ΔAUC.

Using this process, ΔAUCs were calculated for each feature and each pathway-specific algorithm. Bootstrap resampling with replacement with 10000 bootstraps was used to generate 95% confidence intervals on ΔAUC, where both the algorithm ROC AUC and single-feature ROC AUC were calculated on the same bootstrap samples.

Figure S3 shows the median ΔAUCs as black circles with 95% confidence intervals, for each feature and each pathway. Any features with data for less than one hundred patients for a given pathway were removed from the plot for that pathway. Arrows indicate that a confidence interval extends outside the plot area, in the direction of the arrow. The number of cancers and the number of cases were annotated for each feature at the bottom of the plot area. These are in the format "# cancers/# cases". An asterisk was appended to feature names for which the 95% confidence interval does not intersect the line $\Delta AUC = 0$. The feature names are assigned according to the category into which the blood test falls—"FBC" for blood counts, "Bio" for biochemistry, and "TM" for tumour markers—with numbers assigned arbitrarily but consistently across the subplots.

For peer review only

For peer review only - http://bmjopen.bmj.com/site/about/guidelines.xhtml

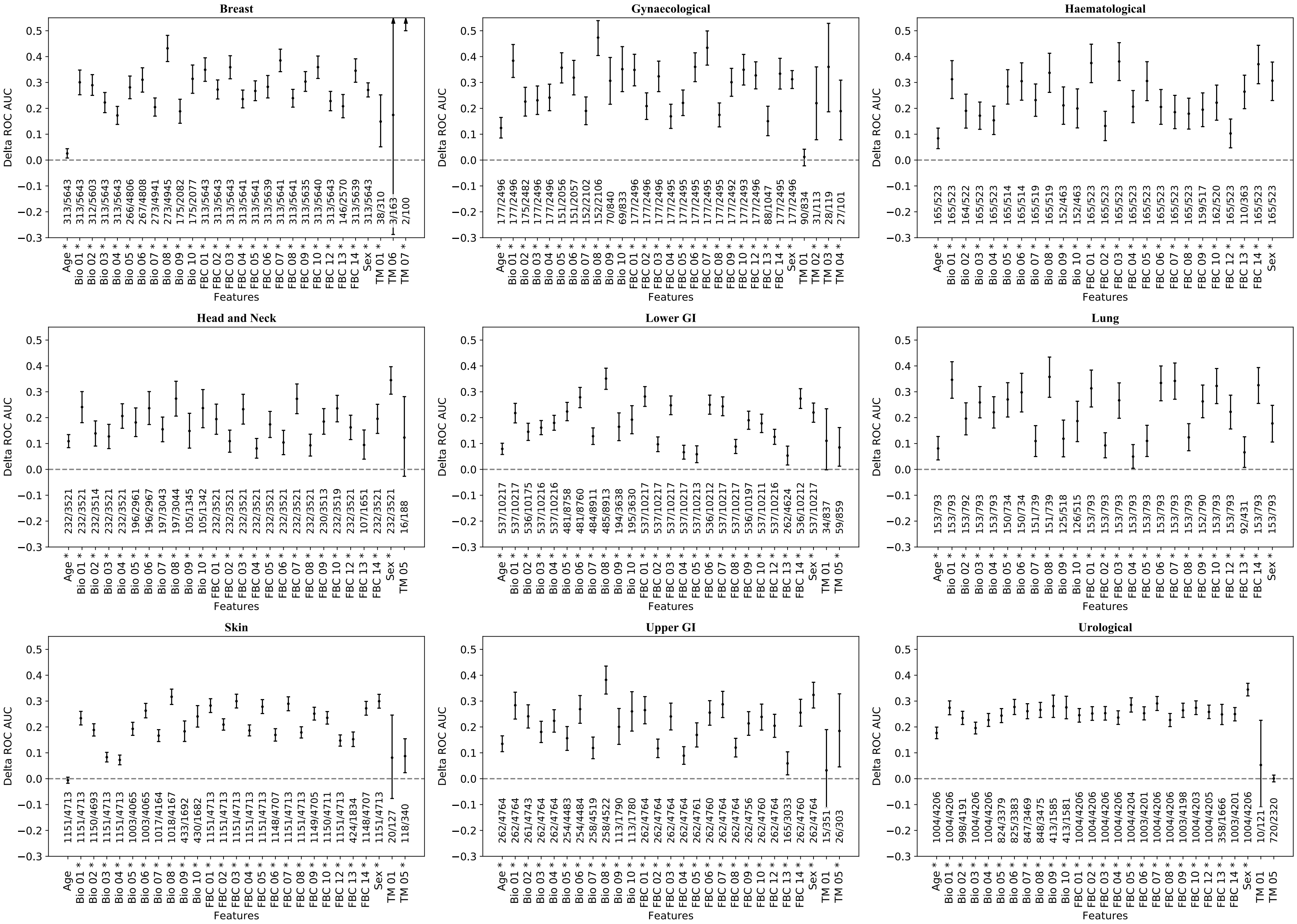

Figure S3: Plots of ΔAUC per feature per pathway. The vertical confidence intervals show the difference between ROC AUC performance for the algorithm and those that one obtains from using an individual analyte. See text for details.

ICD-10 Codes

Table S3: ICD-10 codes designated as "cancer" for the algorithms

ICD-10 code	ICD-10 text
C00-C14	Malignant neoplasms of lip, oral cavity and pharynx
C15-C26	Malignant neoplasms of digestive organs
C30-C39	Malignant neoplasms of respiratory and intrathoracic organs
C40-C41	Malignant neoplasms of bone and articular cartilage
C43-C44	Melanoma and other malignant neoplasms of skin
C45-C49	Malignant neoplasms of mesothelial and soft tissue
C50-C50	Malignant neoplasm of breast
C51-C58	Malignant neoplasms of female genital organs
C60-C63	Malignant neoplasms of male genital organs
C64-C68	Malignant neoplasms of urinary tract
C69-C72	Malignant neoplasms of eye, brain and other parts of central nervous system
C73-C75	Malignant neoplasms of thyroid and other endocrine glands
D00	Carcinoma in situ of oral cavity, oesophagus and stomach
D01	Carcinoma in situ of other and unspecified digestive organs
D02	Carcinoma in situ of middle ear and respiratory system
D03	Melanoma in situ
D04	Carcinoma in situ of skin
D05	Carcinoma in situ of breast
D07	Carcinoma in situ of other and unspecified genital organs
D09	Carcinoma in situ of other and unspecified sites

Table S4: ICD-10 codes designated as "benign" for the algorithms

ICD-10 code	ICD-10 text
D06	Carcinoma in situ of cervix uteri
D10-D36	Benign neoplasms
D37-D48	Neoplasms of uncertain or unknown behaviour

Reference Costs

Table S5 shows the reference costs for the analytes that are used as inputs to the algorithms. These costs, from the 2018-2019 reference schedule, were also used for health economics that have been performed and will be published separately.

Table S5: NHS reference costs, 2018-2019

Item	Category	Cost (2018-19 Ref Schedule)
Full Blood Counts	Haematology	£3.00
Urea & Electrolytes	Clinical Biochemistry	£1.00
CA125	Clinical Biochemistry	£1.00
CA19-9	Clinical Biochemistry	£1.00
Carcinoembryonic Antigen	Clinical Biochemistry	£1.00
CA15-3	Clinical Biochemistry	£1.00
PSA	Clinical Biochemistry	£1.00
Alpha Fetoprotein	Clinical Biochemistry	£1.00
Human Chorionic Gonadotrophin	Clinical Biochemistry	£1.00
C-Reactive Protein	Clinical Biochemistry	£1.00
Liver Function Tests	Clinical Biochemistry	£1.00
Phlebotomy	-	£4.00
**Total NHS Costs**	-	**£17.00**

For peer review only - http://bmjopen.bmj.com/site/about/guidelines.xhtml

Prevalence

Table S6 shows the prevalences, by pathway, for the whole cohort of patients 2011-19, including those excluded from the analyses. A comparison with Table 2 shows differences between the overall prevalences and those for the included patients, highlighting possible sources of spectrum bias. Typical prevalences for the 2WW pathways in NHSE are given for 2009-10 and 2019-20 in Smith et al. [main paper reference 17]. The right hand most column corresponds to the cancer outcomes used in the analyses in this paper, and we note that these are typically somewhat higher than 2WW prevalence rates due to the inclusion of any cancer diagnosis up to 12 months after the referral date. To illustrate this, the middle column shows the cancer prevalence when the diagnoses of the cohort of patients are restricted to only those found via the 2WW pathways, and within 62 days of referral.

Table S6: Cancer prevalence for whole cohort of patients 2011-19, including those excluded from the analyses, for two examples of diagnosis inclusion criterion. See text for details.

Pathway	Cancer prevalence (%) Restricted diagnoses (see text)	Cancer prevalence (%) All diagnoses (see text)
Breast	6.8	8.0
Lower GI	7.1	11.5
Upper GI	10.6	15.4
Gynaecological	11.3	14.3
Urological	25.0	30.6
Lung	30.0	40.4
Haematological	33.1	38.3
Head and Neck	8.8	12.6
Skin	19.4	22.3

For peer review only

For peer review only - http://bmjopen.bmj.com/site/about/guidelines.xhtml

TRIPOD Checklist: Prediction Model Development and Validation

Section/Topic	Item		Checklist Item	Page
**Title and abstract**				
Title	1	D;V	Identify the study as developing and/or validating a multivariable prediction model, the target population, and the outcome to be predicted.	1
Abstract	2	D;V	Provide a summary of objectives, study design, setting, participants, sample size, predictors, outcome, statistical analysis, results, and conclusions.	1
**Introduction**				
Background and objectives	3a	D;V	Explain the medical context (including whether diagnostic or prognostic) and rationale for developing or validating the multivariable prediction model, including references to existing models.	2
	3b	D;V	Specify the objectives, including whether the study describes the development or validation of the model or both.	2
**Methods**				
Source of data	4a	D;V	Describe the study design or source of data (e.g., randomized trial, cohort, or registry data), separately for the development and validation data sets, if applicable.	3
	4b	D;V	Specify the key study dates, including start of accrual; end of accrual; and, if applicable, end of follow-up.	3
Participants	5a	D;V	Specify key elements of the study setting (e.g., primary care, secondary care, general population) including number and location of centres.	3
	5b	D;V	Describe eligibility criteria for participants.	3
	5c	D;V	Give details of treatments received, if relevant.	NA
Outcome	6a	D;V	Clearly define the outcome that is predicted by the prediction model, including how and when assessed.	3
	6b	D;V	Report any actions to blind assessment of the outcome to be predicted.	3
Predictors	7a	D;V	Clearly define all predictors used in developing or validating the multivariable prediction model, including how and when they were measured.	3
	7b	D;V	Report any actions to blind assessment of predictors for the outcome and other predictors.	3
Sample size	8	D;V	Explain how the study size was arrived at.	4
Missing data	9	D;V	Describe how missing data were handled (e.g., complete-case analysis, single imputation, multiple imputation) with details of any imputation method.	4
Statistical analysis methods	10a	D	Describe how predictors were handled in the analyses.	4
	10b	D	Specify type of model, all model-building procedures (including any predictor selection), and method for internal validation.	4
	10c	V	For validation, describe how the predictions were calculated.	4
	10d	D;V	Specify all measures used to assess model performance and, if relevant, to compare multiple models.	4
	10e	V	Describe any model updating (e.g., recalibration) arising from the validation, if done.	4
Risk groups	11	D;V	Provide details on how risk groups were created, if done.	NA
Development vs. validation	12	V	For validation, identify any differences from the development data in setting, eligibility criteria, outcome, and predictors.	4
**Results**				
Participants	13a	D;V	Describe the flow of participants through the study, including the number of participants with and without the outcome and, if applicable, a summary of the follow-up time. A diagram may be helpful.	5
	13b	D;V	Describe the characteristics of the participants (basic demographics, clinical features, available predictors), including the number of participants with missing data for predictors and outcome.	4
	13c	V	For validation, show a comparison with the development data of the distribution of important variables (demographics, predictors and outcome).	6/7
Model development	14a	D	Specify the number of participants and outcome events in each analysis.	6/7
	14b	D	If done, report the unadjusted association between each candidate predictor and outcome.	supp
Model specification	15a	D	Present the full prediction model to allow predictions for individuals (i.e., all regression coefficients, and model intercept or baseline survival at a given time point).	NA
	15b	D	Explain how to the use the prediction model.	NA
Model performance	16	D;V	Report performance measures (with CIs) for the prediction model.	8/9
Model-updating	17	V	If done, report the results from any model updating (i.e., model specification, model performance).	NA
**Discussion**				
Limitations	18	D;V	Discuss any limitations of the study (such as nonrepresentative sample, few events per predictor, missing data).	12
Interpretation	19a	V	For validation, discuss the results with reference to performance in the development data, and any other validation data.	NA
	19b	D;V	Give an overall interpretation of the results, considering objectives, limitations, results from similar studies, and other relevant evidence.	11/12
Implications	20	D;V	Discuss the potential clinical use of the model and implications for future research.	11/12
**Other information**				
Supplementary information	21	D;V	Provide information about the availability of supplementary resources, such as study protocol, Web calculator, and data sets.	supp
Funding	22	D;V	Give the source of funding and the role of the funders for the present study.	4

*Items relevant only to the development of a prediction model are denoted by D, items relating solely to a validation of a prediction model are denoted by V, and items relating to both are denoted D;V. We recommend using the TRIPOD Checklist in conjunction with the TRIPOD Explanation and Elaboration document.

For peer review only - http://bmjopen.bmj.com/site/about/guidelines.xhtml
